# Supplementary material for: Patritumab deruxtecan in HER2-negative breast cancer: part B results of the window-of-opportunity SOLTI-1805 TOT-HER3 trial and biological determinants of early response
Source: Nat Commun. 2024 Jul 11;15:5826. doi: 10.1038/s41467-024-50056-y (PMC11239918; doi:10.1038/s41467-024-50056-y)
Supplement: Supplementary file 1 — Supplementary Information [file 41467_2024_50056_MOESM1_ESM.pdf]

Supplementary information

Patritumab deruxtecan in hormone receptor-positive and HER2-negative (HR+/HER2-) breast cancer: part B results of the window-of-opportunity SOLTI TOT-HER3 trial and biological determinants of early response

Fara Brasó-Maristany, Juan Manuel Ferrero-Cafiero, Claudette Falato, Olga Martínez-Sáez, Juan Miguel Cejalvo, Mireia Margelí, Pablo Tolosa, Francisco Javier Salvador-Bofill, Josefina Cruz, Blanca González-Farré, Esther Sanfeliu, Andreu Òdena, Violeta Serra, Francisco Pardo, Ana María Luna Barrera, Miriam Arumi, Juan Antonio Guerra, Guillermo Villacampa, Rodrigo Sánchez-Bayona, Eva Ciruelos, Martín Espinosa-Bravo, Yann Izarzugaza, Patricia Galván, Judith Matito, Sonia Pernas, Maria Vidal, Anu Santhanagopal, Dalila Sellami, Stephen Esker, Pang-Dian Fan, Fumitaka Suto, Ana Vivancos, Tomás Pascual, Aleix Prat and Mafalda Oliveira

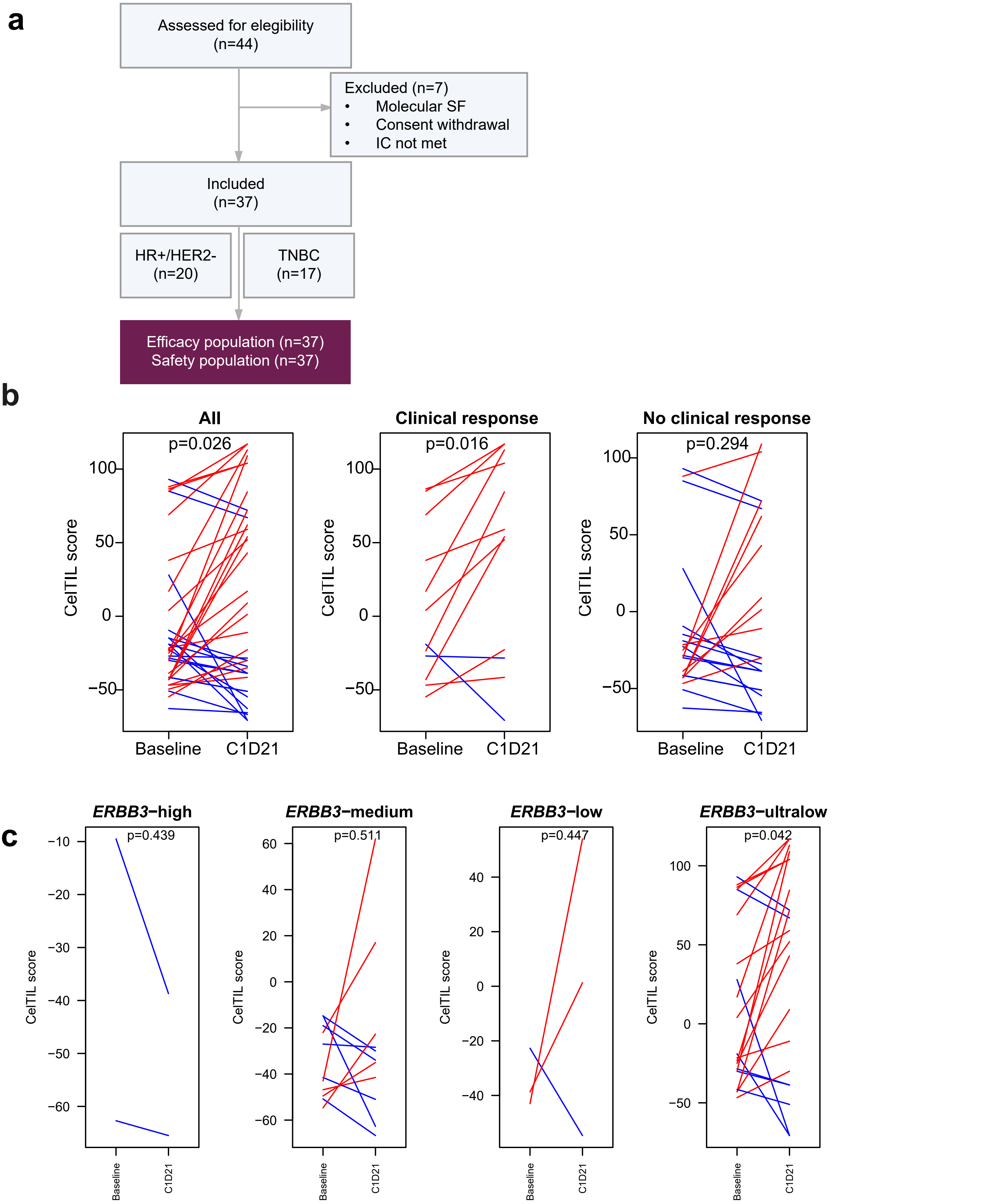

Supplementary Figure 1. Results of SOLTI-1805 TOT-HER3 part B (a) Consort diagram. **(b)** CelTIL score change SOLTI-1805 TOT-HER3 part B in all patients (n=37), in patients with clinical response at day 21 (C1D21) (n=12) and in patients without clinical response (n=22) at day 21. **(c)** CelTIL score change according to *ERBB3* mRNA expression group based on pre-established cutoffs (*ERBB3*-high n=2, *ERBB3*-medium n=11, *ERBB3*-low n=3, *ERBB3* ultralow n=21) (Pascual et al. Front Oncol 2021). In (b) and (c) red lines represent increases at day 21 while blue lines represent decreases at day 21. Source data are provided as a Source Data file.

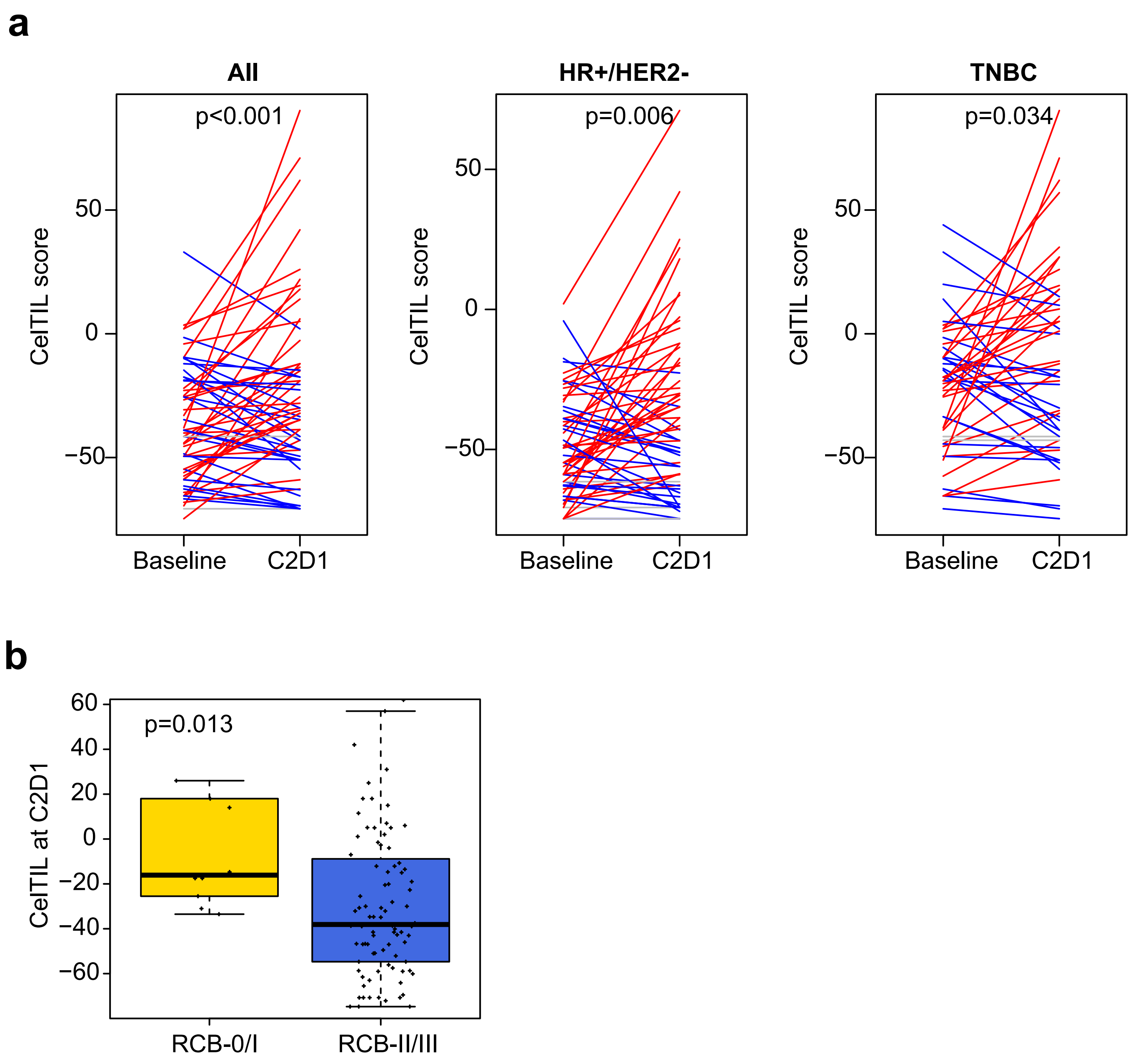

Supplementary Figure 2. Value of CelTIL in patients with HER2-negative breast cancer. (a) CelTIL score change in 132 paired samples of the SOLTI-1007 NeoEribulin trial (Pascual et al. npj breast cancer 2021) in all patients, in patients with HR+/HER2- breast cancer (n=82) and in patients with TNBC (n=50). Red lines represent increases at cycle 2 day 1 (C2D1) while blue lines represent decreases at C2D1. **(b)** Boxplot showing CelTIL score after one cycle of neoadjuvant eribulin in patients with RCB-0/I (n=10) and RCB-II/III (n=88). For the boxplot, center line indicates median; box limits indicate upper and lower quartiles; whiskers indicate 1.5× interquartile range. P-values (p) were determined by two-tailed unpaired t-tests. Source data are provided as a Source Data file.

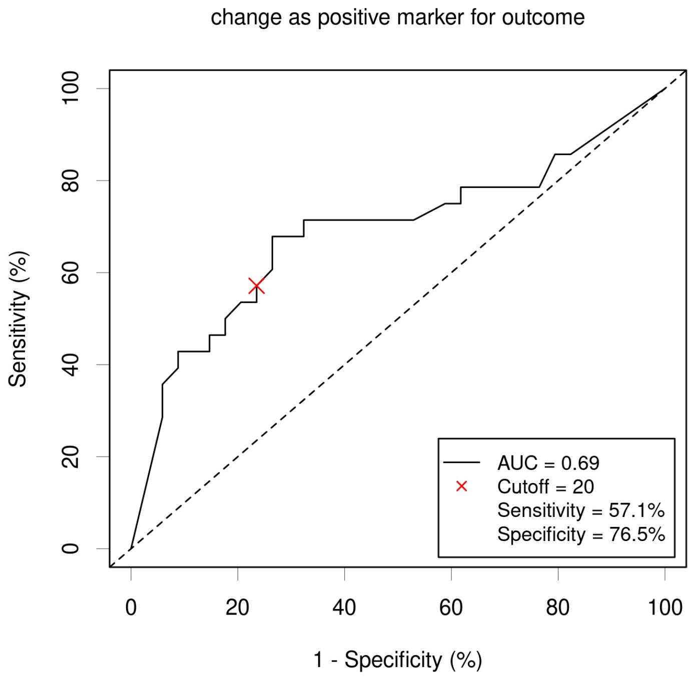

Supplementary Figure 3. Association of CelTIL and clinical response in SOLTI-1805 TOT-HER3 trial. Performance of CelTIL response (as a continuous variable) to predict clinical response. ROC AUC value is reported. Source data are provided as a Source Data file.

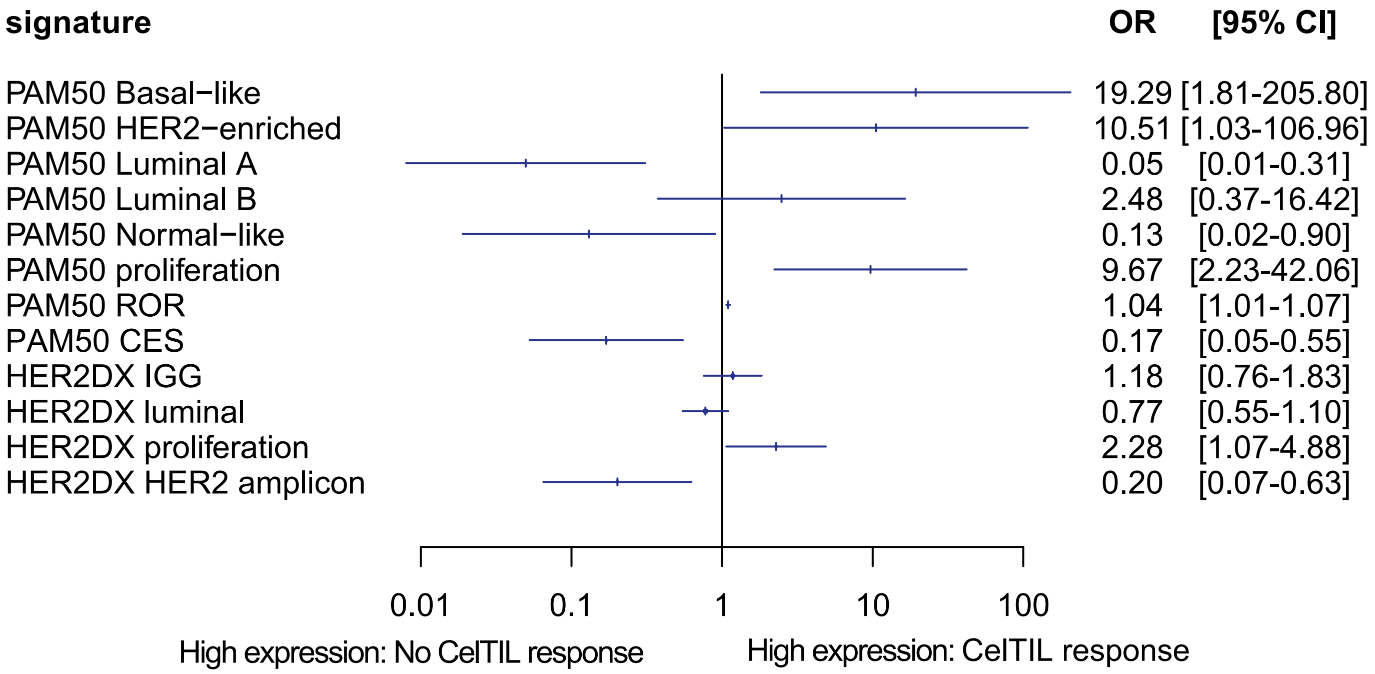

Supplementary Figure 4. Association of gene expression signatures with CelTIL response after one dose of HER3-DXd. Forest plot of the PAM50 and HER2DX gene expression signatures and their association CelTIL response after one dose of HER3-DXd in a logistic regression analysis in patients with CelTIL response (n=26) and without CelTIL response (n=51). Data are presented as the odds ratios (OR) with error bars showing 95% confidence intervals. Source data are provided as a Source Data file.

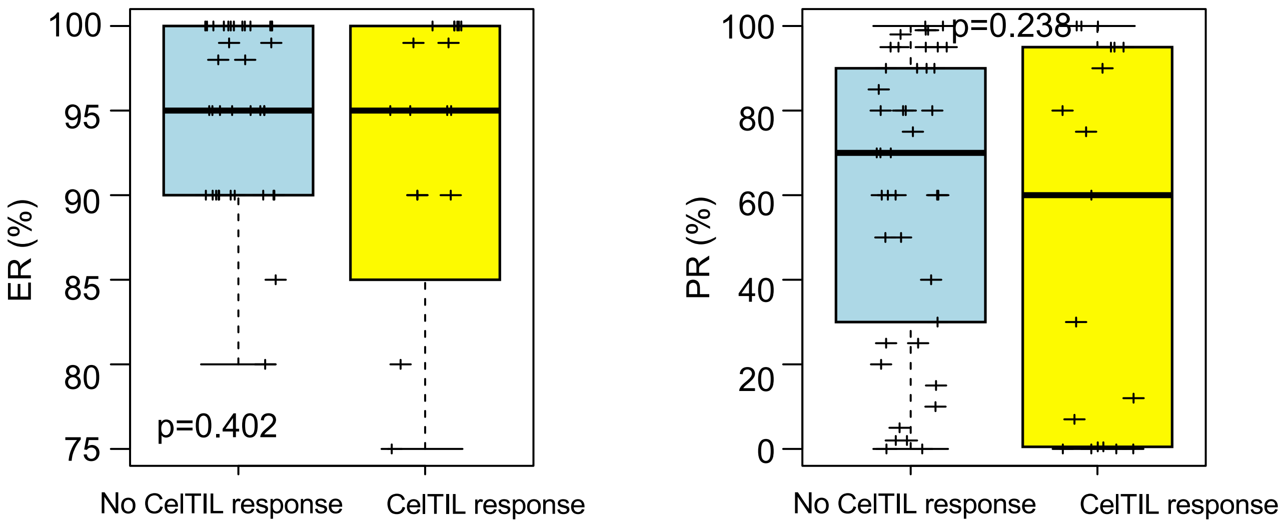

Supplementary Figure 5. Association of ER and PR expression with CelTIL response after one dose of HER3-DXd. Boxplot showing ER (%) and PR (%) in patients with (n=26) and without (n=51) CelTIL response. For the boxplots, center line indicates median; box limits indicate upper and lower quartiles; whiskers indicate 1.5× interquartile range. P-values (p) were determined by two-tailed unpaired t-tests. Source data are provided as a Source Data file.

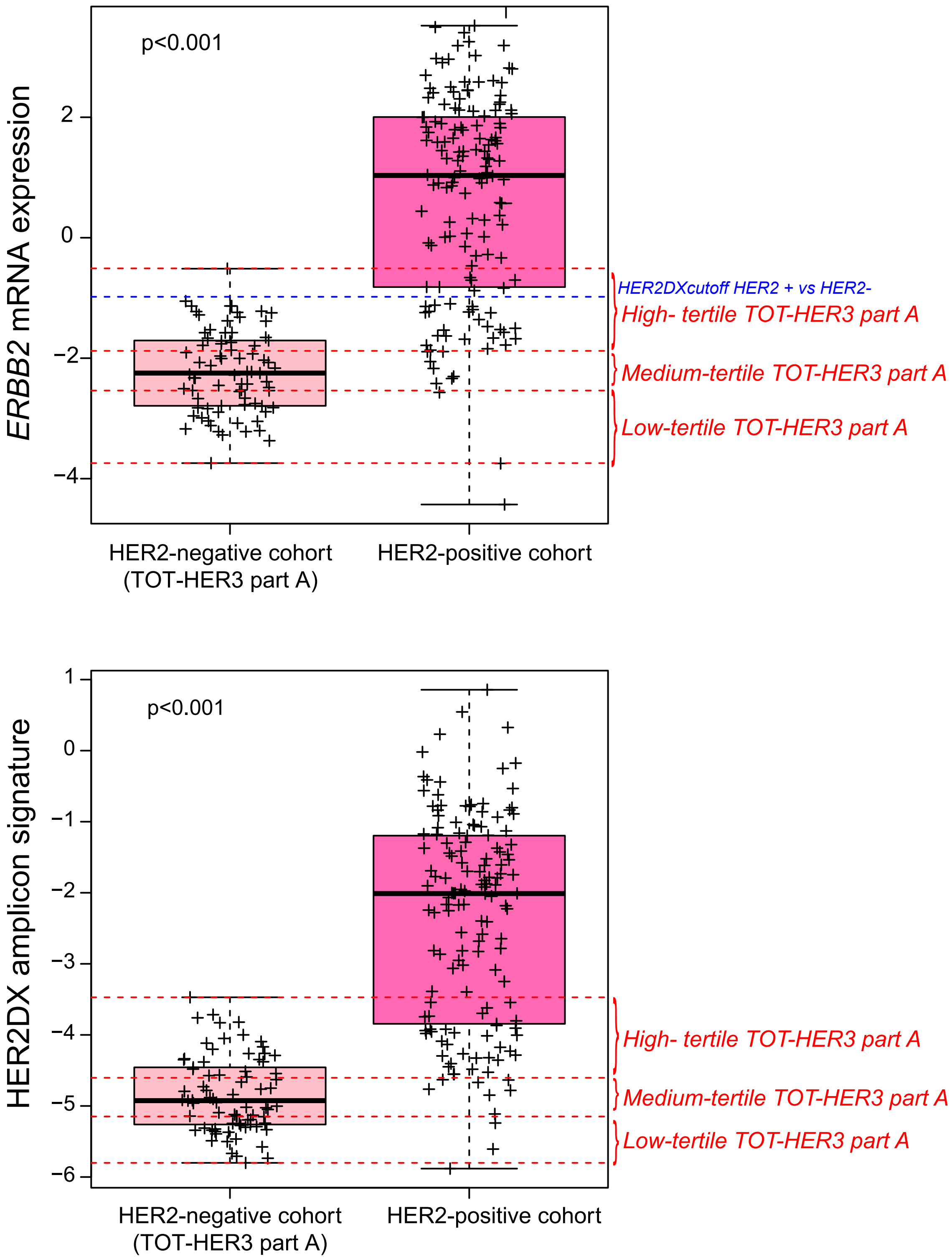

Supplementary Figure 6. Expression of *ERBB2* mRNA and the HER2DX amplicon signature in HER2-negative and HER2-positive tumors. Boxplots of the expression of *ERBB2* mRNA and the HER2DX amplicon signature in the SOLTI-1805 TOT-HER3 part A trial (n=77) and a HER2-positive cohort (n=146) from Hospital Clinic of Barcelona. For the boxplots, center line indicates median; box limits indicate upper and lower quartiles; whiskers indicate 1.5× interquartile range. P-values (p) were determined by two-tailed unpaired t-tests. Source data are provided as a Source Data file.

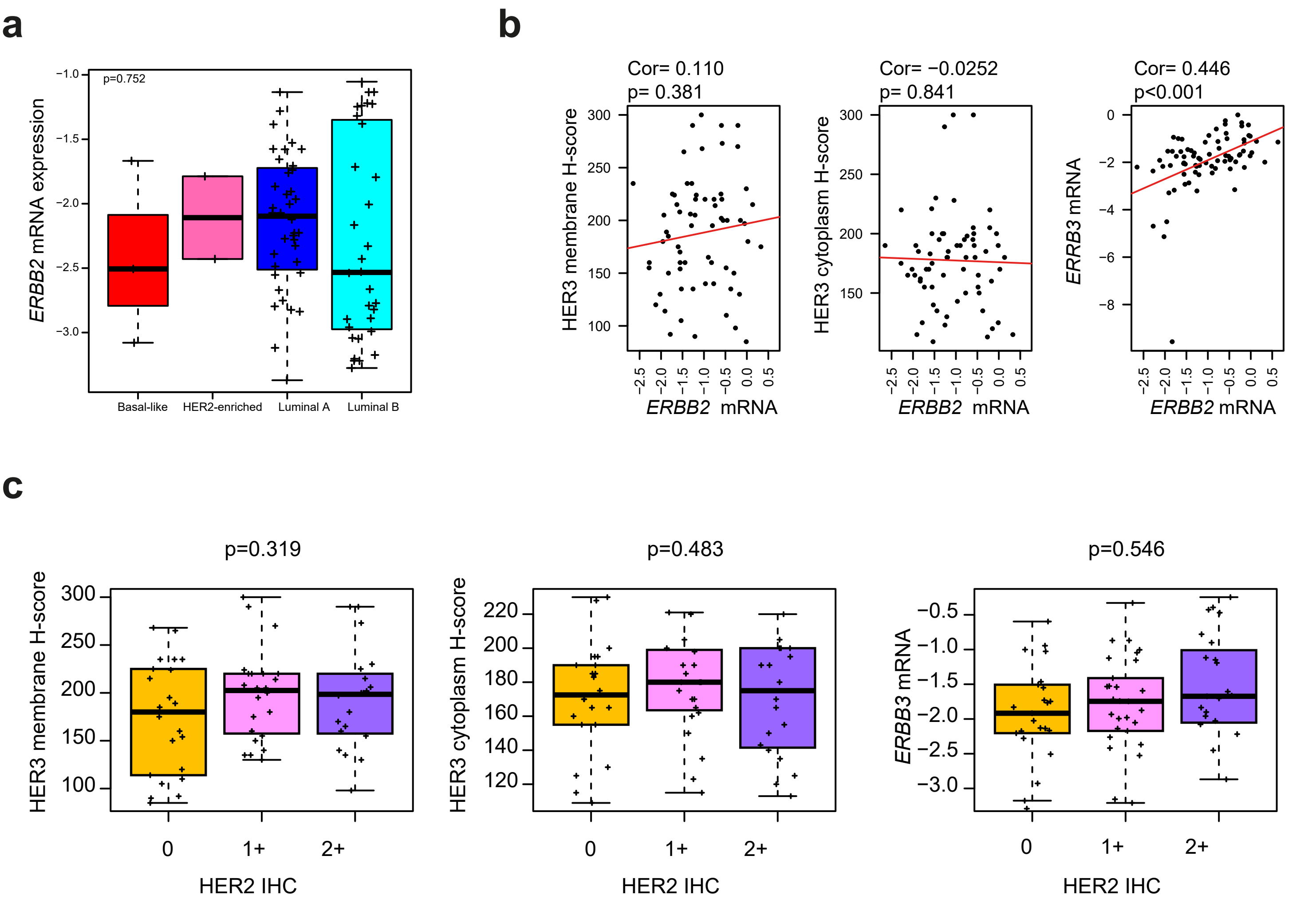

Supplementary Figure 7. Associations between *ERBB2* and PAM50 subtypes and *ERBB3*/HER3 expression. (a) Boxplot showing *ERBB2* mRNA levels across PAM50 molecular subtypes (Basal-like n=3, HER2-enriched n=2, Luminal A n=40, Luminal B n=32). For the boxplot, center line indicates median; box limits indicate upper and lower quartiles; whiskers indicate 1.5× interquartile range.P-value (p) was determined by one-way ANOVA. **(b)** Correlation between *ERBB2* mRNA levels and HER3 membrane protein expression, HER3 cytoplasm protein expression or *ERBB3* mRNA. P-values (p) in were determined by Pearson's correlation. **(c)** Boxplots showing HER3 membrane protein expression, HER3 cytoplasm protein expression or *ERBB3* mRNA across HER2 IHC groups (HER2 0 n=25, HER2 1+ n=29, HER2 2+ n=23). For the boxplots, center line indicates median; box limits indicate upper and lower quartiles; whiskers indicate 1.5× interquartile range. P-values (p) were determined by one-way ANOVA. Source data are provided as a Source Data file.

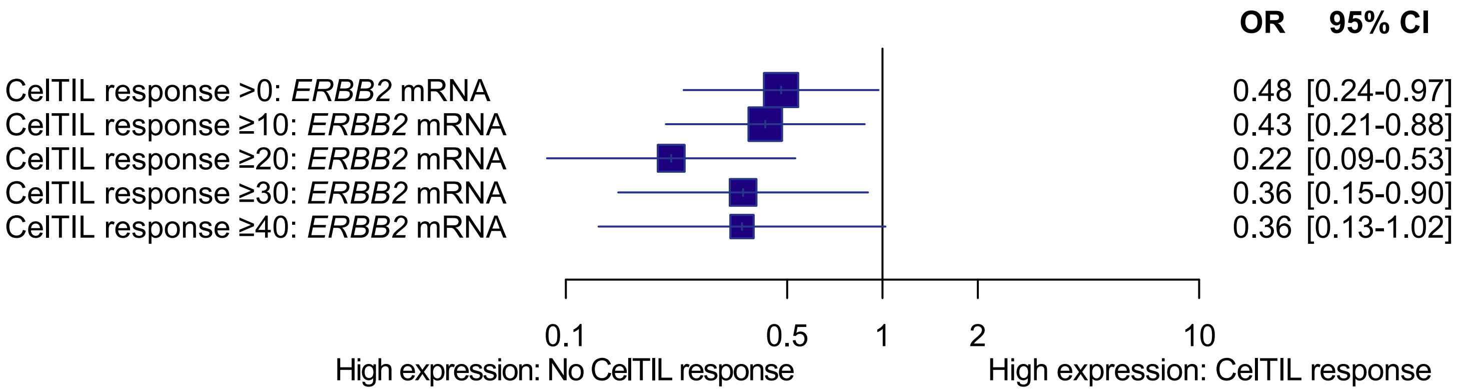

Supplementary Figure 8. Sensitivity analysis using different CelTIL response cutoffs. Forest plot of *ERBB2* mRNA and its association with CelTIL response after one dose of HER3-DXd in a logistic regression analysis using different CelTIL response cutoffs (>0, ≥10, ≥20, ≥30, ≥40 points) in patients with CelTIL response (n=26) and without CelTIL response (n=51). Data are presented as the odds ratios (OR) with error bars showing 95% confidence intervals. Source data are provided as a Source Data file.

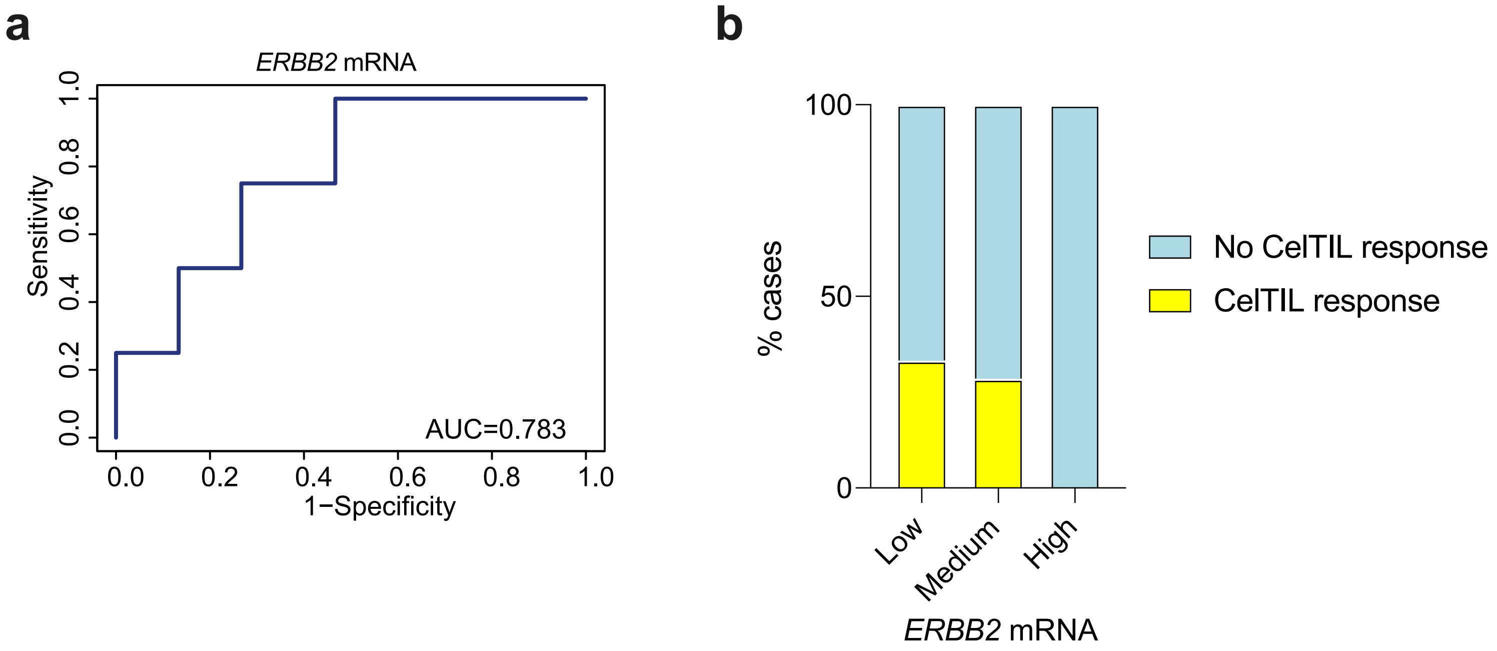

Supplementary Figure 9. High *ERBB2* mRNA is associated with low CelTIL response after one dose of HER3-DXd in TOT-HER3 part B. (a) Performance of *ERBB2* mRNA to predict CelTIL response after one dose of HER3-DXd in 20 patients with HR+/HER2- tumors. ROC AUC values are reported. **(b)** Proportion of tumors with high and low CelTIL response after one dose of HER3-DXd in each *ERBB2* group (as defined by tertiles from part A, low n=6, medium n=7, high n=7). P-values (p) was determined by Fisher's exact test. Source data are provided as a Source Data file.

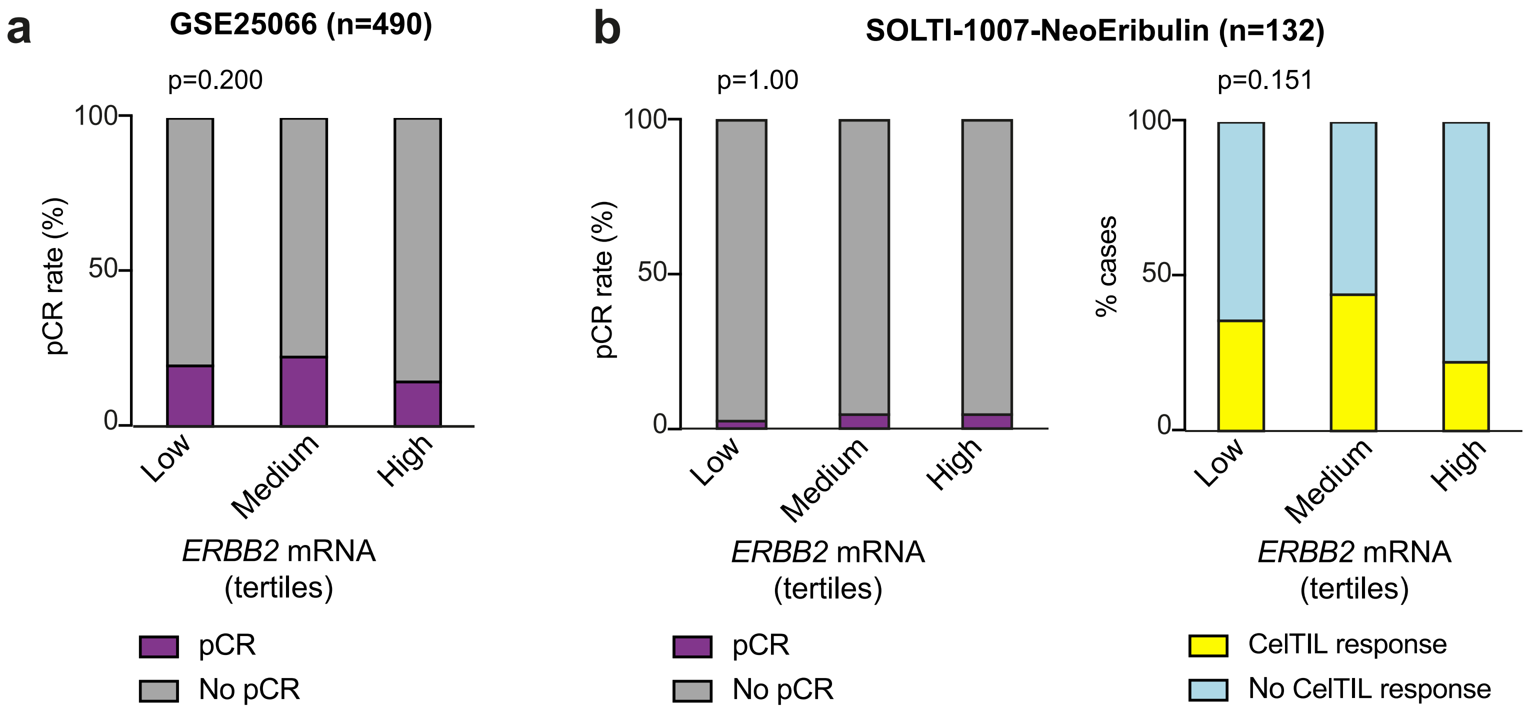

Supplementary Figure 10. Association of *ERBB2* mRNA expression and chemotherapy response. (a) pCR rates in the different *ERBB2* mRNA expression groups defined by tertiles in a publicly available cohort of 490 patients with HER2-negative breast cancer treated with anthracycline/taxane-based neoadjuvant chemotherapy. **(b)** pCR rates and CelTIL response in the different of *ERBB2* mRNA expression groups defined by tertiles in a cohort of 132 patients with HER2-negative breast cancer treated with eribulin in the SOLTI-1007 NeoEribulin neoadjuvant trial. P-values were defined by Fisher's exact test. Source data are provided as a Source Data file.

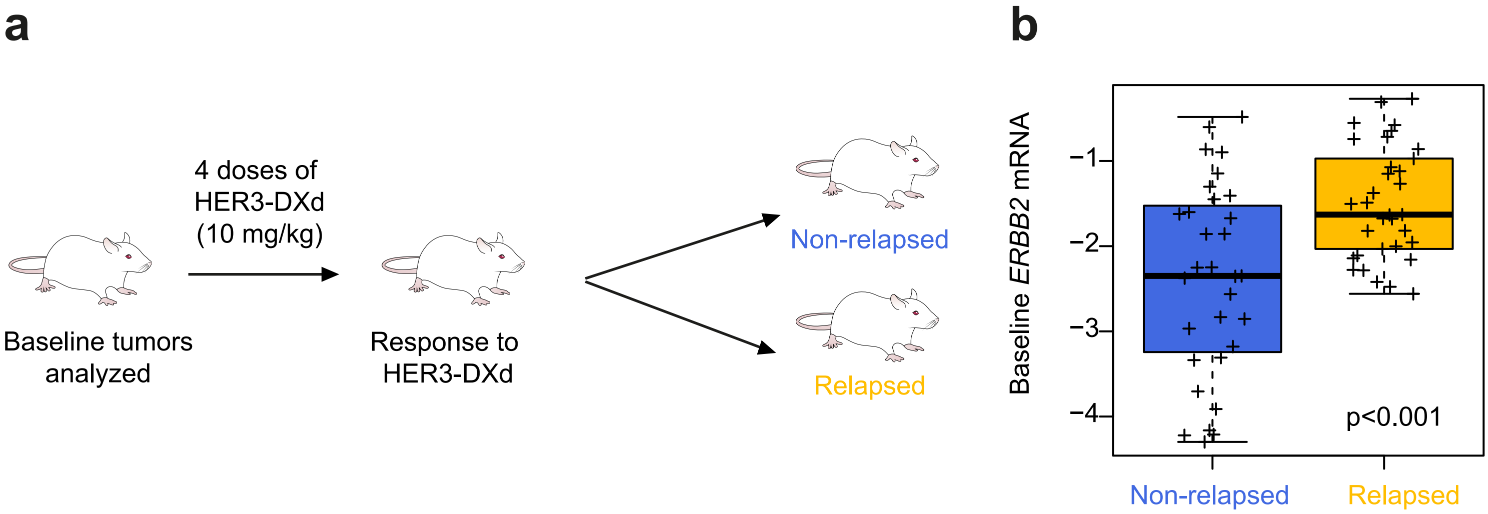

Supplementary Figure 11. Association of pre-treatment baseline *ERBB2* mRNA expression and relapse after HER3-DXd treatment in 30 PDX models (Òdena et al. SABCS 2021) (a) *ERBB2* mRNA expression was assessed in baseline tumors of a cohort of PDX models treated with 4 doses of 10mg/kg of HER3-DXd. PDXs were classified as relapsed and non-relapsed. **(b)** Boxplot of the expression of *ERBB2* mRNA in baseline tumors of PDX that relapsed and did not relapse after HER3-DXd treatment. For the boxplot, center line indicates median; box limits indicate upper and lower quartiles; whiskers indicate 1.5× interquartile range. P-value (p) was determined by two-tailed unpaired t-test. Source data are provided as a Source Data file.

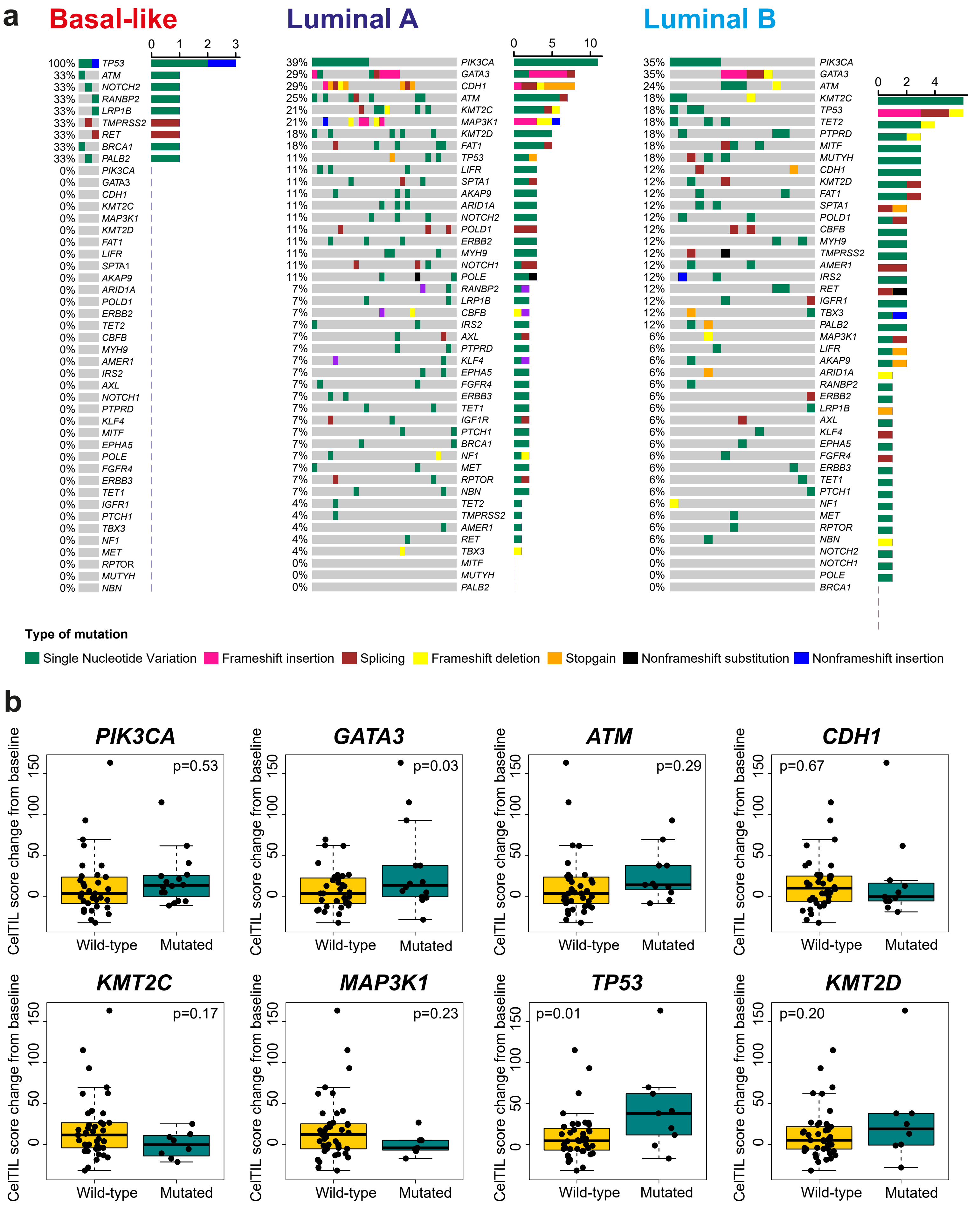

Supplementary Figure 12. *TP53* mutations are associated with with high CelTIL response after one dose of HER3-DXd. (a) OncoPrint representing the frequency of mutations in 49 tumors including 3 basal-like, 28 luminal A and 18 luminal B tumors. **(b)** Boxplot showing CelTIL response after one dose of HER3-DXd in the most frequently mutated genes. For the boxplots, center line indicates median; box limits indicate upper and lower quartiles; whiskers indicate 1.5× interquartile range. P-values (p) were determined by two-tailed unpaired t-tests. Source data are provided as a Source Data file.

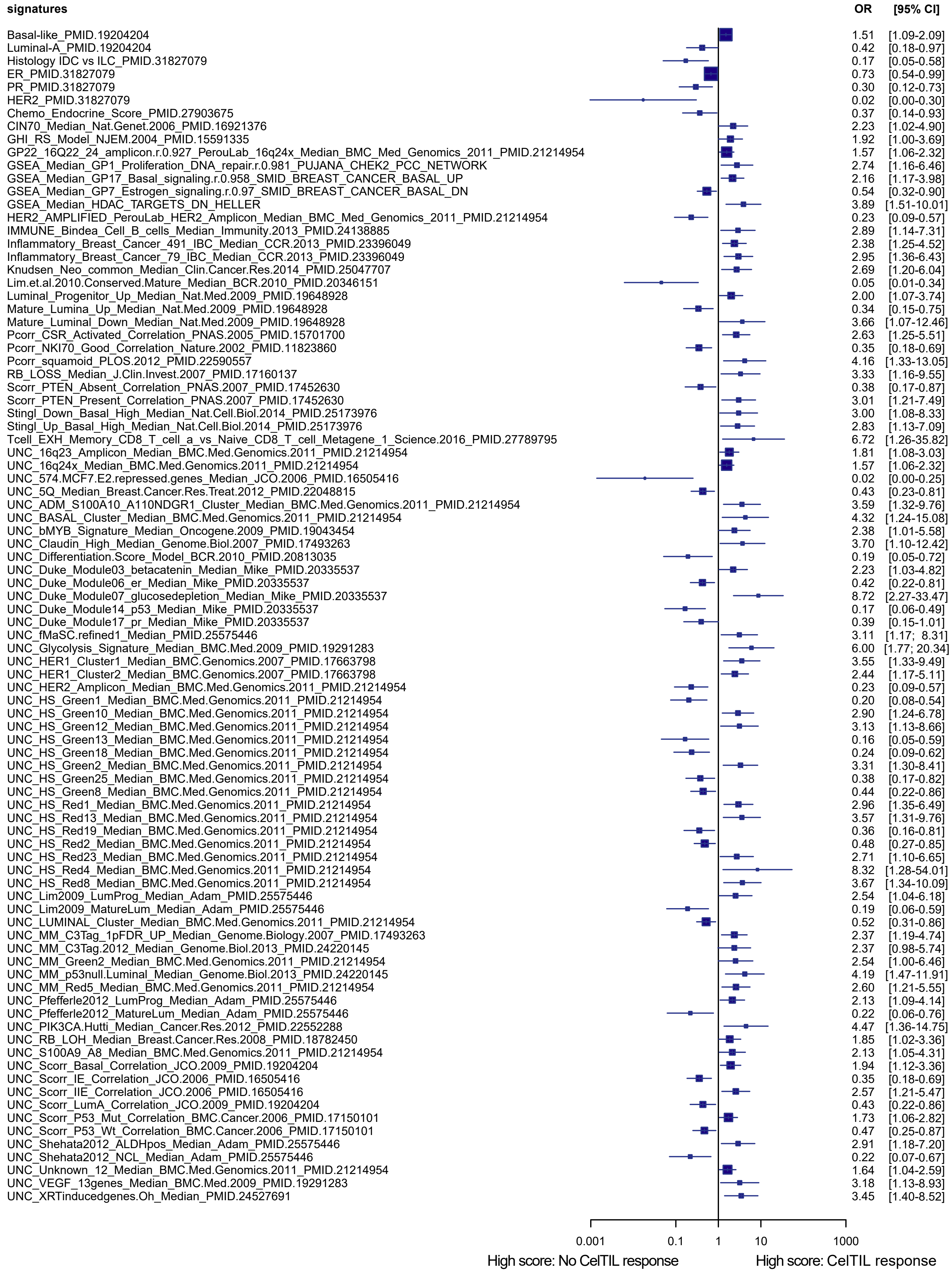

Supplementary Figure 13. Association of CN-based signatures associated with CelTIL response after one dose of HER3-DXd. Forest plot of the 90 CN-based signatures significantly associated with CelTIL response after one dose of HER3-DXd in a logistic regression analysis. Source data are provided as a Source Data file.

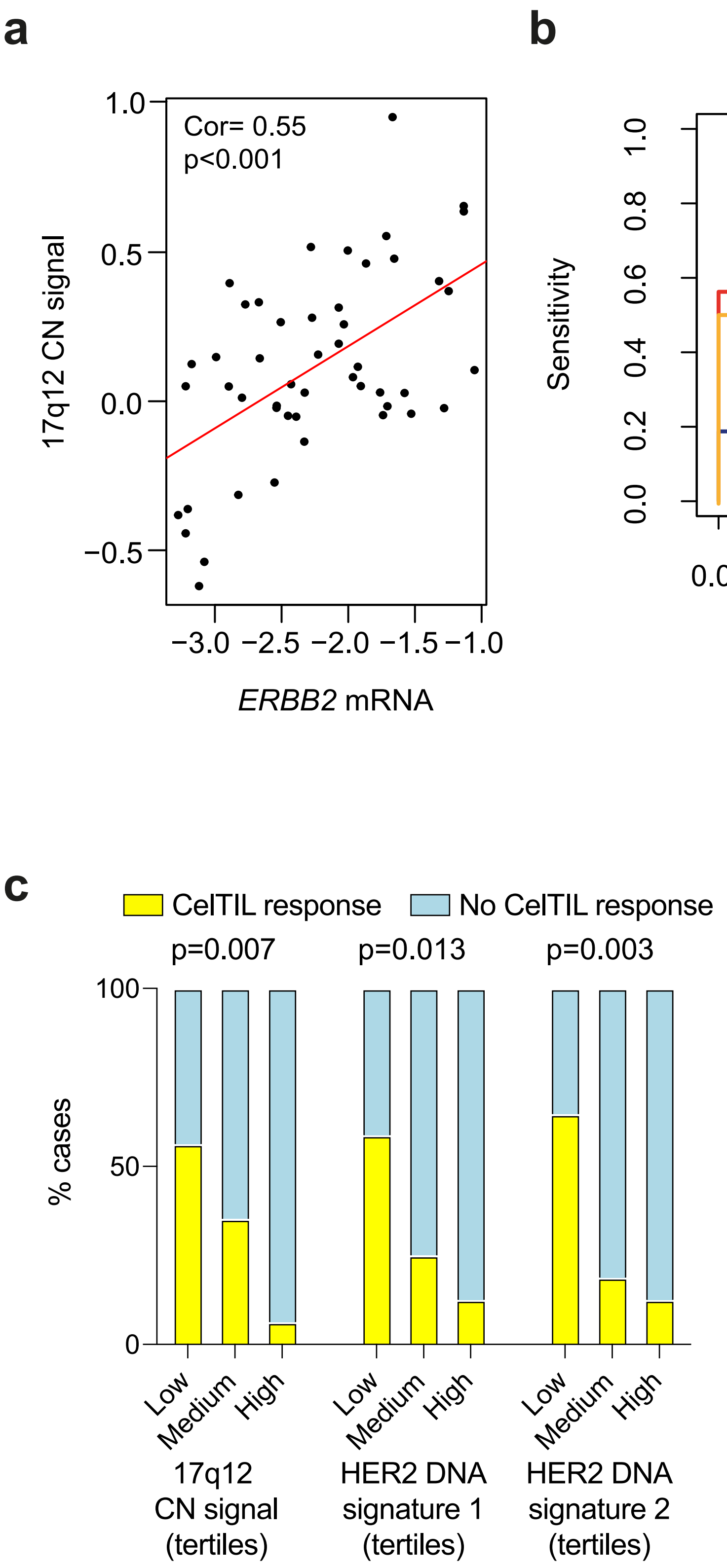

Supplementary Figure 14. Association of the 17q12 CN segment and CN-based HER2-related signatures with CelTIL response after one dose of HER3-DXd. (a) Correlation between *ERBB2* mRNA levels and the signal of the 17q12 CN segment (chr17: 35067383-35272328) in 49 tumors. P-value (p) was determined by Pearson's correlation. **(b)** Performance of the 17q12 CN segment and two CN-based signatures capturing HER2 IHC (HER2 DNA signature 1) and HER2 amplicon (HER2 DNA signature 2) to predict CelTIL response after one dose of HER3-DXd. ROC AUC values are reported. (c) Proportion of tumors CelTIL response after one dose of HER3-DXd in each 17q12 CN segment group (as defined by tertiles: low n=17, medium n=16, high n=16), in the CN-based HER2 signature 1 group (as defined by tertiles: low n=17, medium n=16, high n=16), and in the CN-based HER2 signature 2 group (as defined by tertiles: low n=17, medium n=16, high n=16). P-values (p) were determined by Fisher's exact test. Source data are provided as a Source Data file.

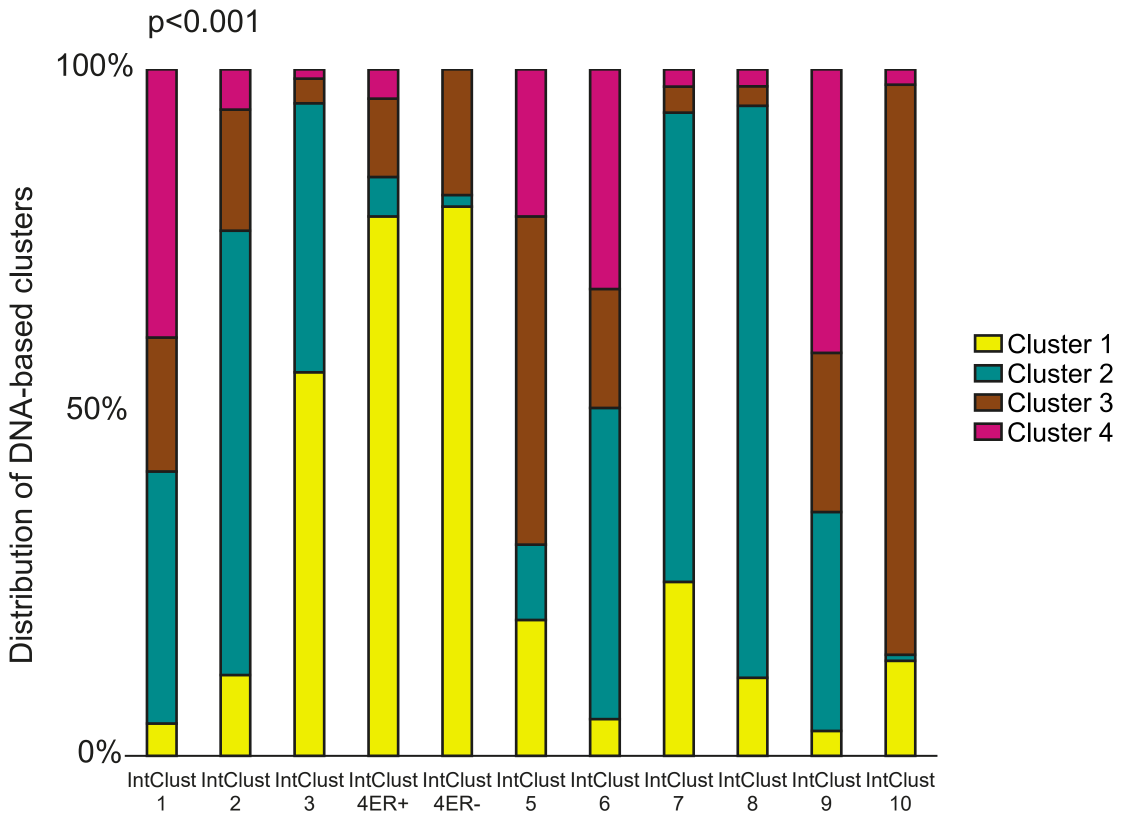

Supplementary Figure 15. Association of DNA-based clusters and integrative clusters in the METABRIC cohort. Distribution of the DNA subtypes (clusters 1-4) and the integrative clusters (IntClust 1-10) of the METABRIC cohort. P-value (p) was determined by Fisher's exact test. Source data are provided as a Source Data file.

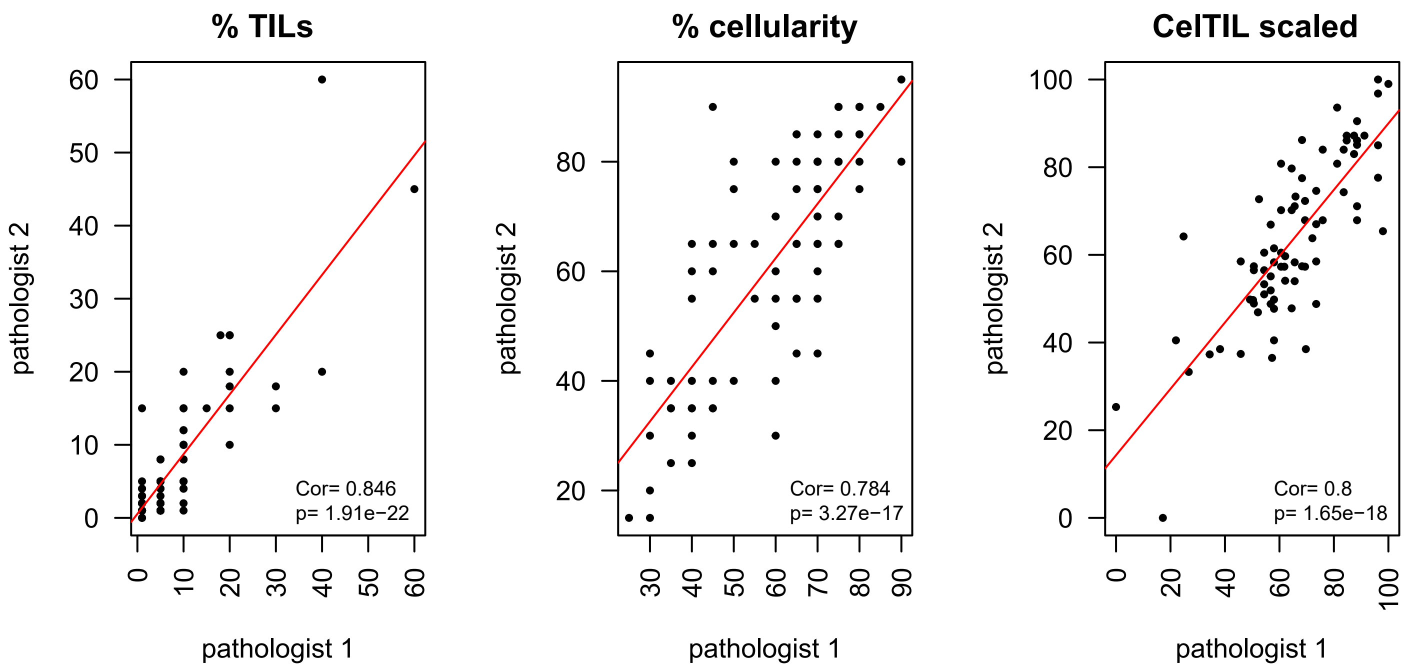

Supplementary Figure 16.Correlations among pathologists. Correlation between two pathologists scoring % TILs, % cellularity and CelTIL of 77 tumors. Source data are provided as a Source Data file.

Supplementary Table 1. Baseline characteristics SOLTI-1805 TOT-HER3 Part B study

	All (n=37)		HR+/HER2- (n=20)		TNBC (n=17)	
	N	%	N	%	N	%
Median age (range)	51 (30-81)		51 (30-65)		50 (30-81)	
Race						
Caucasian	34	92%	17	85%	17	100%
Other	3	8%	3	15%	0	0%
Menopausal status						
Premenopausal	20	54%	12	60%	8	47%
Postmenopausal	17	46%	8	40%	9	53%
Histology		0%		0%		0%
Ductal	33	89%	18	90%	15	88%
Lobular	2	5%	2	10%	0	0%
Other	2	5%	0	0%	2	12%
Tumor stage						
T1	7	19%	5	25%	2	12%
T2	25	68%	13	65%	12	71%
T3	5	14%	2	10%	3	18%
Nodal stage		0%		0%		0%
N0	28	76%	17	85%	11	65%
N1	9	24%	3	15%	6	35%
Grade						
G1	2	5%	2	10%	0	0%
G2	11	30%	10	50%	1	6%
G3	19	51%	6	30%	13	76%
Unknown	5	14%	2	10%	3	18%
BaselineERBB3						
high	2	5%	2	10%	0	0%
medium	11	30%	10	50%	1	6%
low	3	8%	2	10%	1	6%
ultralow	21	57%	6	30%	15	88%
PAM50 subtype						
Basal-like	17	46%	3	15%	14	82%
HER2-enriched	4	11%	1	5%	3	18%
Luminal A	9	24%	9	45%	0	0%
Luminal B	7	19%	7	35%	0	0%

Supplementary Table 2. Grades of treatment-emergent adverse events (TEAEs)

TEAEs	Part B 5.6 mg/kg (n=37)		Part A 6.4 mg/kg (n=78)	
	N	%	N	%
All grades	31	84%	74	95%
Grade 4	0	0%	4	5%
Grade 3	2	5%	10	13%
Grade 2	15	41%	45	58%
Grade 1	31	84%	71	91%
Serious adverse events, all grades	2	5%	4	5%
Grade 3	1	3%	3	4%

Supplementary Table 3. Types of TEAEs

TEAEs	Part B 5.6 mg/kg (n=37)				Part A 6.4 mg/kg (n=78)			
	All grades		Grade 3		All grades		Grade ≥3	
	N	%	N	%	N	%	N	%
Nausea	24	65%	1	3%	52	67%	0	0%
Fatigue	17	46%	0	0%	32	41%	0	0%
Alopecia	10	27%	NA	NA	28	36%	NA	NA
Diarrhea	8	22%	0	0%	19	24%	1	1%
Constipation	5	14%	0	0%	10	13%	0	0%
Headache	5	14%	0	0%	2	3%	0	0%
Transaminitis	5	14%	0	0%	15	19%	2	3%
Vomiting	4	11%	0	0%	20	26%	0	0%
Abdominal pain	3	8%	0	0%	17	22%	0	0%
Anemia	3	8%	0	0%	3	4%	0	0%
Erythema	3	8%	0	0%	3	4%	0	0%
Neutrophil count decrease	0	0%	0	0%	15	19%	6	8%

Supplementary Table 4. Baseline characteristics SOLTI-1805 TOT-HER3 Part A translational study

	**Patients with tumor gene expression data (n=77)**		**Patients with tumor NGS DNAseq data (n=49)**	
	**N**	**%**	**N**	**%**
**Median age (range)**	53 (29-78)		52 (34-78)	
**Menopausal status**				
Premenopausal	43	55.84%	26	53.06%
Postmenopausal	34	44.16%	23	46.94%
**Tumor stage**				
T1	26	33.77%	19	38.78%
T2	41	53.25%	22	44.90%
T3	9	11.69%	7	14.29%
T4	1	1.30%	1	2.04%
**Nodal stage**				
N0	55	71.43%	34	69.39%
N1	18	23.38%	13	26.53%
N2	4	5.19%	2	4.08%
**Grade**				
G1	9	11.69%	6	12.24%
G2	44	57.14%	28	57.14%
G3	14	18.18%	9	18.37%
Unknown	10	12.99%	6	12.24%
**Histology**				
Ductal	58	75.32%	38	77.55%
Lobular	18	23.38%	11	22.45%
Tubular	1	1.30%	0	0.00%
**Mean Ki67 (%) (range)**	27 (5-90)		25.7 (10-70)	
**Baseline *ERBB3***				
high	21	27.27%	14	28.57%
medium	21	27.27%	13	26.53%
low	21	27.27%	15	30.61%
ultralow	14	18.18%	7	14.29%
**PAM50 subtype**				
Basal-like	3	3.90%	3	6.12%
HER2-enriched	2	2.60%	0	0.00%
Luminal A	40	51.95%	28	57.14%
Luminal B	32	41.56%	18	36.73%

Supplementary Note 1. SOLTI-1805 TOT-HER3 protocol.

TOT-HER3

WINDOW PROGRAM

CLINICAL TRIAL PROTOCOL

A window-of-opportunity study of U3-1402, a HER3-targeting antibody-drug conjugate in operable breast cancer according to ERBB3 expression - TOT-HER3 Study

**Sponsor**	SOLTI Breast Cancer Research Group
**Principal investigator**	PPD
**Co-Principal investigator**	
**EUDRACT number**	2019-004964-23
**Protocol code number**	SOLTI-1805
**Version**	Final Version 4.0, 1st of April 2022
**Compound**	U3-1402
**Development phase**	0

Contact data

**Writing Committee**	PPD
**Principal Investigator**	
**Co-Principal Investigator**	
**Medical Monitor**	
**Sponsor details**	
**Chief Scientific Officer**	
**Translational Research**	

Protocol Approval Signatures

Study title:	A window-of-opportunity study of U3-1402, a HER3-targeting antibody-drug conjugate in operable breast cancer according to ERBB3 expression - TOT-HER3 study
Study number:	TOT-HER3 / SOLTI-1805
EudraCT number:	2019-004964-23
Version:	Final Version 4.0

Principal Investigator of the study

PPD

Chief Scientific Officer, SOLTI

PPD

Investigator's protocol acceptance page

principal investigator of the site

Study title:	A window-of-opportunity study of U3-1402, a HER3-targeting antibody-drug conjugate in operable breast cancer according to ERBB3 expression - TOT-HER3 study
Study number:	TOT-HER3 / SOLTI-1805
EudraCT number:	2019-004964-23
Version:	Final Version 4.0

I have read the protocol and agree that it contains all necessary details for conducting this study. I will conduct the study as outlined in the following protocol and in compliance with GCP. I will provide copies of the protocol and all drug information relating to pre-clinical and prior clinical experience furnished to me by the Sponsor, **to all physicians responsible to me who participate in this study. I will discuss this material with them to assure that they are fully informed** regarding the drug and the conduct of the study. I agree to keep records on all patient information (Case Report Forms and patient's Informed Consent statement), drug shipment and return forms, and all other information collected during the study for a period not less than the duration of the period provided by national laws.

Name of the Investigator:

PPD

Index

1. Synopsis 11

2. Background 13

2.1 Current treatment of breast cancer 13
2.2 Window of opportunity trials in the neoadjuvant setting 14
2.3 Biological heterogeneity in breast cancer 15
2.4 HER3 signaling in breast cancer 16
2.4.1 Measuring HER3 in breast cancer 19
2.4.2 Measuring ERBB3 mRNA using the nCounter platform 19
2.4.3 ERBB3 mRNA expression in primary tumor versus metastatic tumor samples 21
2.5 Background on U3-1402 22
2.5.1 Nonclinical studies 22
2.5.2 Clinical studies 23
2.6 Summary of Identified and Potential Risks 27
2.7 Immune gene-signatures in BC: role of tumor infiltrating lymphocytes (TILs) 28
2.8 Tumor cellularity and tumor-infiltrating lymphocytes (CelTIL score) as a biomarker of response 29
2.8.1 An early biological readout of drug activity: tumor cellularity and tumor-infiltrating lymphocytes (CelTIL score) 29
2.8.2 Variability of the CelTIL score according to therapy. 29
2.9 Dose Selection Rationale 31
2.10 Study Rationale 31
3. Study objectives and endpoints 33

3.1 Primary objective and endpoint 33
3.2 Secondary objectives and endpoints 33
3.3 Exploratory objectives and endpoints 35
4. Study design 37

4.1 Study design 37
4.2 Trial diagram 39
5. Study Population 40

5.1 Inclusion criteria 40
5.2 Exclusion criteria 42
6. Study treatment 44

6.1 Treatment schedule and duration 44
6.2 U3-1402 44
CCI [redacted]
6.2.3 Adverse events 45
6.2.4 Toxicity management for AEs associated with U3-1402 45
6.3 Concomitant therapy 49
7. Study Assessments 52

7.1 Table of assessments 52
7.2 Screening 52
7.3 C1D1 53
7.4 C1D3-D7 53
7.5 C1D21 53
7.6 End of Study (EOS) / Safety follow-up (SFV) visit 54
7.7 10-Months Follow-up data collection 54
7.8 Description of Study Assessments 54
7.8.1 Informed Consent 55
7.8.2 Medical History and Demographic data 55
7.8.3 Inclusion/Exclusion criteria 55
7.8.4 Physical examinations 55
7.8.5 Clinical Breast examination 56
7.8.6 Vital signs and weight 56
7.8.7 Tumor assessment: TNM classification 56
7.8.8 Breast and axillar ultrasound. 56
7.8.9 Assessment of cardiac function 56
7.8.10 Biological sample collection. 57
7.8.11 Laboratory tests 57
7.9 Patient, treatment and study discontinuation 58
7.9.1 Patient Discontinuation 58
7.9.2 Study treatment discontinuation 58
7.9.3 Early study discontinuation 59
8. Evaluation criteria 60
8.1 Biological response assessment 60
8.2 Research blood samples 60
9. Translational research 61
9.1 Biological specimens 61
9.1.1 Tumor tissue samples 62
9.1.2 Collection of blood samples 62
9.2 Gene expression signatures 63
9.2.1 The NanoString assay 63
9.2.2 RNA extraction 63
9.2.3 Technical procedures and data analysis 63
9.3 Genetic analysis 64
9.4 Collection of plasma samples 64
9.5 Sample storage and destruction 64
10. Assessment of safety, monitoring and reporting 66
10.1 Monitoring, recording and reporting of adverse events 66
10.1.1 Time Period and Reporting Timelines for AEs/SAEs 66
10.1.2 AE Definition 66
10.1.3 Follow-up of AEs and SAEs 67
10.2 Evaluation of adverse event 67
10.2.1 Seriousness 67
10.2.2 Severity / intensity 68
10.2.3 Causality 69

**10.2.4**	**Duration**	**69**
**10.2.5**	**Action taken**	**69**
**10.2.6**	**Outcome**	**69**
10.3	Abnormal laboratory values	70
10.4	Reporting of adverse events of special interest (Study Specific AESIs)	70
10.5	Pregnancy	70
10.6	Expedited reporting of adverse events to Regulatory Authorities and the Ethics Committee	71
**11.**	**Statistical considerations**	**72**
11.1	Statistical and analytic plan	72
**11.1.1**	**Analysis populations**	**72**
**11.1.2**	**Statistical analysis**	**72**
**11.1.3**	**Planned analysis**	**73**
11.2	Sample size determination	74
**12.**	**Direct access to source data / documents**	**75**
12.1	Study monitoring	75
**12.1.1**	**Responsibilities of the investigators**	**75**
**12.1.2**	**Responsibilities of the sponsor and monitoring**	**76**
**12.1.3**	**Source documents requirements**	**76**
**12.1.4**	**Use and completion of case report forms and additional requests**	**77**
**12.1.5**	**Use of computerized systems**	**77**
12.2	Data Management	78
12.3	Confidentiality	78
**12.3.1**	**Patient records**	**78**
**12.3.2**	**Study documentation and related data**	**78**
**13.**	**Quality control and quality assurance**	**79**
13.1	Protocol deviations	79
13.2	Insurance	79
13.3	Study committees	79
**13.3.1**	**Steering committee (SC)**	**79**
13.4	Premature discontinuation of the study or close-out of a site	80
**14.**	**Ethics**	**81**
14.1	Independent Ethics Committee	81
14.2	Ethical conduct of the study	81
14.3	Informed Consent	82
14.4	Clinical trial / protocol amendments	83
**15.**	**Data handling and record keeping**	**84**
15.1	Subject records and source data	84
15.2	Study documentation and storage of records	84
15.3	Property rights	85
**16.**	**Publication policy**	**87**
**17.**	**References**	**88**
**18.**	**Appendices**	**95**
18.1	Appendix A Table of assessments	96
18.2	Appendix B– Acceptable methods of contraception	99

18.3 Appendix C – ECOG performance status 100
18.4 Appendix D- Adverse Events of Special Interest 101
18.5 Appendix E- Modified Response Evaluation Criteria in Solid Tumors: Assessment of Response of Neoadjuvant Therapy in Early Breast Cancer 102

LIST OF TABLES

Table 1. Distribution of ERBB3 expression across the PAM50 intrinsic subtypes in the METABRIC
[redacted]
Table 5. Summary of the results obtained with absolute changes in CelTIL across 4 SOLTI clinical trials

LIST OF FIGURES

Figure 1. Study design
Figure 2. Distribution of ERBB3-high, ERBB3-medium and ERBB3-low tertiles within the various IHC subtypes in the METABRIC database.
Figure 3. Distribution of tumor samples based on ERBB3 and ERBB2 mRNA levels in the
[redacted]
Figure 5. Expression of ERBB3 across 644 HR+/HER2- tumor samples according to their origin: primary or metastatic.
Figure 6. Tumor Volume of MDA-MB-453 Tumors Xenografted in Nude Mice Administered U3-1402
Figure 7. Study Design of Study U31402-A-J101 (NCT02980341).
Figure 8. Distribution of ERBB3 cohort, ICH expression and PAM50 subtype in the first 30 patients enrolled in TOT-HER3 trial.
Figure 9. CelTIL change by patient (left), indvidual changes in TILs and Celularity (right) along with ERBB3 cohort, clinical response by palpation and intrinsic subtype in the first 30 patients enrolled in TOT-HER3 trial.
Figure 10. Changes of CelTIL and association with response across SOLTI trials.

1. List of abbreviations

AC	Doxorubicin and cyclophosphamide
ADC	antibody-drug conjugate
AE	Adverse event
ALT	Alanine aminotransferase
ANC	Absolute neutrophil count
AP	Alkaline phosphatase
aPTT	Activated partial thromboplastin time
ASCO-CAP	American Society of Clinical Oncology-College of
AST	Aspartate aminotransferase
BC	Breast cancer
BCRP	Breast Cancer Resistance Protein
BUN	Blood urea nitrogen
CI	Confidence interval
CIS	Carcinoma in situ
Cm	Centimeter
CR	Complete response
CRF	Case report form
CSR	Clinical study report
CT	Computerized tomography
CTCAE	Common terminology for classification of adverse events
ctDNA	Circulating tumor DNA
dL	Deciliter
DSUR	Development safety updated report
ECG	Electrocardiogram
ECHO	Echocardiography
ECOG	Eastern Cooperative Oncology Group
e-CRF	Electronic clinical report form
EGFR	epidermal growth factor receptor
EOS	End of Study
ER	Estrogen receptor
ERBB3	Receptor tyrosine-protein kinase erbB-3
FEC	5-Fluorouracil, epirubicin, and cyclophosphamide
FFPE	Formalin–fixed paraffin–embedded
FISH	Fluorescence in situ hybridization
FNA	fine needle aspiration
GCP	Good clinical practice
HER2	Human epidermal growth factor receptor 2
HER3	Human epidermal growth factor receptor 3
HIV	Human immunodeficiency virus
HR	Hazard ratio
ICF	Informed consent Form
IC50	Half maximal inhibitory concentration
IHC	Immunohistochemistry
ICH	International Conference on Harmonisation of Technical Requirements
IEC	Independent Ethics Committee
IHC	Immunohistochemistry
IMP	Investigational Medicinal Product
INR	International normalized ratio
IRB	Institutional Review Board
ITT	Intent-to-treat

IV	Intravenous
Kg	Kilogram
L	Liter
LDH	Lactate dehydrogenase
m2	Square meter
mAb	Monoclonal antibody
MedDRA	Medical dictionary for regulatory activities
mg	Milligram
mL	Milliliter
mm	Millimeter
mmHg	Millimeters of mercury
MRI	Magnetic resonance imaging
mRNA	Messenger ribonucleic acid
MUGA	Multiple Gate Acquisition
NAC	Neoadjuvant Chemotherapy
NaCl	Sodium chloride
NCI	National Cancer Institute
nM	Nanomolar
NRG	Neuroregulin
NSABP	National Surgical Adjuvant Breast and Bowel Project
OATP	Organic anion-transporting protein
OR	Odds ratio
PD	Progressive disease
PD-1	Programmed death 1
PD-L1	Programmed death-ligand 1
PFS	Progression-free survival
Pgp	P-glycoprotein
PgR	Progesterone receptor
PI3K	phosphatidylinositol-3-kinase
PK	Pharmacokinetics
PTT	Partial thromboplastin time
RTK	Receptor tirosin kinase
SAE	Serious adverse event
SC	Steering Committee
SFV	Safety Follow up visit
SOLTI	Spanish Breast Cancer Research Group
SP	Safety population
sTILs	Stromal tumor infiltrated lymphocytes
SUSAR	Suspected unexpected serious adverse reaction
TEAE	Treatment emergent adverse event
TILs	Tumor infiltrated lymphocytes
TMB	Tumor mutation burden
TNBC	Triple-negative breast cancer
ULN	Upper level of normal
US	Ultrasound
µL	Microliter

2. Synopsis

**Title**	A window-of-opportunity study of U3-1402, a HER3-targeting antibody-drug conjugate in operable breast cancer according to ERBB3 expression - TOT-HER3 study	
**Protocol Code Number / EudraCT Number**	SOLTI-1805 / 2019-004964-23	
**Principal investigator**	PPD	
**Co-principal investigator**		
**Chief Scientific Officer**		
**Sponsor**	SOLTI Breast Cancer Research Group	
**Indication**	Treatment naïve early breast cancer.	
**Experimental treatment**	• Part A: U3-1402 monotherapy at a dose of 6.4 mg/kg will be administered once by intravenous (IV) infusion<br>• Part B: U3-1402 monotherapy at a dose of 5.6 mg/kg will be administered once by intravenous (IV) infusion	
**Type**	Phase 0. This is a prospective, multicenter, single arm, window-of-opportunity study.	
**Primary Objectives**	**Primary objective**	**Primary endpoint**
	To evaluate changes in CelTIL score after one dose of 6.4 mg/kg of U3-1402 in patients with HR+/HER2-negative early breast cancer group.	Mean change in CelTIL score per central assessment between paired samples (baseline and C1D21)
**Trial design**	This is a prospective, multicenter, single-arm, window-of-opportunity study evaluating the biological effect of U3-1402 in treatment naïve patients with early breast cancer, whose primary tumors are ≥1 cm by ultrasound evaluation.<br><br>**Figure 1. Study design.**<br>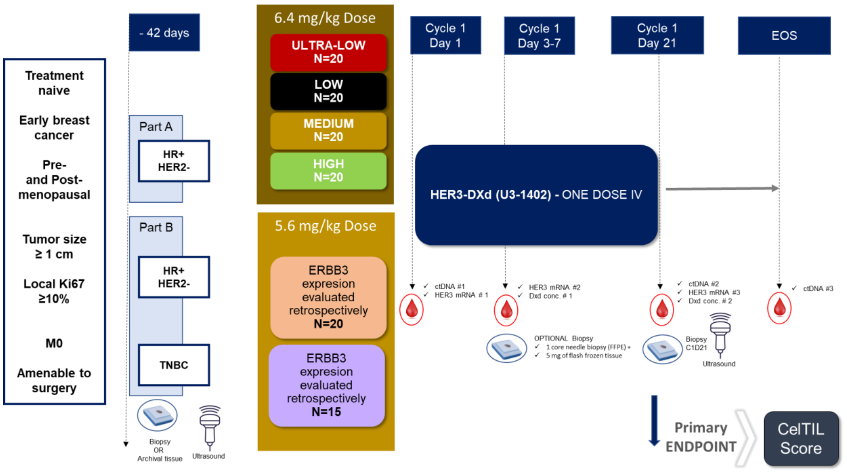	

**Length of study**	Planned study period (LPO): 24 months (from FPI)<br>Recruitment period: Part A 16 months, Part B - 10 months<br>Treatment period of 3 weeks.<br>Follow-up: 1 additional month. Soft Follow-up: 10 months<br>Participating sites: 10 sites
**Planned sample size**	In Part A, a target sample size of 80 patients with HR+/HER2-negative disease will be included. To ensure the representation of the entire range of ERBB3 expression, ERBB3 expression will be determined prospectively (≤7 working days) and approximately 20 patients will be included in one of each of the 4 groups based on ERBB3 quartile expression (ultra-low, low, medium and high).<br><br>Part B of the study will target to include 20 patients with HR+/HER2-negative breast cancer and 15 patients with TNBC to be treated with a dose of 5.6 mg/kg. No formal sample size calculation has been made, but it will maintain a reasonable proportion with Part A, also allowing recruitment within a reasonable time frame. ERBB3 expression will be determined retrospectively.
**Statistical methodology**	The main objective of this proof of concept study is to estimate the biological effect of a single dose of U3-1402 treatment in a window opportunity study before surgical procedure in a patient with treatment naïve breast cancer. The study is designed to formally assess the overall mean change in CelTIL score between baseline and C1D21 after one dose of U3-1402 (part A). However, no formal comparison between ERBB3 cohorts will be done and statistical analyses will be performed for estimations of proportions or means (or medians) for all variables by confidence intervals calculations. A specific statistical analysis plan (SAP) will be performed before the formal close of the database.

3. Background

3.1 Current treatment of breast cancer

Breast cancer is a life-threatening disease and is the most diagnosed cancer among women worldwide, accounting for 1 in 4 cancer cases. It is the most frequent cancer and is the leading cause of death from cancer in women. The estimated 2.3 million new cases indicate that one in every 8 cancers diagnosed in 2020 is breast cancer. In 2020, there were an estimated 684,996 deaths from breast cancer, with a disproportionate number of these deaths occurring in low-resource settings. Although, the death rate from all cancers in the United States has continued to decline, from 1991 to 2018, the cancer death rate has fallen 31%. This includes a 2.4% decline from 2017 to 2018 – a new record for the largest one-year drop in the cancer death rate[1]. In 2021, roughly 1.9 million people will be diagnosed with cancer in the United States. An estimated 281,550 women and 2,650 men will be diagnosed with breast cancer, which makes it the most common cancer diagnosis[2]

Although metastatic breast cancer represents ~5% of cases at diagnosis, nearly one third of breast cancer patients with non-metastatic disease will eventually develop metastases during follow-up[4].

Breast cancer is considered a biologically heterogeneous disease. Endocrine therapies that target estrogen receptor (ER) signaling pathways for ER-positive disease and human epidermal growth factor receptor 2 (HER2)-targeted therapies for HER2-positive disease play a critical role in the treatment of most patients with breast cancer[5–8] . However, not every breast tumor responds equally to these agents.

Hormone receptor (HR)-positive and HER2-negative breast cancer accounts for approximately 60-70% of all diagnoses. For this type of breast cancer, all patients are recommended adjuvant endocrine therapy for 5-10 years. In addition, in cases with high risk of relapse, adjuvant chemotherapy containing anthracycline and taxane-based combinations is also recommended[9]. However, despite current adjuvant therapies, high-risk patients still have a substantial risk of relapsing as shown by the large adjuvant studies GEICAM/9906 and CALGB/9741[10–12]. When the HR-positive/HER2-negative disease is locally recurrent/metastatic, the median overall survival does not exceed the 30-35 months[13–15]. There are several mechanisms that can lead to primary and/or secondary hormonal resistance in HR+ breast cancer: decrease of ER expression, loss of ER expression, or upregulation of growth factor signaling pathways, such as the epidermal growth factor receptor (EGFR)/HER2, the mitogen-activated protein kinase (MAPK), or the PI3K/AKT/mTOR pathways[16].

Therefore, new therapies and treatment strategies to improve survival outcome of patients with HR-positive/HER2-negative breast cancer are of great importance.

Triple-negative breast cancer (TNBC), defined by the lack of expression of HER2 and HR, represents about 15% of invasive breast carcinomas[17,18]. It has an unfavorable prognosis, with an aggressive pattern of progression and a high rate of early-occurring metastasis. TNBC does not

respond to endocrine therapy. The lack of targeted therapy for TNBC makes it one of the most challenging subtypes of invasive breast cancer to treat today. Despite its initial response to chemotherapy, many patients with TNBC relapse quickly, either locoregionally or with the development of visceral metastasis, including lung, liver and brain metastasis. Recent evidence shows that TNBC also comprises a biologically heterogeneous population[17], with wide differences in response to therapy. It is therefore an important unmet medical need and new therapies are required.

3.2 Window of opportunity trials in the neoadjuvant setting

Attempting to conduct translational research to identify predictive biomarkers is challenging and inefficient when analyzing heavily pre-treated tumors with high degrees of heterogeneity. In order to increase the success rate of drugs in later stage clinical trials, accurate and efficient methods of understanding biology, pharmacodynamics and identifying predictive biomarkers is paramount.

Window of opportunity (WOO) studies, in which treatment-naïve patients are briefly exposed to therapy to observe tumor biology, are emerging as a strategy to identify biomarkers[23].

Those studies, also called phase 0, can take a variety of inter-related forms, all making use of the small gaps in standard cancer therapy[24].

In the neoadjuvant setting, WOO studies allow patients to receive one or more new compounds between their cancer diagnosis and standard treatment (mainly surgery); and tumor biopsies before and after the investigational treatment are collected for translational research, assessing cellular and molecular changes, which can help in the identification of undiscovered patterns of molecular determinants of drug sensitivity or resistance[25] . This strategy offers several advantages, particularly in the age of precision medicine.

First, it allows evaluation of changes in a known target following exposure to targeted therapy in vivo.

Secondly, molecular analysis testing can be done on treatment-naïve patients that are less likely to harbor resistance mutations, or molecular alterations that occur as a result of prior treatment exposure. For example, in BC a novel study drug can be given to patients between the diagnostic breast biopsy and the planned surgical resection. It is hoped that this type of study can improve understanding of an agent’s biologic effect and potential target population early in its development without disturbing the standard treatment pattern of a patient. Trials of this type provide tumor tissue before and after the administration of a new therapy (in chemo- and radiotherapy naïve patients) for biomarker analyses of potential mechanisms of response/resistance.

Thirdly, it allows the evaluation of treatments in combination with, or instead of, standard of care therapy that would not be possible in traditional clinical trials.

In conclusion, WOO trials represent an exciting opportunity to learn about mechanisms of action of novel therapies, molecular activity, and efficacy. They also provide an opportunity for biomarker evaluation to appropriately enrich study population for later stage clinical trials.

Considering this clinical trial design, we have launched a Window program at SOLTI that offers a clinical platform for efficient testing of anticancer agents and combinations in breast cancer. Consisting of a small sample size, there is a rapid read-out of antitumor activity by means of biological endpoints. Together with a comprehensive analysis and biomarker identification, these studies grant a smarter design of phase II/III strategy.

3.3 Biological heterogeneity in breast cancer

Breast cancer is a clinically and biologically heterogeneous disease. In terms of global gene expression, four main molecular subtypes (Luminal A, Luminal B, HER2-enriched [HER2-E] and Basal-like) have been identified and intensively studied in the last 15 years[17,26–28]. Known as the 'intrinsic subtypes of breast cancer', these groups of tumors have revealed critical differences in incidence[29,30], survival[19,31,32], and response to treatment[20,33]. Importantly, the information provided by the intrinsic subtypes complements and expands the information provided by classical clinical parameters (e.g. age, node status, tumor size, histological grade) and pathological markers (estrogen receptor [ER], progesterone receptor [PR] and HER2)[10,32,34], all of which are routinely used today in the clinic to stratify patients for prognostic predictions and to select treatments.

The importance of intrinsic subtyping in breast cancer has been highlighted in one of the most complete molecular characterization studies that have ever been performed in breast cancer[26]. In this study, led by The Cancer Genome Atlas Project (TCGA), more than 500 primary breast cancer were extensively profiled at the DNA (i.e. methylation, chromosomal copy-number changes and somatic and germline mutations), RNA (i.e. miRNA and mRNA expression) and protein (i.e. protein and phosphor-protein expression) levels using the most recent technologies[26]. In a particular analysis of over 300 primary tumors (i.e. shown in Figure 2 of that publication https://www.nature.com/articles/nature1141221), 5 different data-types (i.e. all except DNA mutations) were combined together in a cluster of clusters in order to identify how many biological homogenous groups of tumors one can identify in breast cancer. The consensus clustering results showed the presence of 4 main entities of breast cancer but, more importantly, these 4 entities were found to be very well recapitulated by the 4 main intrinsic subtypes (Luminal A, Luminal B, HER2-E and Basal-like) as defined by mRNA expression only[35]. Overall, these results suggest that intrinsic subtyping captures most of the biological diversity occurring in breast cancer.

In 2009, a clinically applicable gene expression-based predictor that robustly identifies these main intrinsic subtypes by quantitative measurement of 50 genes, called the PAM50 subtype predictor[36]. PAM50 assay and the three-gene model for identifying the major and clinically relevant molecular subtypes of breast cancer were described. This analysis is performed by measuring the expression of 50 genes with 3 different platforms (microarrays, RT-qPCR and nCounter) and 2 types of RNA (from formalin-fixed paraffin embedded blocks (FFPB) and fresh

frozen tumor tissue). As well as identifying a subtype, the PAM50 predictor also offers a risk of relapse (ROR) score. The information provided by those intrinsic subtypes complements and expands the information provided by classical clinical parameters (e.g. age, node status, tumor size, histological grade) and pathological markers (ER, PR and HER2)[10,31,32] 14,19,20 all of which are routinely used today in the clinic to stratify patients for prognosis and treatment selection.

As mentioned above not all the intrinsic subtypes responds equally to a specific agent. Thus, a major challenge in breast cancer management is how to prospectively select patients who will derive the maximum benefit from a given drug and minimizing unnecessary toxicities for patients with non-responsive disease.

3.4 HER3 signaling in breast cancer

The HER family of tyrosine kinase receptors HER1 (ErbB1/ epidermal growth factor receptor), HER2 (ErbB2/neu), HER3 (ErbB3), and HER4 (ErbB4) initiate a complex signal transduction cascade modulating cell proliferation, survival, adhesion, migration, and differentiation. Growth factor–induced HER signaling is essential for normal cellular processes and plays a key role in the aberrant development and growth of tumor cells. Each of the HER ligands promotes different homodimerization and heterodimerization patterns within the HER family.

HER2 enhances and stabilizes dimerization but has no ligand, whereas HER3 has no inherent kinase activity and requires dimerization with another HER family members (HER1, HER2, HER4) to promote downstream signaling[37]. In addition, HER3 is described to signal in complexes with several other receptor tyrosine kinases (RTKs) such as fibroblast growth factor receptor 2 (FGFR2) and hepatocyte growth factor receptor (HGFR)[38]. Neuregulin-1 and Neuregulin-2 are high affinity ligands for HER3.

Heregulins (HRGs; aliases: neuregulin, Neu differentiation factor, glial growth factor, acetylcholine receptor-inducing activity) are growth factors that trigger multi-step kinase-dependent signaling events after binding to the transmembrane receptors HER3. Specifically, in cancer, HRGs/HER3 are implicated in stemness, invasiveness, proliferation, resistance to apoptosis, and angiogenesis[39,40].

Numerous studies observe that HER3 activation play a critical role in cancer development, associating it with poor clinical outcomes and as a major cause of treatment failure in cancer therapy:

- HER3 mutations are common in lobular invasive breast cancer[41]. In a study of initial 630 invasive lobular breast cancer primary tumors to characterize the mutational landscape, HER2 and HER3 were mutated in 5.1% and 3.6% of the tumors, with most of these mutations having a proven role in activating the human epidermal growth factor

receptor/ERBB pathway. HER2 and AKT1 mutations were associated with an increased risk of early relapse. HER2, HER3, and AKT1 mutations represent high-prevalence targets for therapy and are necessary to individualize their treatment.

CCI [redacted]

- Several studies indicate that activation of HER3 signaling is one major cause of treatment failure to EGFR or anti-estrogen- based therapies[44,45].
- HER3 overexpression predicts resistance to trastuzumab, though HER3 expression has not been shown as a predictive factor in combination treatment of trastuzumab and pertuzumab in HER2-positive breast cancers[46]. Genetic and functional studies on trastuzumab indicate that activation of PI3K/AKT and SRC signaling are major determinants of trastuzumab-induced resistance[47,48].
- HER3 overexpression also correlates with resistance to lapatinib[49,50].
- Enhanced HER3 expression helps bypass responsiveness to endocrine therapies[51–54].
- Co-expressors of HER2/HER3 more likely to relapse on tamoxifen[51–54].
- HER3 attenuation abrogates HER2-mediated tamoxifen resistance[51–54]. In addition, another study demonstrates that fulvestrant resistant MCF-7 cells depend on increased HER3 and NRG-2 expression to maintain their growth and survival[55]. Therefore, HER3 might serve as a crucial biomarker in estrogen withdrawal therapy in luminal breast cancers and in regulating ER-mediated pathways.
- HER3 is overexpressed in approximately 30-50% of breast cancers and confers a worse prognosis[56–59]. HER3 is emerging as an important molecule in ER+ breast cancers. HER3 mRNA is highest in ER+ or luminal tumors[60,61], consistent with the observation that HER3 is required for cell survival in the luminal but not the basal normal mammary epithelium[62]. Its expression varies in different IHC-subgroups or different PAM50 intrinsic subtypes. (**Figure. 2 and Table 1**).
- The expression of ERBB3 varies in a wide range in different tumors of breast cancer (**Figure. 3**)

Figure 2. Distribution of ERBB3-high, ERBB3-medium and ERBB3-low tertiles within the various IHC subtypes in the METABRIC database.

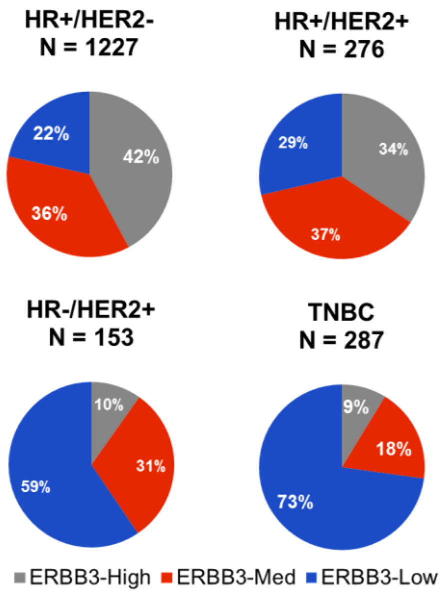

Table 1. Distribution of ERBB3 expression across the PAM50 intrinsic subtypes in the METABRIC database.

	ERBB3-High	ERBB3-Med	ERBB3-Low
**Luminal A**	**47.4%**	**38.8%**	**13.8%**
**Luminal B**	**48.3%**	**34.9%**	**16.9%**
**HER2-E**	**32.8%**	**37.9%**	**29.3%**
**Basal-like**	**2.0%**	**20.0%**	**78.0%**

Figure 3. Distribution of tumor samples based on ERBB3 and ERBB2 mRNA levels in the METABRIC. The green lines identify tertiles of ERBB3 expression.

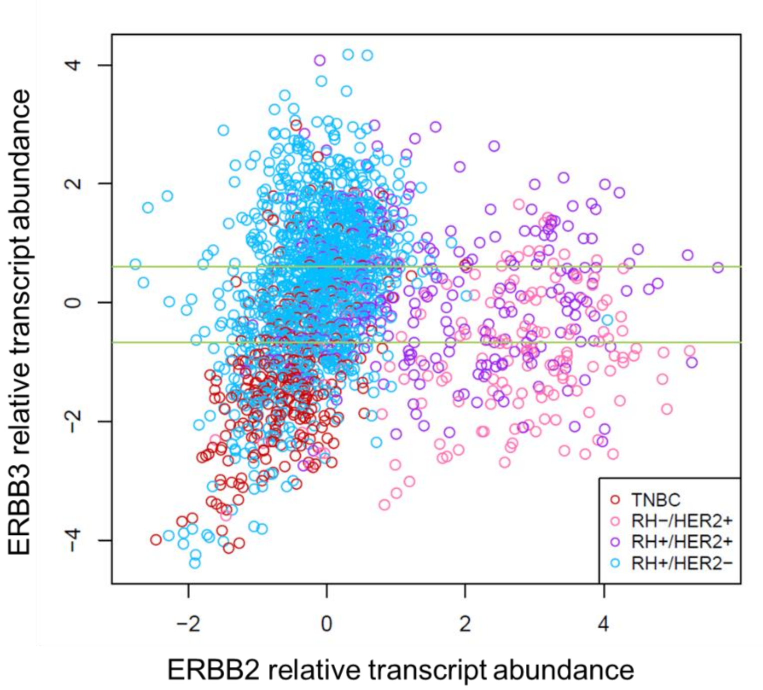

3.4.1 Measuring HER3 in breast cancer

Important technical limitations exist with immunohistochemistry-based assays, such as different sensitivities of the antibodies used and its subjectivity in scoring and cut-off determination. Therefore, clinical implementation of robust and reproducible genomic assays using platforms such as the nCounter is needed. High reproducibility of different biomarkers measurement has been reported using the nCounter platform starting from either already purified RNA or from tissue[63–66]. In addition, a large dynamic range (~116-fold) of ERBB3 mRNA expression is identified in the METABRIC breast cancer dataset (**Figure. 3**.)

3.4.2 Measuring ERBB3 mRNA using the nCounter platform

To date, we have analyzed ERBB3 mRNA using the Nanostring nCounter platform in 1,600 tumor samples using formalin-fixed paraffin-embedded tumor samples. Among the 1,580 samples with IHC data, 65.4% are HR+ and 18.2% are HER2+. IHC subtype distribution is as follows: 1) 51.9% HR+/HER2-negative, 2) 29.9% triple-negative, 3) 13.5% HR+/HER2+ and 4) 4.7% HR-/HER2+. In this dataset, the range of ERBB3 mRNA expression has an 18.6-fold difference in gene expression (i.e. from the lowest to the highest ERBB3 value) and the inter-quartile range (which is the difference between the 75th and 25th percentiles) is 1.5 (in log base 2), which equals to a difference in expression of 2.9-fold (**Figure. 4)**. Using quartiles, **Table 2** shows the proportion of tumors within each quartile based on their IHC subtype. Interestingly, these proportions are very similar as the

proportions found in the METABRIC dataset, which used fresh-frozen tissues and microarray (**Table 3**).

Figure 4. Expression of ERBB3 across 1,600 breast tumor samples (in-house dataset).

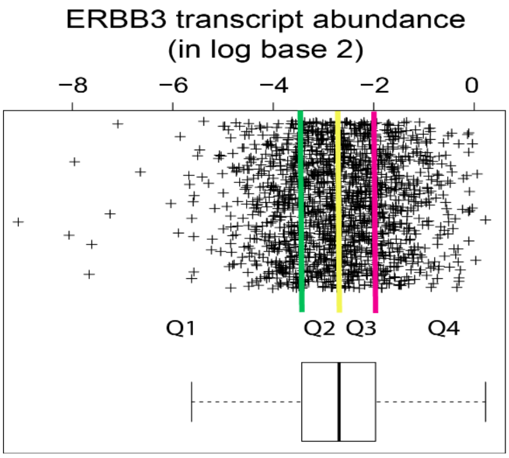

Table 2. Number and proportion of tumor samples within each quartile (Q1-4) based on their IHC subtype.

	FirstQuartile	%	SecondQuartile	%	ThirdQuartile	%	FourthQuartile	%	Total	%
RH-/HER2+	21	28.4%	23	31.1%	22	29.7%	8	10.8%	74	100%
RH+/HER2-	95	11.6%	183	22.3%	242	29.5%	300	36.6%	820	100%
RH+/HER2+	45	21.1%	55	25.8%	53	24.9%	60	28.2%	213	100%
TNBC	229	48.4%	136	28.8%	83	17.5%	25	5.3%	473	100%

Table 3. Comparison of the distribution of tumor samples according to ERBB3 mRNA expression in METABRIC dataset versus our in-house nCounter-based dataset. Proportions of tumor samples within each quartile based on IHC subtype and correlation coefficients between both datasets.

METABRIC	FirstQuartile	SecondQuartile	ThirdQuartile	FourthQuartile
RH-/HER2+	46.4	28.8	21.6	3.3
RH+/HER2-	14.3	24.4	28.9	32.4
RH+/HER2+	17.0	31.2	25.7	26.1
TNBC	65.2	19.5	9.8	5.6

In-house	FirstQuartile	SecondQuartile	ThirdQuartile	FourthQuartile
RH-/HER2+	28.4	31.1	29.7	10.8
RH+/HER2-	11.6	22.3	29.5	36.6
RH+/HER2+	21.1	25.8	24.9	28.2
TNBC	48.4	28.8	17.5	5.3

	Corr.Coefficient
RH-/HER2+	0.76
RH+/HER2-	0.99
RH+/HER2+	0.76
TNBC	0.94

3.4.3 ERBB3 mRNA expression in primary tumor versus metastatic tumor samples

In Cejalvo and colleagues' article[67], we reported the results of gene expression data in 123 paired tumor samples (primary versus metastatic) representing all IHC subtypes. We evaluated the expression of 105 breast cancer-related genes using the nCounter platform. ERBB3 was included in the gene list. As reported, ERBB3 was not found within the list of genes differentially expressed between both tumor samples.

Further, we studied the ERBB3 expression of 644 HR+/HER2- tumors of patients recruited in the EGF3008 trial, a study of first-line metastatic disease[68]. The expression of ERBB3 did not differentiate between primary and metastatic tumor samples. As a summary, we report here the density plots of ERBB3 expression in this dataset (**Figure. 5)**.

Figure 5. Expression of ERBB3 across 644 HR+/HER2- tumor samples according to their origin: primary or metastatic.

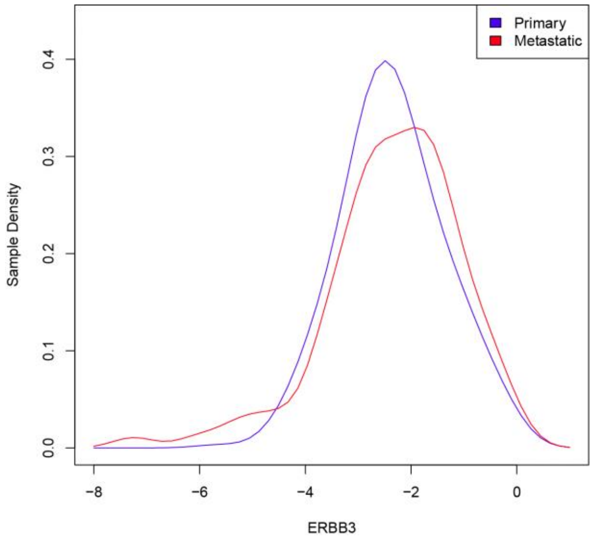

CCI

[redacted]

CCI [redacted]

3.5.2 Clinical studies

Currently, a Phase 1 studies of U3-1402 in subjects with breast cancer is ongoing, study U31402-A-J101 (NCT02980341), safety data of this study is presented below. Adittionally, data from study U31402-A-U102 (NCT03260491) in subjects with NSCLC has been published,

Moreover, a pivotal, phase II, global study in patients with EGFR-mutated NSCLC after disease progression on EGFR TKI and platinum-based chemotherapy has been initiated (HERTHENA-Lung01; NCT04619004). Simirarly, a phase I, dose escalation and dose expansion study to evaluate U3-1402 in combination with osimertinib (and also as monotherapy) in patients with EGFR-mutated NSCLC with tumor progression after treatment with osimertinib monotherapy and no platinum-based chemotherapy (NCT04676477). Another study in metastatic colorectal cancer (NCT04479436).

During the course of this study two interim analysis have been performed, the latest published in SABCS 201 icluding data from 30 patients. Those results are also sumarized below.

3.5.2.1 Study U31402-A-J101

An ongoing, phase 1/2 study[69] (ClinicalTrials.gov NCT02980341) was initiated in 2016 to evaluate the safety, tolerability, and efficacy of U3-1402 in HER3-overexpressing metastatic breast cancer. Eligible patients had HER3-positive (central immunohistochemistry 2+/3+), advanced/unresectable, or metastatic breast cancer that was refractory/intolerable to standard treatment, or for which no standard treatment is available, and an ECOG performance status of 0 or 1. The study includes 3 parts: a Dose Escalation Part to identify the maximum tolerated dose (MTD) of U3-1402; a Dose Finding Part to determine the recommended doses for expansion (RDEs) of U3-1402 and to assess multiple drug administration schedules, including up-titration of

dose, to determine the safety of alternative dosing schedules; and a Dose Expansion Part to confirm the safety and efficacy of U3-1402 at the RDEs for subjects with HER3-positive metastatic breast cancer. (**Figure. 7**).

Figure 7. Study Design of Study U31402-A-J101 (NCT02980341).

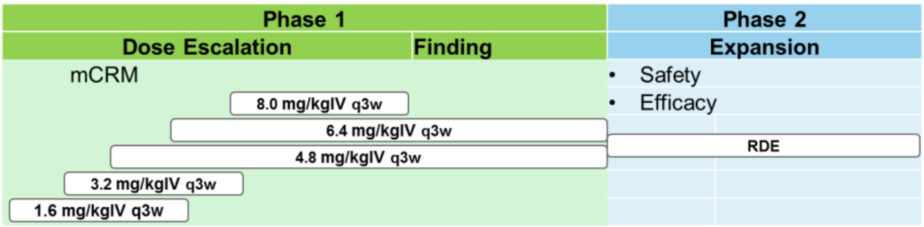

mCRM, modified continuous reassessment method; IV, intravenous; RDE, recommended dose(s) for expansion; q3w, once every 3 weeks.

In the dose-escalation stage of the study, the maximum tolerable dose (MTD) has not been reached and treatment with the U3-1402 was associated with an acceptable safety profile[70].

An analysis of efficacy was performed with a data cut-off date of 18 Aug 2020, and it included 182 subjects with previously treated metastatic breast cancer who received at least 1 dose of study drug. Among these subjects, 116 subjects were in the Dose Expansion Part for HR-positive and HER2-negative metastatic breast cancer and TNBC. The HR-positive cohort was further subdivided into HER3-high (n = 64) and HER3-low (n = 21) cohorts. Subjects in the HR-positive HER3-high cohort were randomized to receive either 4.8 mg/kg (n= 33) or 6.4 mg/kg (n = 31) dose of patritumab deruxtecan q3w, and all other cohorts, including HER3-low (n = 21) and TNBC (n = 31), were treated at 6.4 mg/kg dose only. All results were as per blinded independent central review with confirmation of responses (CR + PR). Preliminary results from expansion cohorts showed a confirmed response rate of 30.3% in the 4.8 mg/kg dose HR-positive/ HER2-negative HER3-high cohort, with a median duration of response (DoR) of 5.0 months (95% CI: 2.8, NE) after a median follow-up of 16.8 months. The confirmed ORR was 12.9% in the HR-positive/HER2-negative HER3-high 6.4 mg/kg cohort, with a DoR of 7.2 months (95% CI: 3.6, 29.8 months) after a median follow-up of 20.4 months. ORR in HR-positive/ HER2-negative HER3-low cohort was 33.3% with median DoR of 5.3 months (95% CI: 3.0, NE) after a median follow-up of 18.7 months. The HER3-high TNBC 6.4 m/kg cohort showed a preliminary ORR of 16.1% with a DoR not reached (95% CI: 4.2, NE) after a median follow-up of 7.4 months.

Given the results of HER3-overexpressing tumors, it is fundamental to further explore biomarkers of response to U3-1402 in order to maximize patients benefit, especially in relation to the selection of patients according to the expression of HER3.

An analysis of safety was performed with data cut-off date of 18 Aug 2020, 182 subjects were treated with at least 1 dose of patritumab deruxtecan in the ongoing Study U31402-A-J101. All 182 subjects were female, 142 subjects (78.0%) were Asian, 34 subjects (18.0%) were White, 4 subjects (2.3%) were Black or African American, 1 subject (0.6%) was American Indian or Alaska Native, and 1 subject (0.5%) was listed as Other. The median age was 57.0 years (range: 30 years

to 83 years). Among these 182 subjects, the median duration of treatment was 5.5 months (range: 0.7 months to 30.6 months). Four subjects out of a total of 42 subjects in the Dose Escalation Part experienced 6 DLTs. In the 4.8 mg/kg dose cohort, 1 subject among 15 experienced a DLT of platelet count decreased. In the 6.4 mg/kg dose cohort, 1 subject among 15 experienced a DLT of platelet count decreased. In the 8.0 mg/kg dose cohort, 2 subjects among 6 experienced DLTs (1 subject experienced platelet count decreased, AST increased, and ALT increased; 1 subject experienced ALT increased). The MTD was not reached.

TEAEs, regardless of causality, were reported in 181 subjects (99.5%) who received patritumab deruxtecan in the ongoing Study U31402-A-J101. A total of 180 subjects (98.9%) had also experienced at least 1 TEAE considered by the investigator as related to study drug. The majority of TEAEs occurring in greater than 20% of subjects regardless of causality were gastrointestinal or hematological. There were 127 subjects (69.8%) with TEAEs of Grade ≥3 severity. The most frequently reported Grade ≥3 TEAEs (≥5% of subjects), by investigator-reported PT, were hematological.

CCI

[redacted]

As of the data cut-off date, 60 of the 182 treated subjects (33.0%) experienced at least 1 SAE. The SAEs reported in more than 1 subject, by investigator-reported PT, and in descending order of frequency are as follows: platelet count decreased (9 subjects [4.9%]); nausea, vomiting, and decreased appetite (7 subjects [3.8%] each); and febrile neutropenia, pneumonitis, thrombocytopenia, and disease progression (4 subjects [2.2%] each). Of the 182 treated subjects, 7 subjects (3.8%) experienced TEAEs with a fatal outcome.

3.5.2.2 Study U31402-A-U102

Efficacy analyses of this study reported by Jänne et al [71] showed that U3-1402 have a clinically meaningful antitumor activity, with an ORR of 39% and median PFS of 8.2 months, but also also demonstrated an increased response rate and prolonged PFS in the subset of patients who achieved early clearance of ctDNA. Confirmed responses were observed across tumors regardless of HER3 membrane H-scores, but there was a slight enrichment of confirmed responses in patients with higher HER3 membrane H-scores at baseline. The safety profile of U3-1402 was manageable, with a low rate of discontinuation due to TEAEs (9%, 7/81). Dose reduction and dose delay were successfully used in individual circumstances to mitigate toxicity and to avoid permanent discontinuation of study treatment. The most common toxicities comprised those related to gastrointestinal toxicity or cytopenia, and the most common grade ≥3 TEAEs comprised cytopenia (most commonly thrombocytopenia and neutropenia). No single TEAE was identified as a major cause of treatment discontinuation. No patient discontinued study treatment due to thrombocytopenia; when it occurred, the onset of grade ≥3 thrombocytopenia was typically early during study treatment (median time to first onset, 8 days) and was transient (median duration, 8 days). Drug-related ILD is an identified risk with DXd-based ADCs, and surveillance and early management are important in the treatment of patients with this class of therapy.

3.5.2.3 Study TOT-HER3

As of April 2021, 30 patients (all women) received the study treatment. Mean age was 52 years (range 35-77); 19 patients were pre- and 11 were post-menopausal. Median tumor size was 20 mm (range 10-60); cN0 70%; mean Ki67, 35% (range 10-90); histological grade 1-2, 67%.

Figure 8. Distribution of ERBB3 cohort, ICH expression and PAM50 subtype in the first 30 patients enrolled in TOT-HER3 trial.

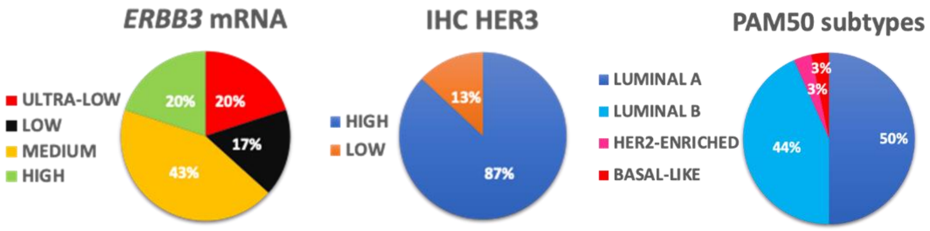

Baseline *ERBB3* mRNA levels, or HER3 protein expression, did not correlate with the change in CelTIL score. PAM50-based proliferation score did not vary significantly across *ERBB3* mRNA cohorts (p=0.39) and across IHC HER3 cohorts (p=0.32), following treatment with U3-1402.

Figure 9. CelTIL change by patient (left), indvidual changes in TILs and Celularity (right) along with *ERBB3* cohort, clinical response by palpation and intrinsic subtype in the first 30 patients enrolled in TOT-HER3 trial.

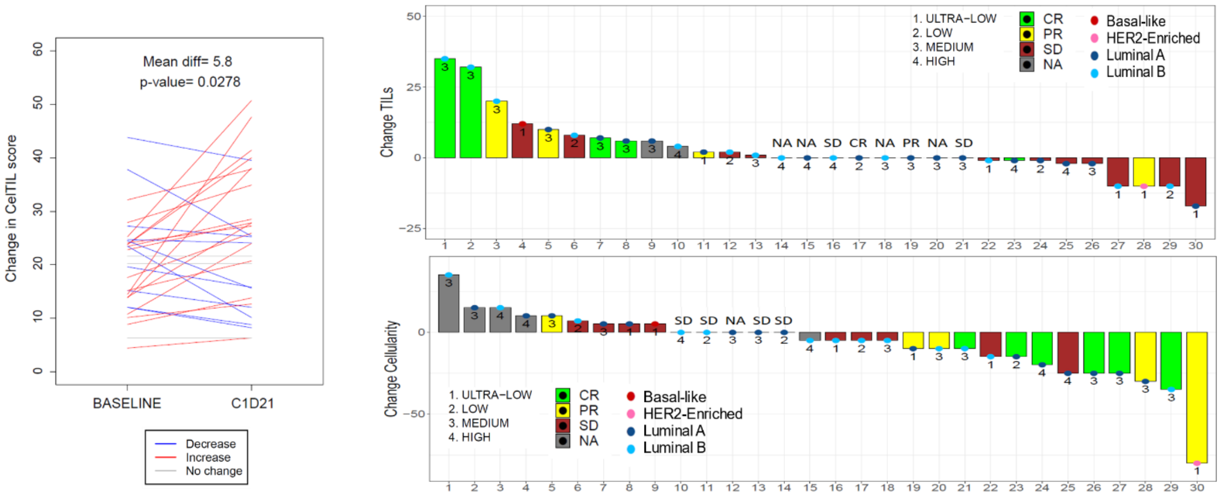

Five (38.5%) Luminal B tumors switched to Luminal A at C1D21. CelTIL score did not vary significantly between Luminal A and Luminal B tumors after single dose of U3-1402 (p=0.39). Baseline high risk-of-recurrence (PAM50 ROR) was associated with high CelTIL score at C1D21 (p=0.002). U3-1402 induced high expression of immune-related genes including *PD1*, *CD8A, CD19,* and the PAM50 Luminal A signature and suppressed proliferation-related genes

In terms of safety, 120 adverse events (AEs) occurred. In total, 29 (97%) patients reported at least one AE and 98% of all registered AEs were grade 1 or 2. A grade 3 treatment-related, reversible ALT increase occurred in 1 patient. No other treatment-related grade 3 or 4 AEs were reported.

3.6 Summary of Identified and Potential Risks

As of the data cut-off date, based on the cumulative review of the available nonclinical, clinical, and epidemiologic information for patritumab deruxtecan; same-class medications (anti-HER3 monoclonal antibodies) and the payload of patritumab deruxtecan (topoisomerase I inhibitor); and scientific literature; there was one important identified risk of ILD, and one important potential risk of elevations of aminotransferases and bilirubin (potential Hy's Law). Upon review of all clinical data and after consultation with an external cardiologist, QT prolongation and LVEF decreased were no longer considered important potential risks and AESIs for patritumab deruxtecan.

- Identified risks: anemia, neutropenia, febrile neutropenia, thrombocytopenia, leukopenia, malaise, ALT increased, AST increased, nausea, vomiting, diarrhea, PLT decreased, stomatitis, WBC count decreased, neutrophil count decreased, decreased appetite, fatigue, constipation, hypokalemia, epistaxis, and febrile neutropenia and alopecia.

- Potential risks: infusion-related reaction, dry eye, keratitis, photosensitivity, dry skin, rash, rash maculopapular, and skin pigmentation.

3.7 Immune gene-signatures in BC: role of tumor infiltrating lymphocytes (TILs)

Although BC has not been traditionally considered to be immunogenic, the association between the presence of TILs and outcome has been widely investigated in breast cancer. Immunological parameters, including tumor-infiltrating lymphocytes (TILs), have been identified as predictors of response to neoadjuvant chemotherapy in breast cancer[73].

The extent of TILs and their association with outcome vary in different BC subtypes[74,75].

Overall, TILs are more frequently observed in highly proliferative tumors (e.g. TNBC and HER2þ BC), and, in these tumors, TILs are usually considered to predict better outcome[76]; and have proven prognostic value in triple-negative and HER2-positive BC patients, both in the adjuvant and neoadjuvant settings[77,78]. In the early stage HER2-positive (HER2+) and in triple negative breast cancer (TNBC), immune infiltrates are detectable in up to 75% of tumors, with up to 20% of tumors having a particularly dense infiltrate and with lower amount of TILs in luminal subtypes[79].

Denkert et al[80] found that a high content of TILs conferred a significantly (p = 0.003) higher likelihood of pathological complete response in HR positive BC patients enrolled in the GeparDuo and GeparTrio Trials of neoadjuvant chemotherapy.

In the neoadjuvant setting, it has been reported that the extent of TILs in residual disease (RD) after NAC is associated with better prognosis in patients with TNBC. It is worth mentioning that a high conversion rate from TILs negative to TILs-positive has been observed from core biopsies before NAT and samples obtained at surgery after NAC[81]. A four-gene signature from pre-treatment samples predicts high levels of TILs after NAT and good outcome in patients with TNBC, thus adding prognostic information to a clinicopathological model at diagnosis[82].

Although there is evidence of the prognostic value of TILs, only a limited number of studies are investigating the importance of the subsets of T cells in TILs and the significance of the location of these TILs. In one study in TNBC[83], the number of CD4+ or CD8+ T cells at tumor host interface or intratumoral stroma was not significantly associated with distant relapse-free or overall survival. However, the CD4/CD8 ratio at the tumor-host interface was significantly associated with both relapse-free survival (hazard ratio 0.2, P = .002) and overall survival (hazard ratio 0.13, P = .002).

TILs should be considered as just one piece of the immunogenic puzzle of the tumor and thus their assessment complementary to other approaches as tumor cellularity in translational research. More clinical studies are necessary to determine to what extent TILs solely or combined with other morphological (for example, subtyping) or genomics-based variables (for example, gene expression profiles) will prove to be sufficiently robust, in terms of clinical utility, to be implemented in a daily practice setting.

3.8 Tumor cellularity and tumor-infiltrating lymphocytes (CelTIL score) as a biomarker of response

3.8.1 An early biological readout of drug activity: tumor cellularity and tumor-infiltrating lymphocytes (CelTIL score)

Tumor microenvironment, in particular, stromal tumor-infiltrating lymphocytes (TILs) is a consistent biomarker of a better outcome. The presence of (TILs) is associated with increased pathologic complete response (pCR) and improved outcomes in HER2-positive early-breast cancer treated with anti-HER2-based therapy[74].

TILs seem to be interesting also as an on-treatment biomarker, in fact, there is a correlation between increasing TILs during therapy and pCR. In PAMELA trial (trastuzumab and lapatinib in the absence of chemotherapy), baseline and Day15 TILs were significantly associated with pCR in univariate analysis following trastuzumab and lapatinib[84]. In multivariable analysis, D15 TILs, but not baseline TILs, were significantly associated with pCR. At D15, TILs and tumor cellularity were found independently associated with pCR. A combined score (CelTIL) considering both variables were derived in PAMELA trial.

Unscaled tumor cellularity and TILs (CelTIL) score = −0.8 × tumor cellularity (in %) + 1.3 × TILs (in %). The minimum and maximum unscaled CelTIL scores were −80 and 130, respectively.

In the PAMELA trial, CelTIL at D15 as a continuous variable was significantly associated with pCR, and patients with CelTIL-low and CelTIL-high scores (cut point 33.16) had a pCR rate of 0% and 33%, respectively. In LPT109096 trial (validation dataset)[84], CelTIL at D15 was found associated with pCR following anti-HER2-based chemotherapy both as a continuous variable, and as group categories, using a pre-defined cut-off (75.0% versus 33.3%). Thus, CelTIL at D15 provides independent predictive information upon completion of neoadjuvant anti-HER2-based therapy.

To further explore if CelTIL is a biomarker of early response to any highly active treatment, we evaluated CelTIL in SOLTI's NEOERIBULIN trial[85], where patients with HER2-negative disease (i.e. triple-negative or HR-positive/HER2-negative disease) were treated with eribulin monotherapy in the neoadjuvant setting. CelTIL was evaluated in a biopsy before Cycle 2 Day 1. As expected, patients without a pCR showed significantly lower CelTIL scores than patients with a pCR (p=0.049). Thus, these data, together with the previous results in PAMELA trial, further suggest that CelTIL is a valid biomarker of treatment response regardless of the type of treatment.

3.8.2 Variability of the CelTIL score according to therapy.

At SOLTI, 4 trials (VENTANA[86], PAMELA[87], CORALLEEN[88] and NEOERIBULIN[89]) in early breast cancer have now evaluated CelTIL score before and after 2-3 weeks of therapy in 403 patients.

In the VENTANA trial, 40 patients with untreated stage I-III breast cancer received letrozole or letrozole plus metronomic vinorelbine (50mg 3 days a week) for 3 weeks and then surgery or a biopsy was performed. The study showed that vinorelbine did not increase the biological efficacy of letrozole alone, which showed a clear downregulation of proliferation as measured by Ki67 or gene expression. In terms of CelTIL, no consistent changes were observed and the mean CelTIL change was +0.0 (**Figure. 10A**). This was expected since letrozole´s effect is mostly anti-proliferative and not pro-apoptotic. The other 3 trials allowed linking CelTIL changes with response to neoadjuvant therapy. In CORALLEEN trial, 106 patients with Luminal B/HER2-negative disease were randomized to 6-months of letrozole plus ribociclib or AC followed by weekly paclitaxel. A biopsy at week 2 was performed. As expected, due to its mostly cytostatic effect, letrozole plus ribociclib did not consistently show an increase in CelTIL (**Figure. 10B**). Regarding 1 dose of AC, a very small mean increase in CelTIL of +2.2 was observed. As expected, none of the changes in CelTIL in the two arms were found associated with response (defined as PAM50 ROR-low at surgery) (**Figure. 10C**).

The third trial was NEOERIBULIN. As explained before, this trial treated 137 patients with HER2-negative disease (i.e., 85 HR+ and 52 triple-negative) with eribulin monotherapy for 4 cycles. A biopsy at baseline and before cycle 2 day 1 (week 3) was performed. In this trial, a significant mean increase of +8.1 in CelTIL was observed in all patients and CelTIL at week 3 was associated with pCR, as reported above, as well as overall response (p<0.001) by imaging before surgery. This mean increase of CelTIL was observed in both HR+ and triple-negative disease (**Figure. 10B and 10C**) and the association of CelTIL at week 3 and response by imaging was also observed in both groups, although in HR+ disease it did not reach statistical significance (p=0.082).

Finally, the PAMELA trial showed the largest mean increase of CelTIL of +31.4, regardless of HR status. The association with pCR was also found independently of HR status. A summary of the results obtained from CelTIL across the 4 trials can be found in **Table 5**.

Figure 10. Changes of CelTIL and association with response across SOLTI trials.

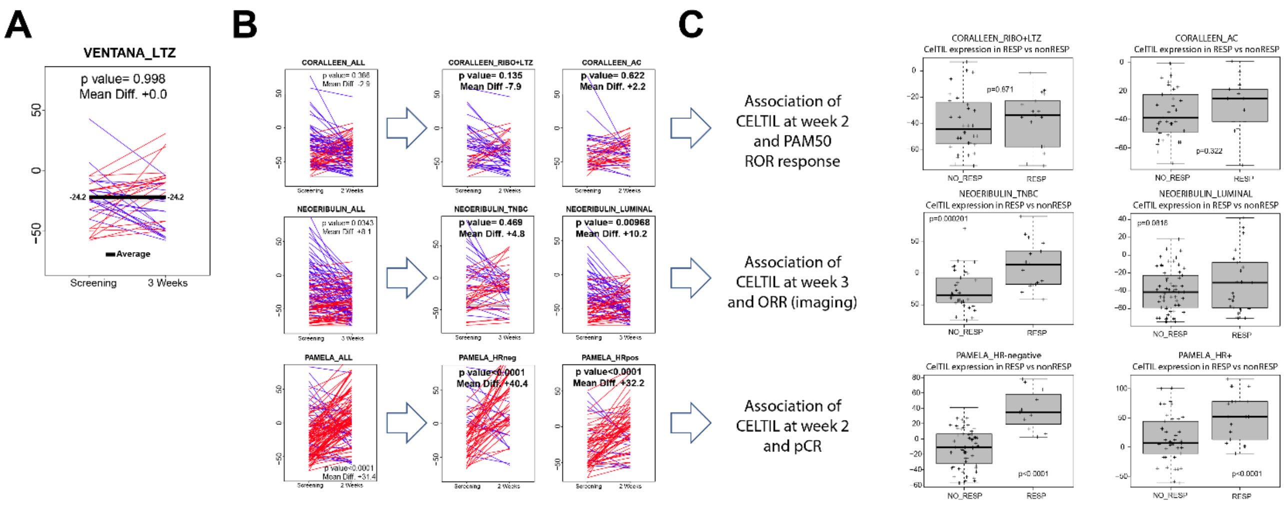

Table 5. Summary of the results obtained with absolute changes in CelTIL across 4 SOLTI clinical trials.

				ABSOLUTE CHANGE_CELTIL					
	N	CT	TYPE_TREATMENT	TIMEPOINT	MEDIANA	MEAN	STD_DEV	95% CI LOW	95% CI HIGH
**NEOERIBULIN**	137	YES	ERIBULIN	WEEK3	1.6	8.1	33.6	2.4	13.8
NEOERIB_LUM	85	YES	ERIBULIN	WEEK3	4.0	10.2	29.8	3.7	16.6
NEOERIB_TNBC	52	YES	ERIBULIN	WEEK3	-0.8	4.8	39.1	-6.1	15.7
**VENTANA**	39	NO	LETROZOLE*	WEEK3	0.0	0.0	27.3	-8.9	8.9
**PAMELA**	130	NO	LAPATINIB+TRASTUZUMAB+/-ENDOCRINE	WEEK2	28.6	31.4	44.6	23.7	39.2
PAMELA_RH+	70	NO	LAPATINIB+TRASTUZUMAB+ENDOCRINE	WEEK2	20.0	23.7	32.2	16.1	31.4
PAMELA_RH-neg	60	NO	LAPATINIB+TRASTUZUMAB	WEEK2	45.3	40.4	54.6	26.3	54.5
**CORALLEN**	97	-	-	WEEK2	-1.3	-2.9	29.1	-8.8	3.0
CORALLEN_QT	48	YES	AC	WEEK2	3.7	2.2	27.6	-5.8	10.2
CORALLEN_RIBO+LTZ	49	NO	RIBOCICLIB+LETROZOLE	WEEK2	-8.0	-7.9	29.9	-16.5	0.7

*, STD_DEV, standard deviation.

3.9 Dose Selection Rationale

TOT-HER3 trial was initially designed to test a dose of 6.4 mg/kg in the early setting of breast cancer within the framework of a window of opportunity trial. As of 14 of February of 2022, a total of 78 patients have been treated with one dose of U3-1402 on this trial and have shown a reasonable safety profile, accordingly with previous available data.

Currently, two new cohorts of patients are included, a cohort of patients with a early TNBC and a new cohort of HR+, HER2-negative patients to be treated with a dose of 5.6 mg/kg.

A dose of 5.6 mg/kg Q3W is the recommended dose for further monotherapy clinical studies with patritumab deruxtecan, according to investigator brochure of the drug.

3.10 Study Rationale

HER2 testing and targeted therapy represents a critical cornerstone in the management of breast cancer.

HER3, a member of the HER receptor tyrosine kinase family, is overexpressed in breast cancer and in other solid tumors, as melanoma, colorectal, gastric, ovarian, and pancreatic cancers[90–92].

In breast cancer, HER-3 status is an important prognostic marker of disease-specific survival and overexpression is associated with poor prognosis[91]. Although many drugs against HER3 are in clinical development, there is no approved targeted treatment yet.

U3-1402 is a novel ADC comprised of a fully humanized anti-HER3 antibody (patritumab) covalently conjugated via a cleavable peptide linker to a derivative of the topoisomerase I inhibitor exatecan (deruxtecan). After binding to HER3 on the tumor cell surface, U3-1402 is internalized and leads to apoptosis via inhibition of topoisomerase I.

CCI [redacted]

Preliminary data demonstrated antitumor activity in a substantial number of heavily pretreated HER3-expressing metastatic breast cancer patients, and associated with a manageable safety profile[94], as well as in in heavily pretreated subjects with metastatic or locally advanced EGFRm NSCLC [71].Window of opportunity studies, in which treatment-naïve patients are briefly exposed to therapy to observe tumor biology, are emerging as a strategy to identify biomarkers. The rationale for this window is to have the opportunity to analyze the clinical activity of a HER3-targeting antibody-drug conjugate alone without the interference of concomitant therapy. The 3-week period is felt both to be safe to the patient (based on our previous experience with similar protocols) and to provide early and critically important biological information of clinical response. Due to the need to better understand the molecular biology, tumor microenvironment and overcoming resistance mechanisms in breast cancer and based on the clinical and nonclinical data available for U3-1402, this window-of-opportunity study is designed.

4. Study objectives and endpoints

4.1 Primary objective and endpoint

Primary objective	Primary endpoint
To evaluate changes in CelTIL score after one dose of 6.4 mg/kg of U3-1402 in patients with HR+/HER2-negative early breast cancer group.	Mean change in CelTIL score per central assessment between paired samples (baseline and C1D21)<br><br>CelTIL score = −0.8 × tumor cellularity (in %) + 1.3 × TILs (in %). The minimum and maximum unscaled CelTIL scores will be −80 and 130. This unscaled CelTIL score will then be scaled to reflect a range from 0 to 100 points.

4.2 Secondary objectives and endpoints

Secondary objectives	Secondary endpoints
To evaluate changes in CelTIL score in the following groups:<br>• In each *ERBB3* cohort of HR+/HER2-negative early breast patients treated with one 6.4 mg/kg dose.<br>• In TNBC patients treated with one 5.6 mg/kg dose<br>• In HR+/HER2-negative early breast patients treated with one 5.6 mg/kg dose<br>• In all treated patients	Mean change in CelTIL score per central assessment between paired samples (baseline and C1D21)<br><br>CelTIL score = −0.8 × tumor cellularity (in %) + 1.3 × TILs (in %). The minimum and maximum unscaled CelTIL scores will be −80 and 130. This unscaled CelTIL score will then be scaled to reflect a range from 0 to 100 points.
To evaluate the association of baseline *ERBB3* expression with changes in CelTIL score in the following groups:<br>• In HR+/HER2-negative early breast patients treated with one 6.4 mg/kg dose and within each *ERBB3* cohort	Correlation between ERBB3 mRNA baseline levels and changes in CelTIL score at C1D21.

• In TNBC patients treated with one 5.6 mg/kg dose<br>• In HR+/HER2-negative early breast patients treated with one 5.6 mg/kg dose<br>• In all treated patients	
To evaluate the association of baseline HER3 IHC expression with changes in CelTIL score in the following groups:<br>• In HR+/HER2-negative early breast patients treated with one 6.4 mg/kg dose and within each *ERBB3* cohort<br>• InTNBC patients treated with one 5.6 mg/kg dose<br>• In HR+/HER2-negative early breast patients treated with one 5.6 mg/kg dose<br>• In all treated patients	Correlation between baseline HER3 IHC levels per central assessment and changes in CelTIL score at C1D21 in paired samples.
To evaluate the changes in CelTIL across the four PAM50 intrinsic subtypes.	Mean change in CelTIL score per central assessment between paired samples (baseline and C1D21) according to intrinsic subtype: Luminal A, Luminal B, HER2-enriched and Basal-like subtypes.
To evaluate the antiproliferative activity of one dose of U3-1402 between baseline and post-treatment samples.	Complete Cell Cycle Arrest (CCCA) determined per central assessment by IHC Ki67 (i. as a continuous variable and ii. as a % of <2.7%) between paired samples (baseline and C1D21).<br><br>Differences in differential expression (mean suppression = 100 - [geometric mean (post treatment / pre-treatment · 100)]) of proliferative genes (BIRC5, CCNB1, CDC20, CDCA1, CEP55, KNTC2, MKI67, PTTG1, RRM2, TYMS and UBE2C)
To evaluate the association of *ERBB3* mRNA expression with HER3 IHC expression.	Correlation coefficients between both biomarkers.
To evaluate the changes of HER3 expression.	HER3 IHC at baseline, at D3-D7 (optional), C1D21.

To evaluate clinical activity of one dose of U3-1402 between baseline and post-treatment.	Overall response rate (ORR) defined as the proportion of patients who have a partial or complete response evaluated with ultrasound and clinical palpation at C1D21, according to modified RECIST1.1 criteria (appendix E)
To describe the safety and tolerability of U3-1402.	Type, incidence, severity (as graded by the NCI CTCAE v. 5.0), seriousness and attribution to the study medications of AEs and any laboratory abnormalities.

4.3 Exploratory objectives and endpoints

CCI [redacted]

CCI

[redacted]

5. Study design

5.1 Study design

This is a prospective, multicenter, single arm, window-of-opportunity study evaluating the biological effect of U3-1402 in treatment naïve patients with early breast cancer, whose primary tumors are ≥1 cm by ultrasound evaluation.

The primary objective is to evaluate the biological activity of U3-1402, measured as the CelTIL score increase at post-treatment (C1D21) in HR+/HER2-negative BC included patients.

The study will consist of 2 parts enrolling ~115 patients.

- **Part A** will target to treat, with 6.4 mg/kg dose, 80 patients with HR-positive/HER2-negative tumors and
- **Part B** will target to treat with 5.6 mg/kg dose 20 patients with HR-positive/HER2-negative and 15 patients with TNBC tumors

Part A will test U3-1402 in patients with HR-positive/HER2-negative early breast cancer with a dose of 6.4 mg/kg. Part B will consist in testing 5.6 mg/kg dose of U3-1402 in patients with HR-positive/HER2-negative early breast cancer and in triple-negative early breast cancer and will be performed sequentially after Part A.

A signed, written Informed Consent Form (ICF) must be obtained before any study specific assessment is initiated. However, procedures conducted as part of the subject's routine clinical management (e.g., diagnostic imaging studies) obtained prior to signing of the IC may be utilized for screening or baseline purposes provided these procedures are conducted as specified in the protocol and within the protocol-defined timeframes.

All patients will undergo pretreatment tumor tissue acquisition. Enrollment will be carried out in a one-stage design. At least two core biopsy specimens are required at baseline to be placed in 10% formalin.

During Part A: ER, PgR and HER2 stsatus will be defined according to the most recent ASCO/CAP and analysed by local laboratory. Ki67% ≥ 10% by local determination will be necessary to include patients with HR-positive/HER2-negative tumors. Central determination of ERBB3 mRNA expression will be performed in FFPE core-biopsies and patients will be enrolled in four cohorts, according to the expression of ERBB3 based in quartiles and defined by the pre-specified cutoff, to ensure a broad representation of HR-positive/HER2-negative tumors with different ERBB3 expression. The number of slots available per cohort will be limited to 20. Once the Sponsor communicates that the last patient of a determinate cohort has been included, it will remain closed to further recruitment.

- Cohort 1: Ultra-Low ERBB3 expression
- Cohort 2: Low ERRB3 expression

- Cohort 3: Medium ERBB3 expression
- Cohort 4: High ERBB3 expression

During Part B: ER, PgR and HER2 status will be defined according to the most recent ASCO/CAP and analysed by local laboratory. Ki67% ≥ 10% by local determination. Central determination of ERBB3 mRNA expression will be performed in FFPE core biopsies retrospectively, and will not be needed for inclusion purposes.

After confirmation of all the eligibility criteria, patients will be enrolled and a single dose of U3-1402 will be administered by intravenous infusion at a dose of 6.4 mg/kg or 5.6 mg/kg as described before.

A second biopsy post-treatment of the same lesion will be mandatory at C1D21 visit, independent of the subsequent treatment.

Thereafter, patients will be considered either for definitive surgery or primary medical treatment (e.g. neoadjuvant chemotherapy) at the discretion of the treating physician.

A minimum of one (two recommended if possible) core of tumor tissue will be collected at each time point for all patients that will be Formalin-Fixed and Paraffin-Embedded (FFPE). In case of multicentricity and/or multifocality, samples will always be collected from the lesion that was defined as “target lesion” at the time of the enrollment. Post-treatment biopsies will be collected for histological examination to assess CelTIL score and other endpoint analyses. Given the preliminary results form the interim analysis, where clinical responses was observed by clinical palpation on day 21, an ultrasound evaluation will be performed not only at screening but also at C1D21.

Blood samples will be collected throughout the study at the following time points, C1D1, C1D3-D7, C1D21 and the end of study (EOS) for future exploratory endpoints and genomic analyses.

A safety follow-up, at the EOS/SFV visit, will be done at 49 days (± 14 days) after the administration of U3-1402. Follow-up of AEs related to the investigational product will be performed until their resolution, including Lab tests if necessary.

Given the results from the interim analysis in tumor size reduction and changes in tumor biology, we considered relevant to collect surgery outcome details (including pathologiacal staging) and post-operative treatment information (chemotherapy, radiotherapy, etc.) will be collected at EOS/SFV visit.

An additional Follow-up will be done after 10 months of treatment.

A schedule of assessments is provided in **Appendix 1.**

A first safety-only interim analysis will be performed after the first 10 patients have been treated and have available safety data; decisions on study continuation will be made by the Study Steering Committee. Safety and efficacy interim analysis will be performed after the first 30 patients are available for central CELTIL evaluation during Part A and decisions on study continuation will be made by the Study Steering Committee. As the safety of the treatment has been evaluated in the metastatic setting, NO interruption in the accrual will be done, in order to maintain the dynamic of accrual in the trial.

After completing the Part A of the study, the Steering Committee of the study will review all study data. Recommendations to continue the recruitment with the Part B of the study or permanently suspend the study will be based on this analysis.

5.2 Trial diagram

Figure 1 Study Design

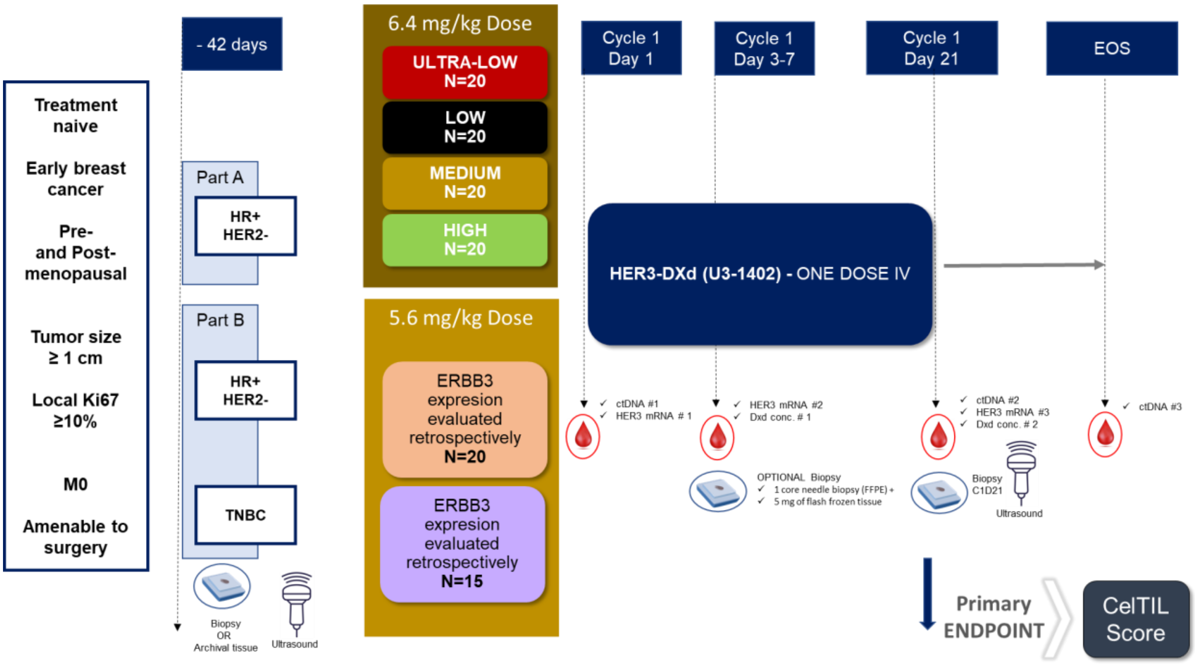

6. Study Population

6.1 Inclusion criteria

All the following criteria must be fulfilled for a patient to be eligible for this study:

1. Written ICF for all study procedures according to local regulatory requirements prior to beginning specific protocol procedures.
2. Premenopausal or postmenopausal women and men, age ≥ 18 years.
3. ECOG Performance Status 0 - 1.
4. Histologically confirmed non-metastatic primary invasive adenocarcinoma of the breast untreated and recently diagnosed, with all the following characteristics:

- At least one lesion that can be measured in at least 1 dimension with ≥ 1 cm in largest diameter measured by ultrasound or MRI.
- Absence of distant metastasis (M0) as determined by institutional practice.
- In the case of a multifocal or multicentric tumors, the largest lesion must be ≥ 1 cm and designated the “target” lesion for all subsequent tumor evaluations and biopsies.

5. Patient must have biopsiable disease.
6. **<u>Only for HR+/HER2-negative patients</u>:** Estrogen (ER)-positive and/or Progesterone (PgR)-positive and HER2-negative tumor by the most recent American Society of Clinical Oncology - College of American Pathologists (ASCO-CAP) guidelines: ER and PgR defined as IHC nuclear staining ≥1% and HER2 negative locally assessed. **<u>Only for TNBC patients</u>:** Estrogen (ER)-negative and Progesterone (PgR)-negative and HER2-negative tumor by the most recent American Society of Clinical Oncology - College of American Pathologists (ASCO-CAP) guidelines: ER and PgR defined as IHC nuclear staining <1% and HER2 negative locally assessed
7. Ki67% ≥ 10% locally assessed (Dowsset et al. *Journal of the National Cancer Institute,* 103 (22), 1656-1664. 2011).
8. Available pre-treatment FFPE core needle biopsy evaluable for PAM50 and ERBB3 mRNA expression. Minimal sample requirements are to have at least 2 tumor cylinders with a minimal tissue surface of 10 mm2 tissue, containing at least 30% tumor cells and having enough tissue to do at least one 3 µm slide, nine 4 µm slides, and eighteen 10 µm slides. Macrodissection is allowed when needed. If archival tissue is either insufficient or unavailable, a new biopsy from the pretreated tumor must be obtained. Patients whose tumor tissue is not evaluable for ERBB3 expression central testing are not eligible.

9. Baseline LVEF ≥ 50% measured by echocardiography (ECHO) or Multiple Gate Acquisition (MUGA) scan

10. Adequate organ function, as determined by the following laboratory tests prior to enrollment:

    - Hematological

        - Absolute neutrophil count (ANC) ≥ 1.5 x 10^9/L

        - Platelet count ≥ 100 x 10^9/L (platelet transfusions are not allowed up to 14 days prior to Cycle 1 Day 1 to meet eligibility criteria)

        - Hemoglobin ≥ 9 g/dL (red blood cell transfusion and/or erythropoietin allowed)

    - Renal

        - Serum creatinine ≤ 1.5 x upper limit of normal (ULN), or 24-hour creatinine clearance ≥ 60 mL/min for subject with creatinine levels > 1.5 x ULN. (Note: Creatinine clearance does not need to be determined if the baseline serum creatinine is within normal limits. Creatinine clearance should be calculated per institutional standard).

    - Hepatic

        - Serum bilirubin ≤ 1.5 x ULN OR direct bilirubin ≤ ULN for a subject with total bilirubin level > 1.5 x ULN

        - Aspartate aminotransferase (AST) ≤ 3 x ULN

        - Alanine aminotransferase (ALT) ≤ 3 x ULN

        - Coagulation International normalization ratio (INR) or prothrombin time (PT) ≤ 1.5 x ULN

        - Partial thromboplastin time (PTT) or activated PTT (aPTT) ≤ 1.5 x ULN

11. Absence of any psychological, familial, sociological or geographical condition potentially hampering compliance with the study protocol and follow-up schedule; those conditions should be discussed with the patient before registration in the trial.

12. Female subject of childbearing potential should have a negative urine or serum pregnancy test within 7 days prior to enrollment. If urine pregnancy test is positive or cannot be confirmed as negative, a serum pregnancy test will be required. Pregnancy testing does not need to be pursued in patients who are judged as postmenopausal before enrollment, as determined by local practice, or who have undergone bilateral oophorectomy, total hysterectomy, or bilateral tubal ligation. Women of childbearing potentially enrolled to the treatment must use adequate contraception for the duration of protocol treatment and after 7 months after the study drug administration (see Appendix B).

6.2 Exclusion criteria

1. Inoperable locally advanced or inflammatory (i.e., inoperable Stage III) breast cancer.
2. Metastatic (Stage IV) breast cancer.
3. Bilateral invasive breast cancer.
4. Patients in whom a primary tumor excisional biopsy was performed.
5. Any prior treatment for primary actual invasive breast cancer.
6. Prior treatment with a HER3 antibody, topoisomerase I inhibitor, with an ADC which consists of an exatecan derivative that is a topoisomerase I inhibitor (e.g., DS-8201) and with a govitecan derivative (e.g., IMMU-132).
7. Medical history of symptomatic congestive heart failure (New York Heart Association classes II-IV) or serious cardiac arrhythmia requiring treatment; myocardial infarction within 6 months prior to enrollment or unstable angina.
8. QT interval corrected using Fridericia's formula to > 450 millisecond (ms) in males and > 470 ms in females.
9. Any factors that increase the risk of corrected QT (QTc) interval prolongation or risk of arrhythmic events, such as congenital long QT syndrome, family history of long QT syndrome, or unexplained sudden death under 40 years of age in first-degree relatives.
10. Medical history of clinically significant lung diseases (e.g., interstitial pneumonia, pneumonitis, pulmonary fibrosis, and severe radiation pneumonitis) or who are suspected to have these diseases by imaging at screening period.
11. Clinically significant corneal disease.
12. Major surgical procedure or significant traumatic injury within 28 days prior to enrollment.
13. Assessment by the investigator to be unable or unwilling to comply with the requirements of the protocol.
14. History of other malignancy within the last 3 years, except for appropriately treated carcinoma in situ of the cervix, non-melanoma skin carcinoma, Stage I uterine cancer, or other malignancies with an expected curative outcome.
15. Current severe, uncontrolled systemic disease (e.g. clinically significant cardiovascular, pulmonary or metabolic disease; wound healing disorders; ulcers; bone fractures).
16. Concurrent, serious, uncontrolled infections or current known infection with HIV or active hepatitis B and/or hepatitis C.

17. History of significant co-morbidities that, in the judgment of the investigator, may interfere with the conduction of the study, the evaluation of response, or with ICF.

18. Known hypersensitivity to either the drug substance components (including an antibody, a drug-linker, or a topoisomerase I inhibitor) or inactive ingredients in the drug product or history of severe hypersensitivity reactions to other monoclonal antibodies.

19. Clinically severe pulmonary compromise resulting from intercurrent pulmonary illnesses including, but not limited to, any underlying pulmonary disorder (i.e. pulmonary emboli within three months of the study enrollment, severe asthma, severe COPD, restrictive lung disease, pleural effusion etc.), and any autoimmune, connective tissue or inflammatory disorders with potential pulmonary involvement (i.e. rheumatoid arthritis, Sjögren's syndrome, sarcoidosis etc.), or prior pneumonectomy.

20. Has unresolved toxicities from previous anticancer therapy, defined as toxicities (other than alopecia) not yet resolved to National Cancer Institute Common Terminology Criteria for Adverse Events (NCI-CTCAE) version 5.0, grade ≤1 or baseline. Subjects with chronic grade 2 toxicities may be eligible per the discretion of the Investigator.

21. Is receiving chronic systemic corticosteroids dosed at >10 mg prednisone or equivalent anti-inflammatory activity or any form of immunosuppressive therapy prior to Cycle 1 Day 1. Subjects who require use of bronchodilators, inhaled or topical steroids, or local steroid injections may be included in the study.

7. Study treatment

[redacted]

[redacted]

7.2.2 Dosage and Administration

CCI

[redacted]

7.2.3 Adverse events

Please see section 2.5.2 for summarize safety data from previous studies, section 2.6 for identified and potential risks, section 6.3 for allowed concomitant medication (i.e. pre-medication) and section 6.2.4 for toxicity management.

7.2.4 Toxicity management for AEs associated with U3-1402

Toxicity management guidelines for specifics AEs associated with U3-1402 are provided in **Table 7**

Table 7- Toxicity management guidelines for AEs associated with U3-1402

	Worst toxicity CTCAE version 5.0 Grade (unless otherwise specified)	Toxicity management
**General disorders and administration site conditions**		
**Infusion-related Reaction**	Grade 1 (Mild transient reaction; infusion interruption not indicated; intervention not indicated)	If infusion-related reaction (such as fever and chills, with and without nausea/vomiting, pain, headache, dizziness, dyspnea, hypotension) is observed during administration, the infusion rate should be reduced by 50% and subjects should be closely monitored.
	Grade 2 (Therapy or infusion interruption indicated but responds promptly to symptomatic treatment (e.g., antihistamines, nonsteroidal anti-inflammatory drugs (NSAIDs), narcotics, IV fluids); prophylactic medications indicated for ≤ 24 h)	Administration of U3-1402 should be interrupted and symptomatic treatment started (e.g., antihistamines, NSAIDs, narcotics, IV fluids).<br>If the event resolves, infusion can be re-started at a 50% reduced infusion rate.
	Grade 3 or 4 (Prolonged or life-threatening consequences, urgent intervention indicated)	Administration of U3-1402 should be discontinued immediately and permanently.<br>Urgent intervention indicated. Antihistamines, steroids, epinephrine, bronchodilators, vasopressors, IV fluid therapy, oxygen inhalation etc., should be administered.
**Respiratory, thoracic, and mediastinal disorders**		
**Respiratory, thoracic and mediastinal disorders**	See next rows	If a subject develops an acute onset of new or worsening pulmonary or other related signs/symptoms such as dyspnea, cough or fever, rule out ILD/pneumonitis.<br><br>If the AE is suspected to be ILD/pneumonitis, treatment with study treatment should be interrupted pending further evaluations.<br>Evaluations should include:<br>* High resolution computed tomography (CT)<br>* Pulmonologist consultation<br>* Pulmonary function tests and pulse oximetry (SpO2)<br>* Arterial blood gases if clinically indicated<br>* One blood sample collection for PK and exploratory biomarker analysis as soon as ILD/pneumonitis is suspected, if feasible

	Worst toxicity CTCAE version 5.0 Grade (unless otherwise specified)	Toxicity management
		* Other tests could be considered as needed.<br>As soon as ILD/pneumonitis is suspected, corticosteroid treatment should be started promptly as per indications in this table<br>All events of ILD regardless of severity or seriousness will be followed until resolution.
**ILD/pneumonitis**	Grade 1	Toxicity Management:<br>Monitor and closely follow-up in 2 to 7 days for onset of clinical symptoms and pulse oximetry.<br>Consider follow-up imaging in 1-2 weeks (or as clinically indicated).<br>Consider starting systemic steroids (e.g. at least 0.5 mg/kg/day prednisone or equivalent) until improvement, followed by gradual taper over at least 4 weeks.<br>If worsening of diagnostic observations despite initiation of corticosteroids, then follow Grade 2 guidelines (if patient is asymptomatic, then patient should still be considered as Grade 1 even if steroid treatment is given).
**ILD/pneumonitis**	Grade 2	Toxicity Management:<br>Promptly start systemic steroids (e.g., at least 1 mg/kg/day prednisone or equivalent) until clinical improvement, followed by gradual taper over at least 4 weeks.<br>Monitor symptoms closely.<br>Re-image as clinically indicated.<br>If worsening or no improvement in clinical or diagnostic observations in 5 days,<br>Consider increasing dose of steroids (e.g., 2 mg/kg/day prednisone or equivalent) and administration may be switched to intravenous (e.g. methylprednisolone).<br>Re-consider additional work-up for alternative etiologies as described above.<br>Escalate care as clinically indicated.
**ILD/pneumonitis**	Grade 3 and 4	Toxicity Management:<br>Hospitalization required.<br>Promptly initiate empiric high-dose methylprednisolone intravenous treatment

	Worst toxicity CTCAE version 5.0 Grade (unless otherwise specified)	Toxicity management
		(e.g., 500-1000 mg/day for 3 days), followed by at least 1.0 mg/kg/day of prednisone (or equivalent) for a minimum of 14 days or until complete resolution of clinical symptoms and chest CT findings, followed by gradual taper over at least 4 weeks<br>Re-image as clinically indicated.<br>If still no improvement within 3 to 5 days,<br>Re-consider additional work-up for alternative etiologies as described above.<br>Consider other immuno-suppressants and/or treat per local practice.
**Gastrointestinal disorders**		
**Nausea and/or vomiting and/or Diarrhea**		Nausea and/or vomiting and/or Diarrhea should be controlled with standard antiemetics and/or anti-diarrheal medicines. Metoclopramide standard antiemetic products may be used as the first drug of choice. If a 5HT3 inhibitor needs to be incorporated because the Metoclopramide is insufficient, the use of Granisetron 1 mg/12h will be permitted due to the low capacity for prolonging QTc with respect to Ondansetron.
**Eye disorders**		
**Ocular Toxicity**		The ocular toxicity should be monitored by an ophthalmologist (or qualified delegate) for recovery.
**Blood and lymphatic system disorders**		
**Neutrophil count decreased**	Grade 3 (500 to <1000/mm^3)	Monitor neutrophil lab value until resolution to within normal limits or to baseline values.
	Grade 4 (<500/mm^3)	
**Febrile neutropenia (ANC <1 × 10^9/L, fever >38.3°C or a sustained temperature of ≥38°C for more than 1 hour)**	Any Grade	Monitor neutrophil lab value until resolution to within normal limits or to baseline values.<br><br>Standard management of febrile neutropenia. per clinical treatment guidelines.

	Worst toxicity CTCAE version 5.0 Grade (unless otherwise specified)	Toxicity management
**Anemia**	Grade 3 (Hb <8.0 g/dL)	Monitor hemoglobin lab value until resolution to within normal limits or to baseline values.<br><br>Consider transfusion per institutional guidelines.
	Grade 4 (Life-threatening consequences; urgent intervention indicated)	
**Platelet count decreased**	Grade 3 (Plt <50 - 25 × 10^9/L)	Monitor platelet values until resolution to within normal limits or to baseline values.<br><br>Consider transfusion per institutional guidelines.
	Grade 4 (Plt <25 × 10^9/L)	
**Lymphocyte Count Decreased**	Grade 4 (<0.2 × 10^9/L)	Monitor Lymphocyte values until resolution to within normal limits or to baseline values.

<u>Special precautions:</u>

Pregnancy and Lactation

No reproductive and developmental toxicity studies have been conducted to date, and the teratogenic potential of U3-1402 has not been established. Based on the composition and mechanism of action of the product, as well as data from similar products of the same class, a teratogenic potential cannot be excluded. U3-1402 should not be administered to pregnant women, to women attempting to conceive or who are breastfeeding, or to men who have a sexual partner who may conceive. Females should not be pregnant or become pregnant or be nursing. Patients of childbearing potential should agree to use effective contraception (Appendix B).

7.3 Concomitant therapy

Concomitant therapy includes any medication (e.g., prescription drugs, over-the counter drugs, herbal or homeopathic remedies, nutritional supplements) used by a patient from 7 days prior to screening to the treatment discontinuation visit. All such medications should be reported to the Investigator and recorded on the Concomitant Medications e-CRF.

No formal drug-drug interaction studies with U3-1402 have been conducted, and the risk of drug-drug interactions has not been assessed in humans.

Prohibited therapy

The following therapies are not permitted on the study:

- Anti-cancer therapy: No additional investigational or commercial anti-cancer agents such as chemotherapy, immunotherapy, targeted therapy, biological response modifiers, or endocrine therapy will be allowed.
- Radiation therapy: Radiation therapy should not be administered to the breast and/or regional lymph nodes prior to 2nd biopsy in this study.
- Concomitant use of CYP3A4 strong inhibitors (e.g., boceprevir, clarithromycin, itraconazole, ketoconazole, lopinavir/ritonavir, nefazodone, telaprevir, telithromycin, voriconazole) or Organic anion transporting polypeptides (OATP) inhibitors (e.g., lopinavir/ritonavir, cyclosporine, rifampicin) should be avoided.

If concomitant use of strong CYP3A4 or OATP inhibitors is unavoidable, consider delaying U3-1402 treatment until the strong CYP3A4 or the OATP inhibitors have cleared from the circulation (approximately 3 elimination half-lives of the inhibitors) when possible.

- If a strong CYP3A4 or an OATP inhibitor is co-administered and U3-1402 treatment cannot be delayed, patients should be closely monitored for adverse reactions. Drugs causing QT prolongation.

Permitted Therapy

The following therapies are permitted on the study:

- Premedication with antiemetic agents, suggested agents include a 5-T-HT3 blocker in combination with another antiemetic or corticosteroid approximately 30 minutes prior to patritumab deruxtecan infusion. Choice of agents is based on investigator's discretion as per local/institutional guidelines.
- Standard therapies for pre-existing medical conditions, medical and/or surgical complications. if they are not included in the list of prohibited medications. Any medication intended solely for supportive care (e.g., analgesics, antidiarrheals, anti-depressants) may also be used at the investigator's discretion.
- Antiemetics: Metoclopramide standard antiemetic products may be used as the first drug of choice. If a 5HT3 inhibitor needs to be incorporated because the Metoclopramide is insufficient, the use of Granisetron 1 mg/12h will be permitted due to the low capacity for prolonging QTc with respect to Ondansetron.
- Bisphosphonates for the prevention of skeletal events.
- Concomitant use of drugs that are substrates of OAT1 and OATP1B1 is allowed. Because the exposure of MAAA-1181a is expected to be low with U3-1402 at doses administered in clinical studies, the inhibition of OAT1 and OATP1B1 by MAAA-1181a is expected to have a little impact on drugs that are substrates of OAT1 and OATP1B1.

- Concomitant use of drugs that inhibit MATE2-K, P-gp, Breast Cancer Resistance Protein (BCRP), and MRP1 is allowed, although patients should be closely monitored for adverse reactions. Because the urinary excretion of MAAA-1181a is expected to be low, MATE2-K (which is involved in the excretion of substrates into urine) is expected to have a minimal impact on the exposure of MAAA-1181a.
- Multiple efflux transporters such as P-gp and BCRP are involved in the excretion of MAAA-1181a; therefore, the risk of interactions with these inhibitors is also expected to be low. Likewise, because the expression of MRP1 in the liver is low, 16 the inhibition of MRP1 is expected to have a little impact.

The above lists of medications are not necessarily comprehensive. Thus, the investigator should contact the Medical Monitor if questions arise regarding medications not listed above.

8. Study Assessments

8.1 Table of assessments

Please see the Table of assessments (Appendix A) for the study procedures performed during the study.

8.2 Screening

The ICF must be signed by the patient before any procedure specific to the study is performed. However, procedures conducted as part of the subject's routine clinical management (e.g., diagnostic imaging studies) obtained prior to signing the ICF may be utilized for screening or baseline purposes provided these procedures are conducted as specified in the protocol and within the protocol-defined timeframes. After signed the ICF, patients will be registered and assigned a study number in the electronic clinical report form (e-CRF). Signature of the ICF can occur outside the 42 days timeframe.

The following assessments and procedures must be completed and reviewed to confirm that patients meet all eligibility criteria before initiation of study treatment. Screening and pretreatment tests and evaluations will be performed within 42 days, unless otherwise specified.

- Complete anamnesis with medical history and demographic data
- Complete physical examination
- Breast and axillar examination
- Vital signs (blood pressure, pulse rate and temperature) and weight
- Height
- Clinical TNM classification
- ECOG performance status (see Appendix C)
- 12-lead ECG
- LVEF determination by ECHO or MUGA scan
- Breast and axillary ultrasound
- ER, PgR, and HER2 status as per most recent ASCO- CAP guideline in the target breast lesion as per most recent American Society of Clinical Oncology (ASCO)-College of American Pathologists Guideline (CAP) guideline, including the percentage of ER positive and PgR positive cells. Ki67 (percentage) will also be collected
- Tumor sample collection: Available tumor FFPE samples from baseline biopsy
- Determination of ERBB3 mRNA expression by central lab
- Hemogram, fasting serum chemistry and coagulation
- Pregnancy test (if applicable). A positive urine test must be confirmed with a serum test
- Current and concomitant medications (including supplements, prescription and over-the-counter medications, and phytotherapy / homeopathy, will be recorded)

- Baseline signs and symptoms, according to the NCI Common Terminology for Classification of Adverse Events (CTCAE) v.5.0
- Documentation of concomitant medications
- **Medical monitor validation should be obtained prior to initiating the study treatment**

8.3 C1D1

On Day 1, the following assessments will be performed if have not been made during the 7 days prior to the treatment assignment. All the assessments should be done before the drug administration. All the visit data will be registered in the e-CRF:

- Vital signs (blood pressure, pulse rate and temperature) and weight
- ECOG performance status (see Appendix C)
- Breast and axillar examination
- Hemogram, fasting serum chemistry and coagulation (The C1D1 blood samples can be extracted during screening if it is done up to 7 days before C1D1)
- Pregnancy test (if applicable). A positive urine test must be confirmed with a serum test
- Blood samples for biomarkers (The C1D1 blood samples can be extracted during screening if it is done up to 7 days before C1D1 and after the medical monitor has validated the inclusion of the patient)
- Collection of any AEs and SAEs, with assignment of the appropriate AE grade according to NCI CTCAE v.5.0
- Documentation of concomitant medications

8.4 C1D3-D7

The optional tissue samples and blood sample will be taken between day 3 and day 10.

- Blood samples for biomarkers are mandatory regardless of patients acceptance of optional biopsy)
- An optional biopsy will be optional between day 3 and day 10 days after the dose of U3-1402.

8.5 C1D21

A biopsy will be mandatory 21 (±3) days after the dose of U3-1402. During this visit, the following assessments will be performed:

- Vital signs (blood pressure, pulse rate and temperature) and weight
- Breast & axillar examination
- Breast and axillary ultrasound
- ECOG performance status (see Appendix C)
- Hemogram and fasting serum biochemistry
- Blood samples for biomarkers

- Collection of any AEs and SAEs, with assignment of the appropriate AE grade according to NCI CTCAE v.5.0
- Documentation of concomitant medications

All the visit data will be registered in the e-CRF.

8.6 End of Study (EOS) / Safety follow-up (SFV) visit

On Day 49 (±14), approximately 28 days after the second biopsy the following assessments will be performed:

- Complete physical examination
- Vital signs (blood pressure, pulse rate and temperature) and weight
- ECOG performance status (see Appendix C)
- Hemogram and fasting serum biochemistry
- Collection of any AEs and SAEs, with assignment of the appropriate AE grade according to NCI CTCAE v.5.0
- Documentation of concomitant medications
- NAC and/or adjuvant treatment
- Surgery outcome (including pathological staging)
- Blood samples for biomarkers

All the visit data will be registered in the e-CRF.

Neo/Adjuvant endocrine therapy and/or chemotherapy or surgical intervention will be delivered as per investigator’s choice. Post-operative radiotherapy must be administered according to local guidelines.

8.7 10-Months Follow-up data collection

After ten (10) months (±60 days),

- NAC and/or adjuvant treatment response
- Surgery outcome if performed after EOS/SFV visit

All the data will be registered in the e-CRF.

8.8 Description of Study Assessments

The Trial Flow Chart – **Appendix A** – summarizes the trial procedures to be performed at each visit. Individual trial procedures are described in detail below. It may be necessary to perform these procedures at unscheduled time points if deemed clinically necessary by the investigator.

Furthermore, additional evaluations/testing may be deemed necessary by SOLTI for reasons related to participant safety. In some cases, such evaluation/testing may be potentially sensitive in nature (e.g., HIV, Hepatitis C, etc.), and thus local regulations may require that additional

informed consent be obtained from the participant. In these cases, such evaluations/testing will be performed in accordance with those regulations.

8.8.1 Informed Consent

At the screening visit, the investigator or authorized delegate will explain the study to the patient, answer all his/her questions and obtain the patient's written informed consent before performing any study-related procedure. The ICF will be signed in duplicate and the patient and/or the authorized representative obtain one original of the signed ICF. The second original is filed with the study documents at the investigational site.

8.8.2 Medical History and Demographic data

Complete anamnesis should be done including: demographic data (age, sex and race or ethnic group), reproductive status, clinically significant disease in the last 3 years, surgeries, toxic background (smoking, alcohol or drug abuse), cancer history (including treatments and previous procedures against cancer), history of breast cancer with tumor characteristics (date of diagnosis, treatments and antineoplastic procedures, type and histological grade, estrogen and progesterone receptor status, HER2 status and percentage of Ki67 expression) and complete cardiovascular history.

All medication taken during the 7 days prior to screening, and as scheduled in table of assessments, including supplements, prescription and over-the-counter medications, and phytotherapy / homeopathy, will be recorded.

8.8.3 Inclusion/Exclusion criteria

The inclusion and exclusion criteria will be reviewed by medically qualified study personnel to ensure that the patient qualifies for the study. The review of inclusion and exclusion criteria and confirmation of the patient's eligibility will be noted in patient's source documents and appropriate e-CRF.

8.8.4 Physical examinations

A complete physical examination should include evaluation of the head, mouth, eyes, ears, nose and throat, neck (including thyroid), abdomen, back, extremities, lymph nodes, and cardiovascular systems, dermatological, locomotor, respiratory, digestive, genitourinary and neurological systems, weight (Kg) and height (cm). If no abnormal data are not observed during selection, the full physical examination may be based on concurrent signs and / or symptoms.

8.8.5 Clinical Breast examination

Assessment of primary breast tumor and regional lymph nodes must be done by physical examination (palpation) at Screening (If this determination has been made within 7 days prior to treatment assignation, it will not be necessary to repeat it on day 1) and during the study as stated in the Table of assessments. Breast tumor measurement by gauge or ruler will be performed preferably by the same evaluator throughout the study and recorded in the e-CRF.

8.8.6 Vital signs and weight

Vital signs will include measurements of heart rate; systolic and diastolic blood pressure while the patient is in a seated position after resting for at least 5 minutes; and oral or axillary temperature.

8.8.7 Tumor assessment: TNM classification

Tumor staging will include a breast ultrasound. If the ultrasound cannot be performed or the tumor cannot be visualized in it, MRI can be used alternatively. Baseline distant sites tumor staging procedures should be performed at the discretion of the treating investigator.

Lymph node stage will be determined by physical axillary exploration and/or by ultrasound. For patients with suspicious axillary lymph nodes, axillary staging should include fine needle aspiration biopsy or core needle biopsy guided by ultrasound performed within 42 days prior to the start of treatment.

8.8.8 Breast and axillar ultrasound.

Ultrasound of the breast performed before the study inclusion is accepted in the 42 days prior to the start of treatment.

8.8.9 Assessment of cardiac function

All patients must have an LVEF determination of no less than 50% by echocardiogram or MUGA, at most 42 days prior to enrollment. Investigators must be aware of the local institution regulations' regarding the maximum permissible repetition frequency of MUGA scans. Patients must also be assessed for history of cardiac events, physical exam, and a baseline ECG prior to enrollment to exclude any cardiac condition that makes them ineligible to participate in this trial.

ECG recordings must be performed after the patient has been resting in a supine position for at least 10 minutes. All ECGs are to be obtained prior to other procedures scheduled at that same time (e.g., vital sign measurements, blood draws).

8.8.10 Biological sample collection.

- Screening tumor biopsy: Tumor sample from primary tumor is mandatory. Available pre-treatment FFPE core needle biopsy evaluable for PAM50 and ERBB3 mRNA expression (2 tumor cylinders with a minimal tissue surface of 10 mm^2 tissue, containing at least 30% tumor cells and having enough tissue to do at least one 3 µm slide, nine 4 µm slides, and eighteen 10 µm slides.). A window longer than 42 days is allowed. If archival tissue is either insufficient or unavailable, a new biopsy from the pretreatment tumor must be obtained. The biopsy will only be done if the tumor is larger or equal than 1 cm in its greatest diameter by ultrasound, otherwise the patient will not be eligible. **Tumor tissue samples should be submitted in the form of paraffin blocks.**
- Core biopsy must be obtained at C1D21 after treatment administration (three cores - two in paraffin block and one 5 mg of flash frozen tissue mg tissue). Optionally, for those patients who accepted, a tissue sample at C1D3-D7 will be obtained (two cores – one in paraffin block and one 5 mg of flash frozen tissue) only if the tumor is larger or equal to 2 cm, and the patient provided consent,
- Blood samples for biomarkers: In all cases, peripheral blood will be collected aseptically by venipuncture. Blood samples for ctDNA (30 ml) will be obtained at baseline, C1D21 and EOS. Blood samples for HER3 mRNA (10 ml) will be obtained at baseline, C1D3-D7 and C1D21. While blood samples for DXd (5 ml) will be collected after dosing at C1D3-D7 and C1D21.

8.8.11 Laboratory tests

- Hemogram with formula and platelet count includes a count of erythrocytes, hemoglobin, hematocrit, leukocyte count with formula (neutrophils, eosinophils, basophils, lymphocytes, monocytes and other cells) and platelet count.
- Fasting serum biochemistry (≥ 8 hours fasting): sodium, potassium, calcium, glucose, creatinine, total protein, albumin, total bilirubin, direct bilirubin if total bilirubin is > ULN, alkaline phosphatase, LDH, AST, ALT and GGTP.
- Coagulation panel, including INR and aPTT.
- Pregnancy test: A urine or serum β-HCG test must be performed for women of childbearing potential (including pre-menopausal women who have had a tubal ligation). If the urine test is positive or cannot be confirmed as negative, a serum pregnancy test will be required. For all other women, documentation must be present in medical history

confirming that the patient is not of childbearing potential. Testing should be performed within 7 days prior to the first administration of study medication.

8.9 Patient, treatment and study discontinuation

8.9.1 Patient Discontinuation

Patients have the right to voluntarily withdraw from the study at any time for any reason. In addition, the Investigator has the right to withdraw a patient from the study at any time. Reasons for withdrawal from the study may include but are not limited to the following:

- Patient withdrawal of consent at any time
- Any medical condition that the Investigator or Sponsor determines may jeopardize the patient's safety if she/he continues in the study.
- Investigator or Sponsor determines it is in the best interest of the patient.
- Patient non-compliance.

Every effort should be made to obtain information on patients who withdraw from the study for any reason. The primary reason for withdrawal from the study should be documented on the appropriate e-CRF. However, patients will not be followed for any reason after consent has been withdrawn.

8.9.2 Study treatment discontinuation

Experimental treatment will be delivered until any of the following occur:

- Completion of study treatment.
- Patient's consent withdrawal.
- Presence of other medical conditions that prohibit continuation of therapy.
- Failure of the patient to comply with study procedures that compromise safety, despite repeated efforts of the Investigator to contact the patient, with complete documentation of the circumstances.
- Unacceptable toxicity.
- Presence of new medical information that warrants the termination of the study.
- Termination of the study by the Sponsor.

Patients who complete all study treatment will undergo surgery or primary medical treatment (e.g. neoadjuvant chemotherapy) at the discretion of the treating physician according to local practices.

Patients who withdraw consent will go off the study. No further study procedures will be performed, nor will they be considered for the endpoint analysis. Every effort should be made to obtain information on participants who withdraw from the study. The primary reason for

withdrawal from the study should be documented on the appropriate e-CRF. However, participants will not be followed for any reason after consent has been withdrawn.

If clinical and/or radiological PD is observed at any time or the patient shown intolerable toxicity, experimental treatment will be withheld, and surgery or further treatment will be administered at the discretion of the Investigator in the best interest of the patient.

8.9.3 Early study discontinuation

SOLTI, as Sponsor, may decide to terminate the study at any time for safety reasons, due to slow patient recruitment or due to the emergence of new data that could alter the relevance of the study objectives. A steering committee (SC) managed by SOLTI will provide their counsel. Ethics committees and health authorities will be informed about all decisions in this respect. The decision to terminate the study early will be binding for all investigators at all study sites.

In the event of early termination, all investigators must inform their patients as soon as possible and they will be responsible for the follow-up and subsequent therapeutic management as appropriate and in accordance with recommended standard practice. Investigators may be informed of additional procedures to be performed to ensure the safety and better care of their patients.

9. Evaluation criteria

In this study we evaluate if a single dose of U3-1402 increases the CelTIL score in women with early breast cancer. Moreover, we want to identify biological changes as secondary and exploratory endpoints.

9.1 Biological response assessment

The biological response to the study treatment will be assessed in a central laboratory by measuring changes of CelTIL score in all the patients in each cohort.

CelTIL at D15 was found to be associated with pCR both as a continuous variable and as group categories using a pre-defined cutoff. In this study, we evaluate the CelTIL score increase at C1D21 days of treatment.

CelTIL score = −0.8 × tumor cellularity (in %) + 1.3 × TILs (in %). The minimum and maximum unscaled CelTIL scores will be −80 and 130. This unscaled CelTIL score will then be scaled to reflect a range from 0 to 100 points.

CelTIL after C1D21 will be compared to pretreatment CelTIL.

Biological changes, as defined by gene expression between post-treatment and pretreatment samples following U3-1402 will be identified. These changes will be evaluated by gene expression of a panel of 60 breast cancer-related genes, including PAM50, and a panel of 770 immune-related genes.

Although only one dose of the study drug will be administered, safety and tolerability will be evaluated in terms of incidence, duration and severity of AEs assessed by the NCI Common Terminology for Classification of Adverse Events (CTCAE) version 5.0 including AEs resulting in delay or discontinuation of study treatment.

9.2 **Research blood samples**

All participants will be asked to donate the blood samples as indicated in the table of assessments (Appendix A) for protocol driven as well as for future ethically approved research.

Further details on sample processing, handling and shipment are provided in the laboratory study manual.

10. Translational research

Translational research data will be collected outside the clinical database; correlations with relevant clinical features will be performed by specialized scientists of the PPD PPD Lab PPD All the data will be reported in a separate ad-hoc report, which will integrate the results and findings described in the clinical study report.

All the data analyses of the study will be under the responsibility of SOLTI and they will be based on a statistical plan according to the protocol specifications.

10.1 Biological specimens

On-study biopsies can provide valuable information regarding target engagement and downstream pathway modulation. Assessing the effects of this combination in this previously untreated patient population provides a unique opportunity for characterizing the effects of this novel combination and the patient population which might benefit the most from it. In fact, the integration of pre- and post-treatment biomarkers is expected to improve prognostic algorithms and help identify patients for whom a specific neoadjuvant therapy is appropriate.

The tissue and blood samples collected will be used to identify biomarkers that may be predictive of response or toxicity to the proposed treatments and/or prognostic for breast cancer. Since the knowledge of new markers that may correlate with disease activity and the efficacy or safety of the treatment is evolving, the analyses may change during the study and may include the determination of additional markers of tumorigenesis pathways and mechanisms of treatment response. The collected tumor tissue and blood samples may also be used to develop and validate diagnostic assays and allow the generation of statistically meaningful biomarker data.

Remaining sample materials after the completion of the initial biomarker assessments (e.g., aliquots of tumor RNA or DNA) may be used for further assessment of expanded marker panels.

Samples will be stored at the PPD Lab PPD for up CCI [redacted]

All patients will consent to the collection and use of research blood and tissue samples. All samples will be linked anonymized and only identified by the trial ID and unique sample number allocated by the SOLTI SAMPLE TRACKER SYSTEM (STS) coordinating team.

Instruction manuals outlining sampling procedures, storage conditions, shipment instructions and supply kits will be provided for the central laboratory assessments and the recruiting sites.

For more information about the sampling procedures and shipment see instructions in the appropriate sections of the Sample Management Guide (SMG).

10.1.1 Tumor tissue samples

Collection of tumor biopsies is an essential part of this study. Paraffin-embedded tumor samples will be obtained from all patients. Quality of tumor samples, even for screening samples, will be evaluated upon arrival at the central laboratory. Pathological analysis includes hematoxylin and eosin (H&E) staining, identification of areas with greater amounts of tumor cells and determination of their tumor cell percentage. Confirmation of eligibility criteria requires central assessment of an adequate quantity and quality of screening sample. If not available, the patient must agree to re-biopsy.

- Tumor samples will also be obtained and analyzed for study endpoints: after treatment with U3-1402 with a core-biopsy prior to neoadjuvant therapy or surgery.
- Optionally, if a tumor is larger or equal to 2 cm, and the patient provided consent, a tissue sample at C1D3-D7 will be obtained.

Specimens will be used for CelTIL and biomarker assessment. It is likely that not all assays will be performed on samples provided by each patient (possibly because of insufficient tumor material or inadequate sample quality). Therefore, assays will be performed with the following priority to ensure a high likelihood of generating data to support the objectives of the study, including but not limited to H&E stains, Ki67 staining, celTIL determination. In addition, other exploratory assessments, including but not limited to genetic analyses, mutational tumor load and gene-expression may be evaluated.

In case of multifocality/multicentricity, samples pre, during and post-treatment must be from the same lesion.

If a tumor block cannot be obtained for various reasons (e.g., the tumor tissue is not sufficient at core-biopsy), the site should discuss with SOLTI.

Details of handling, shipping, sample preparation and conservation are described in the Sample Management Guide (SMG).

10.1.2 Collection of blood samples

Collection of blood samples is an essential part of this study. Samples will be handled accordingly with the objective to which attempt to address. Please refer to point 7.6.10 Biological sample collection and o Table of assessments to further details.

Details of handling, shipping, samples preparation and conservation are described in the SMG

10.2 Gene expression signatures

Gene expression of the PAM50 genes and >500 additional genes, including ERBB3, will be analyzed. The following signatures or biological processes will be evaluated, among others: PAM50 genes, ROR score, EGFR-related genes (ERBB2, EGFR, ERBB3, ERBB4, GRB7, CRYAB), PI3K pathway genes (PIK3CA, PTEN), Claudin-low subtype, VEFG signature, Proliferation, Estrogen-responsive signature, Basal-luminal differentiation, DNA replication genes, Stroma-related genes.

10.2.1 The NanoString assay

The NanoString nCounter Analysis System (http://www.nanostring.com/) delivers direct, multiplexed measurements of gene expression through digital readouts of the relative abundance of hundreds of mRNA transcripts. It uses gene-specific probe pairs that hybridize directly to the mRNA sample in solution eliminating any enzymatic reactions that might introduce bias in the results. After hybridization, all the sample processing steps are automatized.

10.2.2 RNA extraction

The NanoString-based test will use RNA from FFPE breast tumor tissue. A section of the FFPE breast tissue (biopsy will first be examined with hematoxylin and eosin (H&E) staining to determine percent tumor nuclei, percent normal nuclei, and percent necrosis per standard pathology processing. The slide will then be reviewed by a pathologist, who will identify and mark the region of the tissue that contains an adequate percentage of tumor for the gene expression test on the slide. RNA will be extracted from the FFPE tissue sample using an RNA isolation kit. Additional general-purpose laboratory reagents are required for deparaffinization. The extraction process includes a step for removing genomic DNA from the sample. Following extraction of total RNA and removal of genomic DNA, the optical density is measured at wavelengths of 260 nm and 280 nm to determine both yield and purity using a low volume spectrophotometer. RNA will be stored at –80 ºC until the time of testing.

10.2.3 Technical procedures and data analysis

For each set of up to 10 RNA samples isolated from BC tissue, the user will pipette a defined amount of RNA into separate tubes within a 12-reaction strip tube and add the CodeSet and hybridization buffer as specified within the assay protocol. A set concentration of reference sample is pipetted into the remaining two tubes with CodeSet and hybridization buffer. The CodeSet consists of probes for each gene that is targeted, additional probes for endogenous “housekeeping” normalization genes and positive and negative controls that are spiked into the assay. The reference sample consists of in vitro transcribed RNA for the targeted genes and housekeeping genes. Once the hybridization reagents are added to the respective tubes, the user

transfers the strip tube into a specified heated-lid heat block and incubates for a defined period at a set temperature of 65 ºC.

Upon completing hybridization, the user will then transfer the strip tube containing the set of 10 assays and two reference samples into the nCounter Prep Station. An automatized purification process then removes excess capture and reporter probe through two successive hybridization-driven magnetic bead capture steps. The nCounter Prep Station then transfers the purified target/probe complexes into an nCounter cartridge for capture to a glass slide. Following completion of the run, the user removes the cartridge from the Prep Station and seals it with an adhesive film.

The cartridge is then sealed and inserted into the nCounter Digital Analyzer. The analyzer counts the number of probes captured on the slide for each gene, which corresponds to the amount of target in solution. The signals of each sample will be normalized using the housekeeping genes to control for input sample quality. The signals are then normalized to the reference sample within each run to control for run-to-run variations. The resulting normalized data is input into the BC-subtyping algorithm or gene signatures score algorithm

10.3 Genetic analysis

DNA mutation analysis will be performed on tumor DNA samples to identify specific loci, genes, or gene pathways associated with sensitivity to the treatment combination in order to search for predictive biomarkers of response.

Evaluation of tumor mutational load by Next Generation Sequencing will be also performed to assess the predictive value of the most prevalent mutations in this breast cancer subtype DNA-seq of the most prevalent mutations including, but not limited to: TP53, ESR1, PIK3CA, or, HER2.

10.4 Collection of plasma samples

There is increasing evidence that circulating DNA obtained from blood specimens of patients with cancer is representative of the DNA and mutational status of tumor cells. Plasma samples will be collected at three time points during the study (day 1 of treatment, day 21 (±3) after treatment administration and at EOS). 30ml peripheral blood will be collected aseptically by venipuncture into sterile EDTA-treated tubes for future genomic analyses. Those samples will be kept in the biorepository for further analysis. At this point, no platform for the analysis of plasma including but not limited to ctDNA, analysis has been selected. This analysis will be considered future research and will be done out of this protocol.

10.5 Sample storage and destruction

Any blood or tumor samples collected according to the Schedule of Assessments can be analyzed for any of the tests outlined in the protocol.

All residual samples (or leftover biologic samples after protocol-defined studies are completed) obtained during the study (FFPE, fresh-frozen, plasma, etc.) will be stored in an academic central repository. The repository will be the PPD PPD Lab PPD The repository will be under the CCI [redacted]

All biomarker specimens will be retained for new research related to this study and/or disease in accordance with the recommendations and approval of the Study Steering Committee. Samples will be destroyed only if required by local laws relating to the collection, storage, and destruction of biological specimens.

CCI [redacted]

Results may not be available until the end of the study. Results of biomarker development or other exploratory studies are not placed in the subject's medical record and are not to be made available to the subject, members of the family, the personal physician, or other third parties, except as specified in the informed consent.

The subject retains the right to request that the sample material be destroyed by contacting the Investigator. Following the request from the subject, the Investigator is to provide the sponsor with the required study and subject number so that any remaining blood or tumor samples and any other components from the cells can be located and destroyed. Samples will be destroyed once all protocol-defined procedures are completed. However, information collected from samples prior to the request for destruction will be retained by SOLTI.

The Sponsor is the exclusive owner of any data, discoveries, or derivative materials from the sample materials and is responsible for the destruction of the sample(s) at the request of the subject through the Investigator, at the end of the storage period, or as appropriate (e.g., the scientific rationale for experimentation with a certain sample type no longer justifies keeping the sample).

11. Assessment of safety, monitoring and reporting

11.1 Monitoring, recording and reporting of adverse events

11.1.1 Time Period and Reporting Timelines for AEs/SAEs

All AEs/SAEs will be collected and documented on appropriate e-CRF(s) from the time the subject signs informed consent until end of study visit.

Medical occurrences that begin before the start of study intervention but after obtaining informed consent will be recorded on the Medical History/Current Medical Conditions section of the e-CRF not the AE section.

Investigators are not obligated to actively seek AE or SAE after conclusion of the study participation. However, if the Investigator learns of any SAE, at any time after termination of the subject from the study, and he/she considers the event to be reasonably related to the study intervention or study participation, the Investigator must promptly notify the Sponsor.

If an AE is considered serious, both the AE e-CRF and the SAE Report Form must be completed.

The SAE Report Form must be submitted by the Investigator or his/her designee to the Pharmacovigilance responsible within 24 hours of awareness, via email:

PPD

The Investigator will submit any clinically significant updated SAE data to the Pharmacovigilance responsible within 24 hours of awareness.

Adverse Event of Special Interest (AESI) for this study should be documented on the relevant e-CRFs and flagged as AESI, within 24 hours of awareness. The AESIs should also be submitted following the SAE process.

Copies of the documents (e.g. medical records, death certificate) submitted to the Sponsor will be identified with the patient number, all personal data will not be included.

AEs should be collected from the signature of ICF until end of study visit.

11.1.2 AE Definition

According to ICH guidelines for Good Clinical Practice an AE is any harmful, unintended, or untoward medical occurrence that may appear or worsen in a subject during study course. It may be a new intercurrent illness, a worsening concomitant illness, an injury, or any concomitant impairment of the subject's health, including laboratory test values (as specified by the criteria below), regardless of etiology. Any worsening (i.e., any clinically significant adverse change in the frequency or intensity of a pre-existing condition) should be considered an AE. A diagnosis or syndrome should be recorded on the AE e-CRF rather than the individual signs or symptoms of the diagnosis or syndrome.

For this study, disease progression while on treatment or disease recurrence/relapse after the EoS visit are not to be reported as AE. Unless the cause of death is unknown, death per se is not an AE but may be the outcome of an AE.

An overdose, accidental or intentional, whether it is associated with an AE, or abuse, withdrawal, sensitivity or toxicity to an investigational product should be reported as an AE. If an overdose is associated with an AE, the overdose and adverse event should be reported as separate terms.

11.1.3 Follow-up of AEs and SAEs

The Investigator is responsible for following up AEs that are serious, considered related to the study intervention or study procedures, or that caused the participant to discontinue from the study. These events will be followed until resolution, stabilization, the event is otherwise explained, or the participant is lost to follow-up.

11.2 Evaluation of adverse event

All subjects will be monitored for AEs during the study. Assessments may include monitoring of any or all the following parameters: the subject's clinical symptoms, laboratory, pathological, radiological or surgical findings, physical examination findings, or other appropriate tests and procedures. The Investigator must perform an assessment of seriousness, severity, and causality of the AE according to NCI CTCAE criteria.

The Investigator will evaluate all adverse events as to:

11.2.1 Seriousness

A serious adverse event (SAE) is any AE occurring at any dose that:

- Results in death
- Is life-threatening (i.e., in the opinion of the Investigator, the subject is at immediate risk of death from the AE)
- Requires inpatient hospitalization or prolongation of existing hospitalization (hospitalization is defined as an inpatient admission, regardless of length of stay)
- Results in persistent or significant disability/incapacity (a substantial disruption of the subject's ability to conduct normal life functions)
- Is a congenital anomaly/birth defect
- Constitutes an important medical event

Important medical events are defined as those occurrences that may not be immediately life threatening or result in death, hospitalization, or disability, but may jeopardize the subject or require medical or surgical intervention to prevent one of the other outcomes listed above.

Medical and scientific judgment should be exercised in deciding whether such an AE should be considered serious.

Events **not considered** to be SAEs are hospitalizations for:

- A standard procedure for protocol therapy administration. However, hospitalization or prolonged hospitalization for a complication of therapy administration will be reported as an SAE.
- Routine treatment or monitoring of the studied indication is not associated with any deterioration in condition.
- The administration of blood or platelet transfusion as routine treatment of studied indication. However, hospitalization or prolonged hospitalization for a complication of such transfusion constitutes an SAE.
- A procedure for protocol/disease-related investigations (e.g., surgery, scans, endoscopy, sampling for laboratory tests, bone marrow sampling). However, hospitalization or prolonged hospitalization for a complication of such procedures constitutes an SAE.
- Hospitalization or prolongation of hospitalization for technical, practical, or social reasons, in absence of an AE.
- A procedure that is planned (i.e., planned prior to starting of treatment on study); must be documented in the source document and the e-CRF. Hospitalization or prolonged hospitalization for a complication constitutes an SAE.
- An elective treatment of a pre-existing condition unrelated to the studied indication.
- Emergency outpatient treatment or observation that does not result in admission, unless fulfilling other seriousness criteria above.

11.2.2 Severity / intensity

For all AEs/ SAEs, the Investigator must assess the severity / intensity of the event. The severity / intensity of AEs will be graded according to NCI CTCAE, Version 5.0:

http://ctep.cancer.gov/protocolDevelopment/electronic_applications/ctc.htm#ctc_40

AEs that are not defined in the NCI CTCAE should be evaluated for severity / intensity according to the following scale:

- Grade 1 = Mild – transient or mild discomfort; no limitation in activity; no medical intervention/therapy required
- Grade 2 = Moderate – mild to moderate limitation in activity, some assistance may be needed; no or minimal medical intervention/therapy required
- Grade 3 = Severe – marked limitation in activity, some assistance usually required; medical intervention/therapy required, hospitalization is possible
- Grade 4 =Life threatening– extreme limitation in activity, significant assistance required; significant medical intervention/therapy required, hospitalization or hospice care probable
- Grade 5 =Death - the event results in death

The term "severe" is often used to describe the intensity of a specific event (as in mild, moderate or severe myocardial infarction); the event itself, however, may be of relatively minor medical significance (such as severe headache). This criterion is not the same as "serious" which is based on subject/event outcome or action criteria associated with events that pose a threat to a subject's life or functioning.

Seriousness, not severity, serves as a guide for defining regulatory obligations.

11.2.3 Causality

The Investigator must determine the relationship between the administration of protocol drug(s) and the occurrence of an AE /SAE as "Not related" or "Related", as defined below:

- Not related: The temporal relationship of the adverse event to protocol drug(s) delivered administration makes **a causal relationship unlikely or remote**, or other medications, therapeutic interventions, or underlying conditions provide a sufficient explanation for the observed event.
- Related: The temporal relationship of the adverse event to protocol drug(s) delivered administration makes **a causal relationship possible**, and other medications, therapeutic interventions, or underlying conditions do not provide a sufficient explanation for the observed event.

There may be situations in which an SAE has occurred, and the Investigator has minimal information to include in the initial report. However, it is very important that the Investigator always provide an assessment of causality for every event before the initial submission of the SAE data. The SAE reporting should not be delayed obtaining enough information.

11.2.4 Duration

For both AEs/SAEs, the Investigator will record the start and stop dates of the event.

11.2.5 Action taken

The Investigator will report the action taken with IMP(s) as a result of an AE/SAE, as applicable (e.g., discontinuation or reduction of IMP) and report if concomitant and/or additional treatments were given for the event.

11.2.6 Outcome

The Investigator will report the outcome of all AEs/ SAEs. All SAEs that have not resolved upon discontinuation of the subject's participation in the study must be followed until resolution.

11.3 Abnormal laboratory values

An abnormal laboratory value is an AE if the abnormality:

- results in discontinuation from the study;
- requires treatment, modification/interruption of IMP dose, or any other therapeutic intervention; or
- is judged to be of significant clinical importance.

If a laboratory abnormality is one component of a diagnosis or syndrome, then only the diagnosis or syndrome should be recorded on the AE page/screen of the e-CRF. If the abnormality was not a part of a diagnosis or syndrome, then the laboratory abnormality should be recorded as the AE.

11.4 Reporting of adverse events of special interest (Study Specific AESIs)

There are certain types of AE that are considered of study specific importance (AESI). These AEs (serious and non-serious) must be documented in an expedited manner (24 hours after learning of the event) on the relevant e-CRFs as AESI and reported as SAE.

A list of these events is included in Appendix D.

11.5 Pregnancy

Pregnancies and suspected pregnancies (including a positive pregnancy test regardless of age) of a female subject occurring after the first exposure to the study drug(s) will be reported to the Sponsor and Pharmacovigilance responsible within 24 hours of awareness, using the Pregnancy Report Form. Protocol drug(s) delivered is to be discontinued immediately.

The Pregnancy Report Form must be submitted by the Investigator or his/her designee to the Pharmacovigilance responsible within 24 hours of awareness, via email:

PPD

The Investigator will follow the female subject until completion of the pregnancy and must notify the Sponsor immediately about the outcome of the pregnancy (either normal or abnormal outcome).

All neonatal deaths that occur within 28 days of birth should be reported, without regard to causality, as SAEs. Any infant death after 28 days that the Investigator suspects is related to the in-utero exposure to the protocol drug(s) should also be reported within 24 hours of the Investigator's knowledge of the event using the SAE Report Form or approved equivalent form. In addition, any congenital anomaly/birth defect in a child born to a female patient exposed to study

treatment should be classified as a serious adverse event, recorded on the Adverse Event e-CRF, and reported to the Sponsor immediately (i.e., no more than 24 hours after learning of the event).

11.6 Expedited reporting of adverse events to Regulatory Authorities and the Ethics Committee

SOLTI the Sponsor, will inform relevant Regulatory Authorities and SOLTI will inform the applicable Ethic Committees of:

- all relevant information about serious unexpected adverse events suspected to be related to the IMP that are fatal or life-threatening as soon as possible, and in any case no later than seven calendar days after knowledge by the Sponsor. Relevant follow-up information for these cases will subsequently be submitted within an additional eight calendar days.
- all other serious unexpected events suspected to be related to the IMP as soon as possible, but within fifteen calendar days of first knowledge by the by the Sponsor.

12. Statistical considerations

12.1 Statistical and analytic plan

This is a window of opportunity exploratory study to evaluate the effect of U3-1402 in patients with treatment naïve early breast cancer.

The statistical analysis will be conducted following the principles as specified in International Conference on Harmonization (ICH) Topic E9 (CPMP/ICH/363/96). The significance level will be α=0.05 2-sided for all tests. As an exploratory study, multiple testing without adjustment of the significance level is considered acceptable.

A summary of the general approach to statistical analysis is given below. A detailed statistical plan will be issued and approved prior to declaration of clean database lock. Eligibility exceptions and protocol deviations will be summarized by cohort. Patient disposition will be tabulated by cohort, and reasons for premature discontinuation will be summarized in tables and as a CONSORT flow chart.

12.1.1 Analysis populations

All patients included into the study will be considered in the statistical report.

Intent-to-Treat (ITT) efficacy population includes all patients that are enrolled in the study

Safety population (SP) includes the set of patients who received the treatment. This population will be analyzed for the secondary endpoint of safety.

Protocol Population (PP): All efficacy analyses will be performed on a Per-Protocol Population. The Per-Protocol population is defined as the population of all enrolled patients who received the study drug and for whom tumor biopsy specimens at baseline and at C1D21 after treatment administration are available for assessment of biological response. The per-protocol population also excludes patients who had a major violation of protocol.

12.1.2 Statistical analysis

The main objective of this proof of concept study is to estimate the biological effect of a single dose of U3-1402 treatment in a window opportunity study before surgical procedure in a patient with treatment naïve breast cancer. The study is designed to formally assess the mean change in CelTIL score between baseline and C1D21 after one dose of U3-1402 (part A). However, no formal comparison between cohorts will be done and statistical analyses will be performed for estimations of proportions or means (or medians) for all variables by confidence intervals

calculations. A specific statistical analysis plan (SAP) will be performed before the formal close of the database.

12.1.3 Planned analysis

A first safety-only interim analysis will be performed after the first 10 patients have been treated and have available safety data; decisions on study continuation will be made by the Study Steering Committee. Safety and efficacy interim analysis will be performed after the first 30 patients are available for central CELTIL evaluation during Part A and decisions on study continuation will be made by the Study Steering Committee. As the safety of the treatment has been evaluated in the metastatic setting, no interruption in the accrual will be done, to maintain the dynamic of accrual in the trial

Part B will be performed sequentially, depending on the risk/benefit assessment of therapy in patients included in Part B.

12.1.4 Analysis methods

The CelTIL score will be calculated following the formula: [CelTIL score = −0.8 × tumor cellularity (in %) + 1.3 × TILs (in %)]. The minimum and maximum unscaled CelTIL scores will be −80 and 130. This unscaled CelTIL score will then be scaled to reflect a range from 0 to 100 points.

For the primary analysis, the study will evaluate the change in CelTIL score between baseline and C1D21, in paired samples after one dose of U3-1402. On one hand, if the null hypothesis of normal distribution in CelTIL score change values is not rejected (Shapiro test; p-value> 0.05). To evaluate the change in CelTIL score in paired samples (baseline and C1D21), the mean change in CelTIL score and the standard deviation will be reported. The paired sample t-test will be used to calculate if the mean change in CelTIL score is statistically different from zero. The significance level will be $\alpha = 0.05$ using a two-sided test. On the other hand, if the null hypothesis of normal distribution of change values of CelTIL score is rejected (Shapiro test; p-value< 0.05). To evaluate the change in CelTIL score in paired samples (baseline and C1D21), the median change in CelTIL score and the interquartile range (IQR) will be reported. The non-parametric Wilcoxon signed-rank test will be used to calculated if the median change in CelTIL score is statistically different from zero. The significance level will be $\alpha = 0.05$ using a two-sided test.

This analysis will be performed using PP approach. Moreover, the ITT set will be also used as a sensitivity analysis to assess the robustness of the results with the same approach.

For secondary endpoints, the change in CelTIL score will be estimated in subpopulations (e.g., i) ERRB3 cohort, ii) intrinsic subtype, etc.). However, no formal statistical analysis will be carried out because the trial is not adequately powered to detect difference between them.

Correlations will be estimated using the Pearson correlation coefficient. If the null hypothesis of normal distribution is rejected, correlations will be estimated using the Spearman's rank correlation coefficient.

Secondary and exploratory efficacy analysis will be performed using PP approach. The ITT set will be also used as a sensitivity analysis to assess the robustness of the results with the same approach.

Overall, categorical variables will be described by the number and percentage of patients. Missing observations will be tabulated as a separate category. Two percentage columns are presented, total percentage (total %) and valid percentage (valid %), that is, the percentage over the sum of valid responses plus missing values and the percentage over the total of valid responses. When there are not missing values, both of them are equal and then only one will be shown. Continuous variables will be described by the number of non-missing observations, number of missing observations, mean, standard deviation, median, interquartile range (25th and 75th percentiles [Q1 and Q3, respectively]) and extreme values (minimum and maximum).

Mean, median, standard deviation, confidence intervals, minimum and maximum values will be presented using one decimal place more than the recorded data. The number of patients will be presented as a whole number. P-values will be presented to 3 decimal places (or as <0.001 where appropriate). Percentage values will be printed with one digit to the right of decimal point (e.g., 43.2%, 5.7%, etc.).

Final methods to be used will be described in the SAP.

12.2 Sample size determination

Part A of the study would require a sample size of 72 (number of pairs) to achieve a power of 80% and a level of significance of 5% (two-sided), for detecting a mean of the differences of 13 between pairs, assuming the standard deviation of the differences to be 38.6, which is the standard deviation observed in 403 patients with CelTIL data across 4 SOLTI trials[86–89,95]

Assuming a 10% drop-out or lack of tissue, a sample size of 80 patients will be target to be recruited.

In Part A, a target sample size of 80 patients with HR+/HER2-negative disease will be included. To ensure the representation of the entire range of ERBB3 expression, ERBB3 expression will be determined prospectively (≤7 working days) and approximately 20 patients will be included in each one of the 4 groups based on ERBB3 quartile expression (ultra-low, low, medium and high).

The distribution of the patients will be:

Cohorts	ERBB3 central expression	N
Cohort 1	Ultra-low ERBB3 expression	~20
Cohort 2	Low ERBB3 expression	~20
Cohort 3	Medium ERBB3 expression	~20
Cohort 4	High ERBB3 expression	~20

Part B of the study will target to include 20 patients with HR+/HER2-negative breast cancer and 15 patients with TNBC treated with a dose of 5.6 mg/kg. No formal sample size calculation has been made, but it will maintain a reasonable proportion with Part A, also allowing recruitment within a reasonable time frame. ERBB3 expression will be determined retrospectively.

13. Direct access to source data / documents

To ensure compliance with the Clinical Trial Protocol, GCP, and applicable regulatory requirements, the Investigator should permit auditing by or on the behalf of SOLTI and by regulatory authorities.

The Investigator agrees to allow the auditors/inspectors to have direct access to his/her study records for review, being understood that these personnel are bound by professional secrecy, and as such, will not disclose any personal identity or personal medical information.

The Investigator will make every effort to help with the performance of the audits and inspections, giving access to all necessary facilities, data, and documents. As soon as the Investigator is notified of a planned inspection by the authorities, he/she will inform SOLTI and authorize SOLTI to participate in this inspection. The confidentiality of the data verified and the protection of the patients, should be respected during these inspections. Any results and information arising from the inspections by the regulatory authorities will be immediately communicated by the Investigator to SOLTI.

The Investigator must take appropriate measures required by the SOLTI to take corrective actions for all problems found during the audit or inspections.

13.1 Study monitoring

13.1.1 Responsibilities of the investigators

The Investigators and delegated Investigator staff undertake to perform the Clinical Trial in accordance with this Clinical Trial Protocol, ICH guidelines for GCP, and the applicable regulatory requirements.

The Investigator is required to ensure compliance with all procedures required by the Clinical Trial Protocol and with all study procedures provided by the Sponsor (including security rules). The Investigator agrees to provide reliable data and all information requested by the Clinical Trial Protocol (with the help of the e-CRF, Discrepancy Resolution Form [DRF], or other appropriate instrument) in an accurate and legible manner according to the instructions provided and to ensure direct access to source documents by SOLTI representatives.

If any circuit includes transfer of data, attention should be paid to the confidentiality of the patient's data to be transferred.

The Investigator may appoint other individuals, as he/she may deem appropriate, as Sub-investigators to assist in the conduct of the Clinical Trial in accordance with the Clinical Trial Protocol. All Sub-investigators must be appointed and listed in a timely manner. The Sub-investigators will be supervised by and work under the responsibility of the Investigator. The Investigator will provide them with a copy of the Clinical Trial Protocol and all necessary information.

13.1.2 Responsibilities of the sponsor and monitoring

The Sponsor of this Clinical Trial, SOLTI, is responsible to Health Authorities for taking all reasonable steps to ensure the proper conduct of the Clinical Trial Protocol about ethics, Clinical Trial Protocol compliance, and integrity and validity of the data recorded on the e-CRFs. Thus, the main duty of the Monitoring Team is to help the Investigator and SOLTI maintain a high level of ethical, scientific, technical, and regulatory quality in all aspects of the Clinical Trial.

At regular intervals during the Clinical Trial the site will be contacted through monitoring visits, letters, or telephone calls by a representative of the Monitoring Team to review study progress, Investigator and patient compliance with Clinical Trial Protocol requirements, and any emergent problems.

The Monitor will visit the study site on a regular basis to ensure that the study is conducted and documented in accordance with this protocol, ICH GCP guidelines, regulatory requirements, and any study specific documents such as e-CRF completion guidelines.

Monitoring visits will be conducted to confirm that:

- The investigational team is adhering to the study protocol.
- IC has been obtained from all participants.
- AEs have been reported as required.
- Data are being accurately recorded on the e-CRFs.
- IMP is being stored correctly and drug accountability is being performed on an on-going basis.
- Facilities are, and remain, acceptable throughout the study.
- The Investigator and the site are receiving enough information and support throughout the study.

Moreover, during monitoring visits, the data recorded on the e-CRFs, source documents, and other study-related records will be compared against each other in order to ensure accurate data that reflect the actual existence of the subject in the study, i.e., source data verification.

13.1.3 Source documents requirements

According to the ICH guidelines for GCP, the Monitoring Team must check the e-CRF entries against the source documents, except for the pre-identified source data directly recorded on the e-CRF. The IC Form will include a statement by which the patient allows SOLTI´s duly authorized personnel, the Ethics Committee (IRB/IEC), and the regulatory authorities to have direct access to original medical records, which support the data on the e-CRFs (e.g., patient's medical file, appointment books, original laboratory records, etc.).

These personnel, bound by professional secrecy, must maintain the confidentiality of all personal identity or personal medical information (according to confidentiality and personal data protection rules).

13.1.4 Use and completion of case report forms and additional requests

It is the responsibility of the Investigator to maintain adequate and accurate Case Report Forms, which for this trial will be of electronic nature. The electronic Case Report Form (e-CRF) is designed by SOLTI and/or its designee to record according to SOLTI instructions, all observations and other data pertinent to the Study. All e-CRFs should be completed in their entirety in a neat, legible manner to ensure accurate interpretation of data. Should a correction be made, the corrected information will be entered in the e-CRF overwriting the initial information. An audit trail allows identifying the modification.

Data are available to SOLTI as soon as they are entered in the e-CRF system. The computerized handling of the data by the Sponsor when available in the e-CRF may generate additional requests (DRF) to which the Investigator is obliged to respond by confirming or modifying the data questioned. The requests along with their responses will be managed through the e-CRF.

13.1.5 Use of computerized systems

Procedures shall be employed, and control designed to ensure the confidentiality of electronic records. Such procedures and controls must include validation of systems to ensure accuracy and reliability, ability to generate accurate and complete copies of records, protection of records to enable retrieval, use of secure, computer-generated, time-stamped entries, use of operational system checks, use of device checks to determine validity of source data input, determination that those who develop, maintain, or use such systems have adequate education and training, the establishment and adherence of written policies to deter record falsification, the use of appropriate controls over systems documentation including the distribution or use of documentation for system operation and maintenance, and revision and change control procedures, which document time-sequenced development and modifications of systems documentation. For data management activities, the e-CRF will be built using e-Clinical SQL Server.

13.2 Data Management

Data management and handling will be conducted according to the study specific Data Management Plan in accordance with ICH guidelines and standard operating procedures (SOPs), which will be prepared and approved before the end of the experimental phase of the study.

Data entry, validation, and data queries will be handled by the data will be subjected to validation according to SOPs in order to ensure accuracy in the collected e-CRF data.

Before database closure, reconciliation will be performed between the SAEs entered in the safety database and the study database.

Any deviations, i.e., discrepancies and additions from the process defined in the Data Management Plan, will be described in a study-specific Data Management Report.

13.3 Confidentiality

13.3.1 Patient records

The Investigator shall ensure that the anonymity of the patients and protection of her identity from unauthorized individuals are maintained. In the eCRFs or other documents sent to the data management department, patients shall not be identified by name, but by an identification code. The Investigator must keep a patient inclusion log with their codes and full names. The Investigator will have to store the documents that are not going to be sent to the data processing center, for example, original patient ICFs, in a strictly confidential manner.

13.3.2 Study documentation and related data

All information disclosed or provided by SOLTI (or any company/institution acting on its behalf), or produced during the Clinical Trial, including, but not limited to, the Clinical Trial Protocol, the e-CRFs, the Summary of Product Characteristics and the results obtained during the course of the Clinical Trial, is confidential prior to the publication of the Clinical Trial results. The Investigator and any person under his/her authority agree to undertake to keep confidential and not to disclose the information to any third party without the prior written approval of SOLTI. However, the submission of this Clinical Trial Protocol and other necessary documentation to the Ethics Committee (IRB/IEC) is expressly permitted, the IRB/IEC members having the same obligation of confidentiality.

The Sub-investigators are bound by the same obligations as the Investigator. The Investigator must inform the Sub-investigators of the confidential nature of the Clinical Trial. The Investigator and the Sub-investigators should use the information solely for the purposes of the Clinical Trial, to the exclusion of any use for their own or for a third party's account. Furthermore, the

Investigator and SOLTI agree to adhere to the principles of personal data confidentiality in relation to the patients, the Investigator, and the collaborators involved in the study.

14. Quality control and quality assurance

14.1 Protocol deviations

The investigator should document and explain any protocol deviations. The investigator should promptly report any deviations that might have an impact on patient safety and data integrity to the Sponsor and to the IRB/EC in accordance with established IRB/EC policies and procedures.

Deviations to the study protocol will be documented in a Protocol Deviation Log.

The classification of subjects into protocol violators will be made during a meeting before database lock. Listings will indicate the allocation of subjects by analysis set and the number of subjects per analysis set will be recorded in the Clinical Study Report (CSR).

14.2 Insurance

SOLTI, the Sponsor, should provide insurance or should indemnify (legal and financial coverage) the Investigator/the institution against claims arising from the study, except for claims that arise from malpractice, negligence, or non-compliance with the protocol.

14.3 Study committees

14.3.1 Steering committee (SC)

A SC will be in place during the study. It will be created comprising SOLTI investigators associated with the design or conduct of the study, as well as non-SOLTI investigators, provided the Principal Investigator of the study deems it appropriate. They will review the final protocol and the overall data at the time of primary analysis.

The SC shall ensure that the management of the study is carried out in line with the Protocol and GCP guidelines. The SC may propose and must review and approve any necessary amendment to the Protocol. It will also make decisions regarding the publications generated from the study data.

Information on members, responsibilities and frequency of steering committee meetings are specified in the SC statutes.

The SC may be consulted for advice when needed, either in face-to-face meetings or via teleconference.

Details of SC operating procedures including meeting frequency are provided in the SC Charter.

14.4 Premature discontinuation of the study or close-out of a site

Decided by Daiichi-Sankyo and SOLTI (Sponsor), in the following cases:

- If new information on the product leads to doubt as to the benefit/risk ratio.
- If the Investigator has received from the Sponsor all IP, means, and information necessary to perform the Clinical Trial and has not included any patient after a reasonable period mutually agreed upon.
- In the event of breach by the Investigator of a fundamental obligation under this agreement, including, but not limited to, breach of the Clinical Trial Protocol, breach of the applicable laws and regulations, or breach of the ICH guidelines for GCP.
- If the total number of patients are included earlier than expected.

In any case, the Sponsor will notify the Investigator of its decision by written notice.

Decided by the Investigator:

The Investigator must notify (30 days' prior notice) the Sponsor of his/her decision and give the reason in writing. In all cases (decided by the Sponsor or by the Investigator), the appropriate Ethics Committee(s) (IRB/IEC) and Health Authorities should be informed according to applicable regulatory requirements.

15. Ethics

15.1 Independent Ethics Committee

This protocol and any amendments will be submitted to a properly constituted Independent Ethics Committee (IEC), in accordance with the International Conference on Harmonization (ICH) guidelines, the applicable European Directives and local legal requirements, for approval of the study. Approval must be obtained in writing before the first subject can be recruited.

During the Clinical Trial, any amendment or modification to the Clinical Trial Protocol should be submitted to the Ethics Committee (IRB/IEC) before implementation, unless the change is necessary to eliminate an immediate hazard to the patients, in which case the IRB/IEC should be informed as soon as possible. It should also be informed of any event likely to affect the safety of patients or the continued conduct of the Clinical Trial, any change in safety. The Summary of Product Characteristics will be sent to the Ethics Committee (IRB/IEC).

Principal Investigator will not be released at the study site and the Investigator will not start the study before the written and dated approval/favorable opinion is received by the Investigator and SOLTI. Before study start, the Investigator must sign the Protocol signature page to confirm that he/she agrees to conduct the study in compliance with these documents and with all instructions and procedures described in the Protocol and to permit access to all relevant data and records to the study monitors, Daiichi Sankyo/SOLTI auditors, representatives of Daiichi-Sankyo/SOLTI's clinical quality assurance department, designated Daiichi Sankyo/SOLTI agents, IECs and health authorities upon request.

A progress report is sent to the Ethics Committee (IRB/IEC) at least annually in the DSUR and a summary of the Clinical Trial’s outcome at the end of the Clinical Trial.

15.2 **Ethical conduct of the study**

This Clinical Trial will be conducted in compliance with the Protocol, regulatory requirements, the ICH guidelines for Good Clinical Practice (GCP) and the ethical principles of the latest revision of the Declaration of Helsinki as adopted by the World Medical Association.

This Clinical Trial will be recorded in the public registry website clinicaltrials.gov and Spanish registry of clinical trials (*Registro Español de Ensayos Clinicos*) before the enrollment of the first

patient. The registry will contain basic information about the trial, understandable enough to inform patients and their healthcare practitioners on how to enroll in the trial.

15.3 Informed Consent

All potential participants will receive verbal and written information on the study in a previous interview with the study doctor in their hospital. In this information, special emphasis will be placed on the fact that participation in the study is voluntary and that the patient may withdraw herself from the study at any time and for any reason, without this affecting her medical care. All patients will have the opportunity to ask questions about the study and they will be given enough time to decide if they wish to participate.

The ICF must mention the specific data that will be recorded, collected, processed and that can be sent to countries pertaining to and outside of the European Economic Area (EEA). In accordance with the Regulation (EU) 2016/679 of the European Parliament and of the Council of 27 April 2016 and LOPD, the individuals participating in the study shall not be identified.

Personal data will be managed in accordance with the applicable legislation in force at the time, and in particular, in accordance with Regulation (EU) No 2016/679 of 27 April 2016 on the protection of individuals with regard to the processing of their personal data (hereinafter, “GDPR”).

The patient will be given a copy of the Patient Information Sheet, including the signed ICF.

Eligible patients can only be included after granting their written informed consent (before witnesses, when required by laws or standards). The signature of the ICF must be obtained before performing any study-specific procedures (that is, any of the procedures described in the Protocol). The date on which the ICF is signed must be recorded in the eCRF.

The investigators will be given an ICF approved by the IEC that is appropriate for the study and that satisfies the ICH GCP standards and legal requirements. Any modification to this ICF proposed by the Investigator must be accepted by Daiichi Sankyo/SOLTI and approved by the local IEC. A copy of the approved version must be provided to the trial monitor after IEC approval is obtained.

Patients must be re-consented to the most current version of the Consent Forms (or to a significant new information/findings addendum in accordance with applicable laws and EC policy) during their participation in the study.

An optional Informed Consent Form that addresses the use of remaining samples for future exploratory research will be provided to each site. The investigator or authorized designee will explain to each patient the objectives of the exploratory research. Patients will be told that they are free to refuse to participate and may withdraw their specimens at any time and for any reason during the storage period. A signature will be required to document a patient's agreement to allow any remaining specimens to be used for exploratory research. Patients who decline to participate will not sign the optional ICF.

15.4 Clinical trial / protocol amendments

All appendices attached hereto and referred to herein are made part of this Clinical Trial Protocol.

The Investigator should not implement any deviation from, or changes to the Clinical Trial Protocol without agreement by SOLTI and prior review and documented approval/favorable opinion from the IRB/IEC of an amendment, except where necessary to eliminate immediate hazard(s) to patients enrolled in the trial, or when the change(s) involves only logistical or administrative aspects of the trial. Any change agreed upon will be recorded in writing, the written amendment will be signed by the Investigator and SOLTI, and the signed amendment will be filed with this Clinical Trial Protocol. Daiichi Sankyo will be informed about any protocol amendment and requires Daiichi Sankyo approval for substantial amendments.

Any amendment to the Clinical Trial Protocol requires written approval/favorable opinion by the Ethics Committee (IRB/IEC) prior to its implementation, unless there are overriding safety reasons.

In some instances, an amendment may require a change to the IC Form. The Investigator must receive an IRB/IEC approval/favorable opinion concerning the revised IC form prior to implementation of the change and patient signature should be recollected if necessary.

16. Data handling and record keeping

16.1 Subject records and source data

A current copy of the Curriculum Vitae describing the experience, qualification, and training of each Investigator and Sub-Investigator will be signed, dated and provided to the Sponsor SOLTI prior to the beginning of the Clinical Trial.

It is the responsibility of the Investigator to record essential information in the medical records in accordance with national regulations and requirements. The following information should be included as a minimum:

- A statement that the subject is in a clinical study
- The identity of the study, e.g., Study code
- Subject screening number and/or subject number
- That IC was obtained and the date
- Diagnosis
- Dates of all visits during the study period
- Any information relating to AEs
- All treatments and medications prescribed/administered (including dosage)
- Date of study termination
- Subject health service identification number

The Investigator is responsible for ensuring the accuracy, completeness, legibility, and timeliness of the data recorded on the e-CRFs. Data reported on the e-CRF that are derived from source documents should be consistent with the source documents or the discrepancies should be explained. Locked sections of CRFs will be monitored on a regular basis.

16.2 **Study documentation and storage of records**

The Investigator or the site must store the essential documents (as defined in Standard E6 of the ICH GCP, section 8) as required by the applicable administrative requirements. The Investigator or the site will have to take measures to prevent the accidental or early destruction of said documents.

After the study is closed, the Investigator or the designated individual from the site must store all study records in a secure, protected area of the site, except where, according to local legislation,

they must be stored by another person or institution. The records must be stored to enable their easy, timely recovery as needed (for example, audit or inspection) and, whenever possible, to allow any subsequent analysis of the data together with the site's assessment, the support systems and the personnel. When so permitted by local standards or legislation or the institution's policy, some or all these records can be stored in a format other than hard copy (for example, microfile, scanned or electronic support); however, precaution must be exercised before adopting these measures. The Investigator must make certain that all reproductions are legible, a true and exact copy of the original and that they comply with the standards for accessibility and recovery, including that of re-generating a printed copy if necessary. Moreover, the Investigator must make certain that there is an acceptable backup copy of these reproductions and an acceptable quality control process for making said reproductions.

[redacted]

The Investigator must notify SOLTI of any change in the availability of the files, for example: archived in an off-site facility or transfer of the ownership of the records in the event the Investigator leaves the site.

16.3 Property rights

All information supplied by SOLTI in connection with this study will remain the sole property of the Sponsor and is to be considered confidential information. No confidential information will be disclosed to others without obtaining prior written consent from the Sponsor and will not be used except in the performance of this Study. SOLTI will retain ownership of all data.

All information, documents, and intellectual property (IP) provided by Daiichi Sankyo are and remain the sole property of them. The Investigator shall not mention any information or the Product in any application for a patent or for any other intellectual property rights.

In terms of the results generated by the study, SOLTI will maintain ownership of all data and will allow Daiichi Sankyo to make scientific use of it, if it considers it pertinent. Any reports, documents, publications and inventions directly or indirectly arising from this study shall be the immediate and exclusive property of the Sponsor. SOLTI, as Sponsor, may use and exploit all the study results at its full discretion.

As the case may be, the Investigator and/or the Sub-investigators should provide all assistance required by SOLTI, at SOLTI's expense, for obtaining and defending any patent, including the signature of legal documents.

17. Publication policy

All publications and presentations of the study results must comply with the approved scientific practice and academic standards and comply with SOLTI's publication policy.

This policy is available to all Investigators and groups participating in the study. Every Investigator who wishes to publish or present the study data must obtain the permission of the study SC. Daiichi Sankyo must review and approve any article prior to it being submitted to journals, congresses or conferences. The authorship of the publications will be decided by the SC, which will follow the standard guidelines of peer-reviewed journals in line with International Committee of Medical Journal Editors authorship requirements. and will observe SOLTI's publication policy.

Key design elements of this protocol will be posted in a publicly accessible database such as clinicaltrials.gov prior to enrolment of the first patient.

Once the study has been completed, SOLTI will prepare a Clinical Study Report (CSR) in line with the ICH guidelines on the structure and content of clinical study reports (ICH E3). All publications and presentations must be based on the CSR.

SOLTI agrees to communicate the results of the study, regardless of the outcome of the trial, in public access media, and shall particularly respect the dissemination of the results in scientific publications, assuming an active role in the preparation of articles or summaries, in line with the SC, and participating in its submission to the corresponding authors. All study communications must mention Daiichi Sankyo's economic support and supply of the drugs. In order to guarantee the protection of the intellectual property arising from the study, Daiichi Sankyo will have the right to review all articles prior to submission.

SOLTI will have the right to use the results in internal presentations and for the external promotion of its interests.

If an Investigator wishes to publish results from this clinical study, written permission to publish must be obtained from SOLTI in advance.

18. References

1. Sung H, Ferlay J, Siegel RL, Laversanne M, Soerjomataram I, Jemal A, et al. Global Cancer Statistics 2020: GLOBOCAN Estimates of Incidence and Mortality Worldwide for 36 Cancers in 185 Countries. CA Cancer J Clin. 2021;71:209–49.

2. Common Cancer Sites - Cancer Stat Facts [Internet]. SEER. [cited 2022 Feb 14]. Available from: https://seer.cancer.gov/statfacts/html/common.html

3. Siegel RL, Miller KD, Jemal A. Cancer Statistics, 2017. CA Cancer J Clin. United States; 2017;67:7–30.

4. Gonzalez-Angulo AM, Morales-Vasquez F, Hortobagyi GN. Overview of Resistance to Systemic Therapy in Patients with Breast Cancer. In: Yu D, Hung M-C, editors. Breast Cancer Chemosensitivity. Springer New York; p. 1–22.

5. Swain SM, Baselga J, Kim S-B, Ro J, Semiglazov V, Campone M, et al. Pertuzumab, Trastuzumab, and Docetaxel in HER2-Positive Metastatic Breast Cancer. N Engl J Med. 2015;

6. Ciruelos E, Cortes-Funes H, Ghanem I, Manso L, Arteaga C. Role of inhibitors of mammalian target of rapamycin in the treatment of luminal breast cancer. Anticancer Drugs. England; 2013;24:769–80.

7. Ciruelos E, Pascual T, Arroyo Vozmediano ML, Blanco M, Manso L, Parrilla L, et al. The therapeutic role of fulvestrant in the management of patients with hormone receptor-positive breast cancer. 23:201–8.

8. Fribbens C, O'Leary B, Kilburn L, Hrebien S, Garcia-Murillas I, Beaney M, et al. Plasma ESR1 Mutations and the Treatment of Estrogen Receptor-Positive Advanced Breast Cancer. J Clin Oncol Off J Am Soc Clin Oncol. United States; 2016;34:2961–8.

9. Coates AS, Winer EP, Goldhirsch A, Gelber RD, Gnant M, Piccart-Gebhart M, et al. Tailoring therapies--improving the management of early breast cancer: St Gallen International Expert Consensus on the Primary Therapy of Early Breast Cancer 2015. Ann Oncol Off J Eur Soc Med Oncol. England; 2015;26:1533–46.

10. Prat A, Cheang MCU, Martin M, Parker JS, Carrasco E, Caballero R, et al. Prognostic significance of progesterone receptor-positive tumor cells within immunohistochemically defined luminal A breast cancer. J Clin Oncol Off J Am Soc Clin Oncol. United States; 2013;31:203–9.

11. Martin M, Rodriguez-Lescure A, Ruiz A, Alba E, Calvo L, Ruiz-Borrego M, et al. Randomized phase 3 trial of fluorouracil, epirubicin, and cyclophosphamide alone or followed by Paclitaxel for early breast cancer. J Natl Cancer Inst. United States; 2008;100:805–14.

12. Martín M, Prat A, Rodríguez-Lescure Á, Caballero R, Ebbert MTW, Munárriz B, et al. {PAM}50 proliferation score as a predictor of weekly paclitaxel benefit in breast cancer. 138:457–66.

13. Chia S, Piccort M, Gradishar W. Fulvestrant vs exemestane following non-steroidal aromatase inhibitor failure: first overall survival data from the EFECT trial. BREAST CANCER Res Treat. SPRINGER 233 SPRING STREET, NEW YORK, NY 10013 USA; 2007. p. S115–6.

14. Johnston SRD, Kilburn LS, Ellis P, Dodwell D, Cameron D, Hayward L, et al. Fulvestrant plus anastrozole or placebo versus exemestane alone after progression on non-steroidal aromatase inhibitors in postmenopausal patients with hormone-receptor-positive locally advanced or metastatic breast cancer ({SoFEA}): a composite, multicen. 14:989–98.

15. de Azambuja E, Holmes AP, Piccart-Gebhart M, Holmes E, Di Cosimo S, Swaby RF, et al. Lapatinib with trastuzumab for HER2-positive early breast cancer (NeoALTTO): survival outcomes of a randomised, open-label, multicentre, phase 3 trial and their association with pathological complete response. Lancet Oncol. England; 2014;15:1137–46.

16. Johnston SRD. Enhancing the Efficacy of Hormonal Agents with Selected Targeted Agents. Clin Breast Cancer. Elsevier; 2009;9:S28–36.

17. Prat A, Perou CM. Deconstructing the molecular portraits of breast cancer. 5:5–23.

18. Prat A, Adamo B, Cheang MCU, Anders CK, Carey LA, Perou CM. Molecular characterization of basal-like and non-basal-like triple-negative breast cancer. The oncologist. United States; 2013;18:123–33.

19. Prat A, Carey LA, Adamo B, Vidal M, Tabernero J, Cortés J, et al. Molecular Features and Survival Outcomes of the Intrinsic Subtypes Within {HER}2-Positive Breast Cancer. 106.

20. Prat A, Bianchini G, Thomas M, Belousov A, Cheang MCU, Koehler A, et al. Research-based PAM50 subtype predictor identifies higher responses and improved survival outcomes in HER2-positive breast cancer in the NOAH study. Clin Cancer Res Off J Am Assoc Cancer Res. United States; 2014;20:511–21.

21. Gianni L, Pienkowski T, Im Y-H, Tseng L-M, Liu M-C, Lluch A, et al. 5-year analysis of neoadjuvant pertuzumab and trastuzumab in patients with locally advanced, inflammatory, or early-stage HER2-positive breast cancer (NeoSphere): a multicentre, open-label, phase 2 randomised trial. Lancet Oncol. England; 2016;17:791–800.

22. Swain SM, Kim SB, Cortés J, Ro J, Semiglazov V, Campone M, et al. Pertuzumab, trastuzumab, and docetaxel for HER2-positive metastatic breast cancer (CLEOPATRA study): Overall survival results from a randomised, double-blind, placebo-controlled, phase 3 study. Lancet Oncol. 2013;

23. Marous M, Bièche I, Paoletti X, Alt M, Razak ARA, Stathis A, et al. Designs of preoperative biomarkers trials in oncology: a systematic review of the literature. Ann Oncol. 2015;26:2419–28.

24. Glimelius B, Lahn M. Window-of-opportunity trials to evaluate clinical activity of new molecular entities in oncology. Ann Oncol Off J Eur Soc Med Oncol. England; 2011;22:1717–25.

25. Schmitz S, Duhoux F, Machiels J-P. Window of opportunity studies: Do they fulfil our expectations? Cancer Treat Rev. Netherlands; 2016;43:50–7.

26. Koboldt DC, Fulton RS, McLellan MD, Schmidt H, Kalicki-Veizer J, McMichael JF, et al. Comprehensive molecular portraits of human breast tumours. Nature. 2012;

27. Prat A, Parker JS, Karginova O, Fan C, Livasy C, Herschkowitz JI, et al. Phenotypic and molecular characterization of the claudin-low intrinsic subtype of breast cancer. 12:R68.

28. Perou CM, Sorlie T, Eisen MB, van de Rijn M, Jeffrey SS, Rees CA, et al. Molecular portraits of human breast tumours. Nature. England; 2000;406:747–52.

29. Millikan RC, Newman B, Tse C-K, Moorman PG, Conway K, Smith L V, et al. Epidemiology of basal-like breast cancer. 109:123–39.

30. Anderson WF, Rosenberg PS, Prat A, Perou CM, Sherman ME. How many etiological subtypes of breast cancer: two, three, four, or more? J Natl Cancer Inst. United States; 2014;106.

31. Bastien RRL, Rodríguez-Lescure Á, Ebbert MTW, Prat A, Munárriz B, Rowe L, et al. PAM50 Breast Cancer Subtyping by RT-qPCR and Concordance with Standard Clinical Molecular Markers. BMC Med Genomics. 2012;5:44.

32. Nielsen TO, Parker JS, Leung S, Voduc D, Ebbert M, Vickery T, et al. A comparison of PAM50 intrinsic subtyping with immunohistochemistry and clinical prognostic factors in tamoxifen-treated estrogen receptor-positive breast cancer. Clin Cancer Res Off J Am Assoc Cancer Res. United States; 2010;16:5222–32.

33. Usary J, Zhao W, Darr D, Roberts PJ, Liu M, Balletta L, et al. Predicting drug responsiveness in human cancers using genetically engineered mice. Clin Cancer Res Off J Am Assoc Cancer Res. United States; 2013;19:4889–99.

34. Cheang MCU, Martin M, Nielsen TO, Prat A, Rodriguez-Lescure A, Ruiz A, et al. Quantitative hormone receptors, triple-negative breast cancer (TNBC), and molecular subtypes: A collaborative effort of the BIG-NCI NABCG. J Clin Oncol. 2012;30:1008.

35. Parker JS, Mullins M, Cheang MCU, Leung S, Voduc D, Vickery T, et al. Supervised risk predictor of breast cancer based on intrinsic subtypes. J Clin Oncol Off J Am Soc Clin Oncol. United States; 2009;27:1160–7.

36. Prat A, Parker JS, Fan C, Perou CM. PAM50 assay and the three-gene model for identifying the major and clinically relevant molecular subtypes of breast cancer. Breast Cancer Res Treat. 2012;135:301–6.

37. Yarden Y, Sliwkowski MX. Untangling the ErbB signalling network. Nat Rev Mol Cell Biol. England; 2001;2:127–37.

38. Mishra R, Alanazi S, Yuan L, Solomon T, Thaker TM, Jura N, et al. Activating HER3 mutations in breast cancer. Oncotarget. Impact Journals LLC; 2018;9:27773–88.

39. Breuleux M. Role of heregulin in human cancer. Cell Mol Life Sci CMLS. Switzerland; 2007;64:2358–77.

40. Khurana A, Gonzalez-Guerrico A, Lupu R. Heregulin in breast cancer: old story, new paradigm. Curr Pharm Des. Bentham Science Publishers; 2014;20:4874–8.

41. Desmedt C, Zoppoli G, Gundem G, Pruneri G, Larsimont D, Fornili M, et al. Genomic characterization of primary invasive lobular breast cancer. J Clin Oncol. American Society of Clinical Oncology; 2016;34:1872–81.

42. Lee-Hoeflich ST, Crocker L, Yao E, Pham T, Munroe X, Hoeflich KP, et al. A central role for HER3 in HER2-amplified breast cancer: implications for targeted therapy. Cancer Res. AACR; 2008;68:5878–87.

43. Vaught DB, Stanford JC, Young C, Hicks DJ, Wheeler F, Rinehart C, et al. HER3 is required for HER2-induced preneoplastic changes to the breast epithelium and tumor formation. Cancer Res. AACR; 2012;72:2672–82.

44. Vlacich G, Coffey RJ. Resistance to EGFR-targeted therapy: a family affair. Cancer Cell. United States; 2011;20:423–5.

45. Kruser TJ, Wheeler DL. Mechanisms of resistance to HER family targeting antibodies. Exp Cell Res. United States; 2010;316:1083–100.

46. Baselga J, Cortes J, Im S-A, Clark E, Ross G, Kiermaier A, et al. Biomarker analyses in CLEOPATRA: a phase III, placebo-controlled study of pertuzumab in human epidermal growth factor receptor 2-positive, first-line metastatic breast cancer. J Clin Oncol Off J Am Soc Clin Oncol. United States; 2014;32:3753–61.

47. Zhang S, Huang W-C, Li P, Guo H, Poh S-B, Brady SW, et al. Combating trastuzumab resistance by targeting SRC, a common node downstream of multiple resistance pathways. Nat Med. United States; 2011;17:461–9.

48. Agus DB, Akita RW, Fox WD, Lewis GD, Higgins B, Pisacane PI, et al. Targeting ligand-activated ErbB2 signaling inhibits breast and prostate tumor growth. Cancer Cell. United States; 2002;2:127–37.

49. de Alava E, Ocana A, Abad M, Montero JC, Esparis-Ogando A, Rodriguez CA, et al. Neuregulin expression modulates clinical response to trastuzumab in patients with metastatic breast cancer. J Clin Oncol Off J Am Soc Clin Oncol. United States; 2007;25:2656–63.

50. Xia W, Petricoin EF 3rd, Zhao S, Liu L, Osada T, Cheng Q, et al. An heregulin-EGFR-HER3 autocrine signaling axis can mediate acquired lapatinib resistance in HER2+ breast cancer models. Breast Cancer Res BCR. England; 2013;15:R85.

51. Newby JC, Johnston SR, Smith IE, Dowsett M. Expression of epidermal growth factor receptor and c-erbB2 during the development of tamoxifen resistance in human breast cancer. Clin Cancer Res. AACR; 1997;3:1643–51.

52. Shou J, Massarweh S, Osborne CK, Wakeling AE, Ali S, Weiss H, et al. Mechanisms of tamoxifen resistance: increased estrogen receptor-HER2/neu cross-talk in ER/HER2-positive breast cancer. J Natl Cancer Inst. United States; 2004;96:926–35.

53. Tovey S, Dunne B, Witton CJ, Forsyth A, Cooke TG, Bartlett JMS. Can molecular markers predict when to implement treatment with aromatase inhibitors in invasive breast cancer? Clin Cancer Res Off J Am Assoc Cancer Res. United States; 2005;11:4835–42.

54. Hutcheson IR, Goddard L, Barrow D, McClelland RA, Francies HE, Knowlden JM, et al. Fulvestrant-induced expression of ErbB3 and ErbB4 receptors sensitizes oestrogen receptor-positive breast cancer cells to heregulin beta1. Breast Cancer Res BCR. England; 2011;13:R29.

55. Frogne T, Benjaminsen R V, Sonne-Hansen K, Sorensen BS, Nexo E, Laenkholm A-V, et al. Activation of ErbB3, EGFR and Erk is essential for growth of human breast cancer cell lines with acquired resistance to fulvestrant. Breast Cancer Res Treat. Netherlands; 2009;114:263–75.

56. Bieche I, Onody P, Tozlu S, Driouch K, Vidaud M, Lidereau R. Prognostic value of ERBB family mRNA expression in breast carcinomas. Int J Cancer. United States; 2003;106:758–65.

57. Esteva FJ, Hortobagyi GN, Sahin AA, Smith TL, Chin DM, Liang SY, et al. Expression of erbB/HER receptors, heregulin and P38 in primary breast cancer using quantitative immunohistochemistry. Pathol Oncol Res POR. Netherlands; 2001;7:171–7.

58. Bae SY, La Choi Y, Kim S, Kim M, Kim J, Jung SP, et al. HER3 status by immunohistochemistry is correlated with poor prognosis in hormone receptor-negative breast cancer patients. Breast Cancer Res Treat. Netherlands; 2013;139:741–50.

59. Lipton A, Goodman L, Leitzel K, Cook J, Sperinde J, Haddad M, et al. HER3, p95HER2, and HER2 protein expression levels define multiple subtypes of HER2-positive metastatic breast cancer. Breast Cancer Res Treat. Netherlands; 2013;141:43–53.

60. Fujiwara S, Ibusuki M, Yamamoto S, Yamamoto Y, Iwase H. Association of ErbB1-4 expression in invasive breast cancer with clinicopathological characteristics and prognosis. Breast Cancer Tokyo Jpn. Japan; 2014;21:472–81.

61. Morrison MM, Hutchinson K, Williams MM, Stanford JC, Balko JM, Young C, et al. ErbB3 downregulation enhances luminal breast tumor response to antiestrogens. J Clin Invest. United States; 2013;123:4329–43.

62. Balko JM, Miller TW, Morrison MM, Hutchinson K, Young C, Rinehart C, et al. The receptor tyrosine kinase ErbB3 maintains the balance between luminal and basal breast epithelium. Proc Natl Acad Sci U S A. United States; 2012;109:221–6.

63. Prat A, Navarro A, Pare L, Reguart N, Galvan P, Pascual T, et al. Immune-Related Gene Expression Profiling After PD-1 Blockade in Non-Small Cell Lung Carcinoma, Head and Neck Squamous Cell Carcinoma, and Melanoma. Cancer Res. United States; 2017;77:3540–50.

64. Pare L, Pascual T, Segui E, Teixido C, Gonzalez-Cao M, Galvan P, et al. Association between PD1 mRNA and response to anti-PD1 monotherapy across multiple cancer types. Ann Oncol Off J Eur Soc Med Oncol. England; 2018;29:2121–8.

65. Martin M, Gonzalez-Rivera M, Morales S, de la Haba-Rodriguez J, Gonzalez-Cortijo L, Manso L, et al. Prospective study of the impact of the Prosigna assay on adjuvant clinical decision-making in unselected patients with estrogen receptor positive, human epidermal growth factor receptor negative, node negative early-stage breast cancer. Curr Med Res Opin. England; 2015;31:1129–37.

66. Fernandez-Martinez A, Pascual T, Perrone G, Morales S, de la Haba J, Gonzalez-Rivera M, et al. Limitations in predicting PAM50 intrinsic subtype and risk of relapse score with Ki67 in estrogen receptor-positive HER2-negative breast cancer. Oncotarget. United States; 2017;8:21930–7.

67. Cejalvo JM, Martinez de Duenas E, Galvan P, Garcia-Recio S, Burgues Gasion O, Pare L, et al. Intrinsic Subtypes and Gene Expression Profiles in Primary and Metastatic Breast Cancer. Cancer Res. United States; 2017;77:2213–21.

68. Prat A, Cheang MCU, Galvan P, Nuciforo P, Pare L, Adamo B, et al. Prognostic Value of Intrinsic Subtypes in Hormone Receptor-Positive Metastatic Breast Cancer Treated With Letrozole With or Without Lapatinib. JAMA Oncol. United States; 2016;2:1287–94.

69. Kogawa T, Yonemori K, Naito Y, Noguchi E, Shimizu C, Tamura K, et al. Phase 1/2, multicenter, non-randomized, open-label, multiple-dose first-in-human study of U3-1402 (anti-HER3 antibody drug conjugate) in subjects with HER3-positive metastatic breast cancer. J Clin Oncol. American Society of Clinical Oncology (ASCO); 2017;35:TPS1116–TPS1116.

70. Kogawa T, Yonemori K, Masuda N, Takahashi S, Takahashi M, Iwase H, et al. Single agent activity of U3-1402, a HER3-targeting antibody-drug conjugate, in breast cancer patients: Phase 1 dose escalation study. J Clin Oncol. American Society of Clinical Oncology (ASCO); 2018;36:2512–2512.

71. Jänne PA, Baik C, Su W-C, Johnson ML, Hayashi H, Nishio M, et al. Efficacy and Safety of Patritumab Deruxtecan (HER3-DXd) in EGFR Inhibitor–Resistant, *EGFR* -Mutated Non–Small Cell Lung Cancer. Cancer Discov. 2022;12:74–89.

72. Efficacy and safety of patritumab deruxtecan (U3-1402), a novel HER3 directed antibody drug conjugate, in patients (pts) with EGFR-mutated (EGFRm)... | OncologyPRO [Internet]. [cited 2021 Mar 17]. Available from: https://oncologypro.esmo.org/meeting-resources/esmo-virtual-congress-2020/efficacy-and-safety-of-patritumab-deruxtecan-u3-1402-a-novel-her3-directed-antibody-drug-conjugate-in-patients-pts-with-egfr-mutated-egfrm

73. Savas P, Salgado R, Denkert C, Sotiriou C, Darcy PK, Smyth MJ, et al. Clinical relevance of host immunity in breast cancer: from {TILs} to the clinic. 13:228–41.

74. Salgado R, Denkert C, Campbell C, Savas P, Nuciforo P, Aura C, et al. Tumor-Infiltrating Lymphocytes and Associations With Pathological Complete Response and Event-Free Survival in HER2-Positive Early-Stage Breast Cancer Treated With Lapatinib and Trastuzumab: A Secondary Analysis of the NeoALTTO Trial. JAMA Oncol. United States; 2015;1:448–54.

75. Solinas C, Carbognin L, De Silva P, Criscitiello C, Lambertini M. Tumor-infiltrating lymphocytes in breast cancer according to tumor subtype: Current state of the art. Breast Edinb Scotl. Netherlands; 2017;35:142–50.

76. Mao Y, Qu Q, Zhang Y, Liu J, Chen X, Shen K. The value of tumor infiltrating lymphocytes (TILs) for predicting response to neoadjuvant chemotherapy in breast cancer: a systematic review and meta-analysis. PloS One. United States; 2014;9:e115103.

77. Loi S, Michiels S, Salgado R, Sirtaine N, Jose V, Fumagalli D, et al. Tumor infiltrating lymphocytes are prognostic in triple negative breast cancer and predictive for trastuzumab benefit in early breast cancer: results from the FinHER trial. Ann Oncol. 2014;25:1544–50.

78. Loi S, Sirtaine N, Piette F, Salgado R, Viale G, Van Eenoo F, et al. Prognostic and predictive value of tumor-infiltrating lymphocytes in a phase III randomized adjuvant breast cancer trial in node-positive breast cancer comparing the addition of docetaxel to doxorubicin with doxorubicin-based chemotherapy: BIG 02-98. J Clin Oncol Off J Am Soc Clin Oncol. United States; 2013;31:860–7.

79. Stanton SE, Adams S, Disis ML. Variation in the Incidence and Magnitude of Tumor-Infiltrating Lymphocytes in Breast Cancer Subtypes: A Systematic ReviewTumor-Infiltrating Lymphocytes in Breast Cancer SubtypesTumor-Infiltrating Lymphocytes in Breast Cancer Subtypes. JAMA Oncol. 2016;2:1354–60.

80. Denkert C, Loibl S, Noske A, Roller M, Muller BM, Komor M, et al. Tumor-associated lymphocytes as an independent predictor of response to neoadjuvant chemotherapy in breast cancer. J Clin Oncol Off J Am Soc Clin Oncol. United States; 2010;28:105–13.

81. Dieci M V, Criscitiello C, Goubar A, Viale G, Conte P, Guarneri V, et al. Prognostic value of tumor-infiltrating lymphocytes on residual disease after primary chemotherapy for triple-negative breast cancer: a retrospective multicenter study. Ann Oncol. 2015;26:1518.

82. Criscitiello C, Bayar MA, Curigliano G, Symmans FW, Desmedt C, Bonnefoi H, et al. A gene signature to predict high tumor-infiltrating lymphocytes after neoadjuvant chemotherapy and outcome in patients with triple-negative breast cancer. Ann Oncol Off J Eur Soc Med Oncol. England; 2018;29:162–9.

83. Robertson JFR, Dowsett M, Bliss JM, Morden JP, Wilcox M, Evans A, et al. Abstract GS1-03: Peri-operative aromatase inhibitor treatment in determining or predicting longterm outcome in early breast cancer – The POETIC* Trial (CRUK/07/015). Cancer Res. 2018;78:GS1-03.

84. Nuciforo P, Pascual T, Cortes J, Llombart-Cussac A, Fasani R, Pare L, et al. A predictive model of pathologic response based on tumor cellularity and tumor-infiltrating lymphocytes (CelTIL) in HER2-positive breast cancer treated with chemo-free dual HER2 blockade. Ann Oncol Off J Eur Soc Med Oncol. England; 2018;29:170–7.

85. Prat A, Llombart-Cussac A, De La Peña L, Di Cosimo S, Ortega V, Rubio I, et al. Abstract OT3-3-06: NeoEribulin: A Phase II, non-randomized, open-label, single-arm, multicenter, exploratory pharmacogenomic study of single agent eribulin as neoadjuvant treatment for operable Stage I-II HER2 non-overexpressing breast cancer. Cancer Res. 2012;72:OT3-3.

86. Adamo B, Bellet M, Paré L, Pascual T, Vidal M, Pérez Fidalgo JA, et al. Oral metronomic vinorelbine combined with endocrine therapy in hormone receptor-positive HER2-negative breast cancer: SOLTI-1501 VENTANA window of opportunity trial. Breast Cancer Res. BioMed Central Ltd.; 2019;21:108.

87. Llombart-Cussac A, Cortés J, Paré L, Galván P, Bermejo B, Martínez N, et al. HER2-enriched subtype as a predictor of pathological complete response following trastuzumab and lapatinib without chemotherapy in early-stage HER2-positive breast cancer (PAMELA): an open-label, single-group, multicentre, phase 2 trial. Lancet Oncol. Lancet Publishing Group; 2017;18:545–54.

88. Prat A, Saura C, Pascual T, Hernando C, Muñoz M, Paré L, et al. Ribociclib plus letrozole versus chemotherapy for postmenopausal women with hormone receptor-positive, HER2-negative, luminal B breast cancer (CORALLEEN): an open-label, multicentre, randomised, phase 2 trial. Lancet Oncol. Lancet Publishing Group; 2020;21:33–43.

89. Prat A, Ortega V, Villagrasa P, Paré L, Galván P, Oliveira M, et al. Abstract P1-09-09: Efficacy and gene expression results from SOLTI1007 NEOERIBULIN phase II clinical trial in HER2-negative early breast cancer. Cancer Res. American Association for Cancer Research (AACR); 2017. p. P1-09-09-P1-09–09.

90. Ocana A, Vera-Badillo F, Seruga B, Templeton A, Pandiella A, Amir E. HER3 overexpression and survival in solid tumors: a meta-analysis. J Natl Cancer Inst. United States; 2013;105:266–73.

91. Chiu CG, Masoudi H, Leung S, Voduc DK, Gilks B, Huntsman DG, et al. HER-3 overexpression is prognostic of reduced breast cancer survival: a study of 4046 patients. Ann Surg. United States; 2010;251:1107–16.

92. Fuchs IB, Siemer I, Buhler H, Schmider A, Henrich W, Lichtenegger W, et al. Epidermal growth factor receptor changes during breast cancer metastasis. Anticancer Res. Greece; 2006;26:4397–401.

93. Hashimoto Y, Koyama K, Kamai Y, Hirotani K, Ogitani Y, Zembutsu A, et al. A Novel HER3-Targeting Antibody-Drug Conjugate, U3-1402, Exhibits Potent Therapeutic Efficacy through the Delivery of Cytotoxic Payload by Efficient Internalization. Clin Cancer Res Off J Am Assoc Cancer Res. United States; 2019;

94. Yonemori K, Masuda N, Takahashi S, Kogawa T, Nakayama T, Yamamoto Y, et al. 151O Single agent activity of U3-1402, a HER3-targeting antibody-drug conjugate, in HER3-overexpressing metastatic breast cancer: Updated results from a phase I/II trial. Ann Oncol. Oxford University Press; 2019;30:mdz100-002.

95. González-Farré B, NP, BLP, CJ, CAL, GJG, et al. (2020). 15P The CelTIL score as an early predictor of anti-tumour response following neoadjuvant therapy (NAT): A SOLTI biomarker analysis. Ann Oncol 31 S22. 2020;

19. Appendices

19.1 Appendix A Table of assessments

Assessments	Screening		C1D1	C1D3-D7	C1D21	End of Study (EOS[p])	Eight Month Follow-up
**Days**	**>42 days**	**-42 to 1**	**1**	**3-10 days**	**21(±3 days)**	**49(±14 days)**	**10 months (±60 days)**
Informed consent [a]	X						
Inclusion / exclusion criteria [k]		X					
Complete anamnesis [b]		X					
Physical examination [c]		X				X	
Vital signs and weight [d]		X	X		X	X	
Height		X					
TNM classification [e]		X					
Clinical evaluation of the tumor: breast & axillar examination [f]		X	X		X		
ECOG status		X	X		X	X	
12-lead ECG		X					
LVEF		X					
Breast and axillar ultrasound		X			X		
ER/PgR, HER2, Ki67 status by local laboratory		X					
Pregnancy test[o]		X	X				
Hemogram with formula and platelet count [i]		X	X		X	X	
Fasting serum biochemistry [j]		X	X		X	X	
Coagulation (INR y aPTT)		X	X				
Tumor samples collection	X [g]			X[r]	X[h]		
ERBB3 mRNA expression by central lab		X					
Medical Monitor Validation		X					
Assignment [l]			X				
Study treatment administration (U3-1402)			X				
Concomitant medication [m] & AEs		X	X		X	X	
NAC/Neoadjuvant treatment & surgery outcome						X[q]	X[q]
Blood samples for biomarkers [n]			X	X[s]	X	X	
AES and SAES Collection			X		X	X	

AEs = Adverse Events; aPTT – activated partial thromboplastin time; ECG – Electrocardiogram; ECOG - Eastern Cooperative Oncology Group; INR – International normalized ratio; ULN - upper limit of normal; Soc – Standard of care. Clarification: All procedures on C1D1 visit must be done pre-dose, unless otherwise specified.

a Written informed consent is required before performing any study-specific tests or procedures. Signing of the Informed Consent Form can occur outside the 42-day screening period. All screening evaluations must be completed and reviewed to confirm that patients meet all eligibility criteria before enrollment. Results of standard-of-care tests or examinations performed prior to obtaining informed consent and within 42 days prior to study entry (except where otherwise specified) may be used for screening assessments rather than repeating such tests. The investigator will maintain a screening log to record details of all patients screened and to confirm eligibility or record reasons for screening failure, as applicable.

b Anamnesis complete, with demographic data (age, sex and race or ethnic group), reproductive status, clinically significant diseases in the last 5 years, surgeries, toxic background (smoking, alcohol or drug abuse), oncological history (including treatments and previous procedures against cancer), history of breast cancer with tumor characteristics (namely, date of diagnosis, treatments and antineoplastic procedures, type and histological grade,% estrogen and progesterone receptor, HER2 status,% Ki67) and complete cardiovascular history.

c Baseline physical examination will be performed in the 7 days prior to assignation and must include evaluation of the head, mouth, eyes, ears, nose and throat, neck (including thyroid), abdomen, back, extremities, lymph nodes, and cardiovascular, dermatological, locomotor, respiratory, digestive, genitourinary and neurological systems, weight and height. If no abnormal data are observed during selection, the full physical examination may be based on concurrent signs and/or symptoms.

d The vital signs are body temperature, systolic and diastolic blood pressure in supine position, and heart rate. If this determination has been made within 7 days prior to treatment assignation, it will not be necessary to repeat it on day 1.

e Lymph node stage will be determined by ultrasound. For patients with suspicious axillary lymph nodes, axillary staging should include fine needle aspiration biopsy or core needle biopsy guided by ultrasound performed within 42 days prior to the start of treatment. Distant disease should be discarded by local practice distance according to local practice.

f Measurement with gauge or rule, and preferably performed by the same evaluator throughout the study. If this determination has been made within 7 days prior to treatment assignation, it will not be necessary to repeat it on day 1. Then will be performed again at C1D21.

g A window longer than 42 days is allowed. If archival tissue is either insufficient or unavailable, a new biopsy from the pretreatment tumor must be obtained. The biopsy will only be done if the tumor is larger than 1 cm in its greatest diameter by ultrasound, otherwise, the patient will not be eligible. Tumor tissue samples should be submitted in the form of paraffin blocks, at least two tissue cylinders with enough material to do at least: one 3 µm slide, nine 4 µm slides, and eighteen 10 µm slides, this slides should be provided in TOMO slides and stored at 2-8ºC.

h 2 Core biopsy + 5 mg of fresh frozen tissue must be obtained at C1D21; the equivalent in tissue for 2 cores biopsies samples should be submitted in the form of paraffin blocks (both in same block) flash frozen tumor tissue should be submitted frozen.

i The blood count includes a count of erythrocytes, hemoglobin, hematocrit, leukocyte count with formula (neutrophils, eosinophils, basophils, lymphocytes, monocytes and other cells) and platelet count. Screening results may be valid for C1D1 if performed within 7 days prior to Cycle 1, Day 1.

j Fasting serum biochemistry (≥ 8 hours fasting): sodium, potassium, calcium, glucose, creatinine, total protein, albumin, total bilirubin, direct bilirubin if total bilirubin is > ULN, alkaline phosphatase, LDH, AST, ALT and GGTP. Screening results may be valid for C1D1 if performed within 7 days prior to Cycle 1, Day 1.

k Confirmation that the patient meets all the inclusion criteria and none of exclusion before their inclusion in the study.

l Once included, the patient must start the assigned treatment that same day or within 3 days.

m All medication taken during the 7 days prior to screening, including supplements, prescription and over-the-counter medications, and phytotherapy/homeopathy, will be recorded.

n In all cases, 30ml peripheral blood will be collected aseptically by venipuncture into sterile EDTA-treated tubes for ctDNA analysis. Furthermore, for future research in each time point at C1D1 (can be extracted during screening if it is done up to 7 days before C1D1), C1D21 visit and EOS. Blood sample (10 ml) for HER3 mRNA will be obtained at baseline, C1D3-D7 and C1D21 (only if missing C1D21 sample, an EOS sample will be collected). While DXd (5 ml) will be collected after dosing at C1D3-D7 and C1D21.

o A urine or serum β-HCG test must be performed for women of childbearing potential (including pre-menopausal women who have had a tubal ligation). Testing should be performed within 7 days prior to the first administration of study medication. For all other women, documentation must be present in medical history confirming that the patient is not of childbearing potential. If the urine pregnancy test is positive or cannot be confirmed as negative, a serum pregnancy test will be required.

p A safety follow-up will be done at 49 days (+/- 14 days) after treatment administration. Follow-up of AEs related to the study medication will be carried out until its resolution, with analytics if necessary.

q Information on surgery or neoadjuvant or adjuvant treatment received will be collected, At 10-months. Follow-up response to chemotherapy will be collected as well as surgery outcome (pathological staging) if performed after EOS/SFV follow-up.

r Optional biopsy. The optional biopsy will be used for analysis of HER3 IHC, γH2AX IHC, Ki67 IHC, DXd concentration. 1 core needle biopsy (in paraffin block) plus 5 mg of flash frozen tissue is needed. This biopsy will be taken only if the tumor is larger or equal than 2 cm.

s Blood and tumor sample can be taken between Day 3 to day 10 including both.

19.2 Appendix B– Acceptable methods of contraception

- Combined (estrogen and progestogen containing) hormonal contraception associated with inhibition of ovulation (oral, intravaginal, or transdermal delivery).
- Progestogen-only hormonal contraception associated with inhibition of ovulation (oral, injectable, or implantable delivery).
- Intrauterine device (IUD).
- Intrauterine hormone-releasing system (IUS).
- Bilateral tubal occlusion.
- Vasectomized partner.
- Complete sexual abstinence.

19.3 Appendix C – ECOG performance status

Grade	ECOG
**0**	Fully active, able to carry on all pre-disease performance without restriction.
**1**	Restricted in physically strenuous activity but ambulatory and able t carry out work of a light or sedentary nature, e.g., light housework, offic work.
**2**	Ambulatory and capable of all self-care but unable to carry out any work activities.<br>Up and about more than 50% of waking hours.
**3**	Capable of only limited self-care, confined to bed or chair more than 50% of waking hours.
**4**	Completely disabled.<br>Cannot carry on any self-care. Totally confined to bed or chair.
**5**	Dead.

19.4 Appendix D- Adverse Events of Special Interest

Adverse events of special interest are required to be reported by the investigator to the Sponsor immediately (i.e., no more than 24 hours after learning of the event) using the relevant CRFs. For the U3-1402 clinical program, based on the available nonclinical data, review of the cumulative literature, reported toxicities for the same class of agents of the monoclonal antibody, and payload of U3-1402 and biological plausibility, the events described below are considered to be AEs of special interest:

- Interstitial Lung Disease
- Pneumonitis
- Potential Hy's Law

Cases of potential drug-induced liver injury that include an elevated ALT or AST in combination with either elevated bilirubin or clinical jaundice, as defined by Hy's Law and based on the following observations:

  - Treatment-emergent ALT or AST > 3 ´ ULN (or > 3 ´ baseline value in disease states where LFTs may be elevated at baseline) in combination with total bilirubin > 2 ´ ULN (of which ^{3} 35% is direct bilirubin)
  - Treatment-emergent ALT or AST > 3 ´ ULN (or > 3 ´ baseline value in disease states where LFTs may be elevated at baseline) in combination with clinical jaundice.

19.5 Appendix E- Modified Response Evaluation Criteria in Solid Tumors: Assessment of Response of Neoadjuvant Therapy in Early Breast Cancer

Conventional response criteria may not be ideal for the assessment of response in the setting of neoadjuvant therapy in early breast cancer. Therefore, RECIST 1.1 criteria have been modified to specifically address assessment of primary breast lesions along with axillary lymph node disease, using ultrasound. Selected sections from the Response Evaluation Criteria in Solid Tumors (RECIST), Version 1.1 (Eisenhauer et al., 2009) are presented below, with modifications and the addition of explanatory text as needed for clarity.

Table 1: Modified RECIST Early Breast Cancer Neoadjuvant Therapy

	RECIST v1.1	Modified RECIST
Modalities	CT as primary modality, ultrasound not recommended	Primary modality ultrasound, and clinical exam
Lymph nodes	May be considered target lesions based on size criteria (15 mm in SAD.	Only axillary lymph nodes assessed; nodes that are considered abnormal on imaging (based on morphological factors including, but not limited to SAD) to be followed as non-target lesions
Possibility of having only non-target disease	Allowed	Not allowed; primary breast lesions must be measurable by ultrasound

CT = computed tomography;; SAD = short axis dimension.

Baseline Documentation of Target and Non-Target Lesions

To assess objective response or future progression, it is necessary to estimate the overall tumor burden at baseline and to use this as a comparator for subsequent measurements.

All baseline evaluations should be performed as close as possible to the treatment start.

Target Lesions

Target lesions should be selected on the basis of their size (lesions with the longest diameter) and should lend themselves to reproducible repeated measurements. Up to 2 lesions in the breast may be identified as target lesions. *Per this protocol, in the case of a multicentric/multifocal tumor, the largest lesion must be ≥10 mm and designated as target lesion for all subsequent evaluations.* A sum of the diameters of all target lesions will be calculated and reported as the baseline sum of diameters. The baseline sum of diameters will be used as a reference to further characterize any objective tumor regression in the measurable dimension of the disease. Lesions that meet the criteria for radiographically defined simple cysts should not be considered malignant lesions (neither target nor non-target) since they are, by definition, simple cysts. Pathologic axillary lymph nodes are not to be designated at target lesions, and lymph node measurements are not to be included in the sum of diameters (see below for more detail).

Bilateral breast imaging studies should be conducted at each protocol-specified study assessment. The same method of measurement and the same technique should be used to characterize each target lesion at baseline and during the study, and all measurements should be recorded in metric notation. Care must be taken in measurement of target lesions with different modalities, since the same lesion may appear to have a different size with each modality. If for some reason the same imaging modality cannot be used at a scheduled assessment timepoint, then the case should be discussed with the radiologist to determine if substitution of any other approach is possible and, if not, the patient should be considered not evaluable at that timepoint, for that particular type of imaging assessment.

Non-Target Lesions

Non-target lesions may include any other measurable breast lesions not identified as target lesions, as well as truly non-measurable lesions, such as diffuse skin thickening or other lesions not measurable by reproducible imaging techniques.

Lymph nodes merit special mention since they are normal anatomical structures that may be visible by imaging even if not involved by tumor. Axillary lymph nodes are known to vary widely in size, and signs of abnormality in axillary lymph nodes on imaging include other morphological

findings often in addition to changes in nodal size. For these reasons, pathologic axillary lymph nodes on imaging should be identified as non-target lesions at baseline. Change in short-axis dimension may be considered in the assessment of pathology, but measurements are not required, and these lesions should be followed qualitatively, as described below at each response assessment timepoint.

Signs of lymph node pathology on imaging include the following:

- Increase in short axis dimension
- Thickened cortex, either diffusely or asymmetrically enlarged
- Thinning, or replaced fatty hilum
- Irregular margins or spiculations
- Rim enhancement
- Decreased echogenicity of cortex
- Perinodal edema

Evaluation of response

Evaluation of Target Lesions

This section provides the definitions of the criteria used to determine objective tumor response for target breast lesions:

- Complete response (CR): disappearance of all target lesions
- Partial response (PR): at least a 30% decrease in the sum of diameters of target lesions, taking as reference the baseline sum of diameters
- Progressive disease (PD): at least a 20% increase in the sum of diameters of target lesions, taking as reference the smallest sum on study (nadir), including baseline, AND an absolute increase of ≥5 mm. The appearance of one or more new lesions is also considered progression.
- Stable disease (SD): neither sufficient shrinkage to qualify for PR nor sufficient increase to qualify for PD, taking as reference the smallest sum on study
-

Special Notes on the Assessment of Target Lesions

- **Target Lesions That Become Too Small to Measure.** While on study, all lesions recorded

at baseline should have their actual measurements recorded at each subsequent evaluation, even when very small (e.g., 2 mm). However, sometimes lesions that are recorded as target lesions at baseline become so faint on imaging that the radiologist may not feel comfortable assigning an exact measure and may report them as being too small to measure. When this occurs, it is important that a value be recorded on the CRF as follows:

- If it is the opinion of the radiologist that the lesion has likely disappeared, the measurement should be recorded as 0 mm.
- If the lesion is believed to be present and is faintly seen but too small to accurately measure, BML (below measurable limit) should be indicated.

To reiterate, however, if the radiologist is able to provide an actual measure, that should be recorded, and, in that case, BML should not be ticked.

- **Lesions That Split or Coalesce on Treatment.** When non-nodal lesions fragment, the longest diameters of the fragmented portions should be added together to calculate the target lesion sum. Similarly, as lesions coalesce, a plane between them may be maintained that would aid in obtaining maximal diameter measurements of each individual lesion. If the lesions have truly coalesced such that they are no longer separable, the vector of the longest diameter for the coalesced lesion should be recorded.

Evaluation of Non-Target Lesions

This section provides the definitions of the criteria used to determine the tumor response for any non-target lesions identified at baseline. Although some non-target lesions may actually be measurable, they need not be measured and, instead, should be assessed only qualitatively at the timepoints specified in the protocol.

- CR: disappearance of all non-target lesions. All lymph nodes must be non-pathologic in appearance
- Non-CR/Non-PD: persistence of one or more non-target lesion(s)
- PD: unequivocal progression of existing non-target lesions. For pathologic axillary lymph nodes, this may be based on a combination of morphological

factors, including a potential increase in short-axis dimension

Special Notes on Assessment of Progression of Non-Target Disease

To achieve unequivocal progression on the basis of the non-target disease, there must be an overall level of substantial worsening in non-target disease in a magnitude that, even in the presence of SD or PR in target disease, the overall tumor burden has increased sufficiently to merit discontinuation of therapy. A modest increase in the size of one or more non-target lesions is usually not sufficient to qualify for unequivocal progression status. The designation of overall progression solely on the basis of change in non-target disease in the face of SD or PR of target disease will therefore be extremely rare.

New Lesions

The appearance of new malignant lesions denotes disease progression; therefore, some comments on detection of new lesions are important. There are no specific criteria for the identification of new radiographic lesions; however, the finding of a new lesion should be unequivocal, that is, not attributable to differences in scanning technique, change in imaging modality, or findings thought to represent something other than tumor. This is particularly important when the patient's baseline lesions show partial or complete response. For example, necrosis of a breast lesion may be reported on an MRI scan report as a "new" cystic lesion, which it is not. A lesion identified during the study in an anatomical location that was not scanned at baseline is considered a new lesion and will indicate disease progression.

If a new lesion is equivocal, for example because of its small size, continued therapy and follow-up evaluation will clarify if it represents truly new disease. If repeat scans confirm there is definitely a new lesion, then progression should be declared using the date of the initial scan.

Timepoint Response (Overall Response)

Table 2 provides a summary of the overall response status calculation at each protocol-specified timepoint for which a response assessment occurs.

Missing Assessments and Not-Evaluable Designation

When no imaging/measurement is done at all at a particular timepoint, the patient is not evaluable at that timepoint. If only a subset of lesion measurements are made at an assessment, usually the case is also considered not evaluable at that timepoint, unless a convincing argument can be made that the contribution of the individual missing lesion(s) would not change the assigned timepoint response. Similarly, if one or more non-target lesions are not assessed, the response for non-target lesions should be "not evaluable" except where there is clear progression in non-target lesions that are assessed.

Special Notes on Response Assessment

Patients with a global deterioration of health status requiring discontinuation of treatment without objective evidence of disease progression at that time should be reported as "symptomatic deterioration." Every effort should be made to document objective progression even after discontinuation of treatment. Symptomatic deterioration is not a descriptor of an objective response; it is a reason for stopping study therapy. The objective response status of such patients is to be determined by evaluation of target and non-target disease as shown in Table 2.

Table 2. Integration of target, non-target and new lesions into response assessment.

Target Lesions	Non-Target Lesions	New Lesions	Overall Response
CR	CR	No	CR
CR	Non-CR/Non-PD	No	PR
CR	Not all evaluated	No	PR
PR	Non-PD/ not all evaluated	No	PR
SD	Non-PD/ not all evaluated	No	SD
Not all evaluated	Non-PD	No	NE
PD	Any	Any	PD
Any	PD	Any	PD
Any	Any	Yes	PD
No Target	CR	No	CR
No Target	Non-CR/non-PD	No	Non-CR/ non-PD
No Target	Not all evaluated	No	NE
No Target	Unequivocal PD	Any	PD
No Target	Any	Yes	PD

References:Eisenhauer, E.A., P. Therasse, J. Bogaerts, L.H. Schwartz, D. Sargent, R. Ford, J. Dancey, et al. «New Response Evaluation Criteria in Solid Tumours: Revised RECIST Guideline (Version 1.1)». *European Journal of Cancer* 45, n.º 2 (enero de 2009): 228-47. https://doi.org/10.1016/j.ejca.2008.10.026.
