# Supplementary material for: Patritumab deruxtecan in HER2-negative breast cancer: part B results of the window-of-opportunity SOLTI-1805 TOT-HER3 trial and biological determinants of early response
Source: Nat Commun. 2024 Jul 11;15:5826. doi: 10.1038/s41467-024-50056-y (PMC11239918; doi:10.1038/s41467-024-50056-y)
Supplement: Supplementary file 3 — Description of Additional Supplementary Files [file 41467_2024_50056_MOESM3_ESM.pdf]

Description of Additional Supplementary Files

Supplementary Data 1. Pathological data from SOLTI-1805 TOT-HER3 part A including ER (%), PR(%), HER2 IHC, absolute CelTIL change between C1D21 and baseline, and PAM50 subtype.

Supplementary Data 2. Gene expression counts from SOLTI-1805 TOT-HER3 part A samples.

Supplementary Data 3. DNA-based subtypes and signatures from SOLTI-1805 TOT-HER3 part A samples.

Supplementary Data 4. Mutation data from SOLTI-1805 TOT-HER3 part A samples.

Supplementary Data 5. Pathological data from SOLTI-1805 TOT-HER3 part B including ER (%), PR(%), HER2 IHC, absolute CelTIL change between C1D21 and baseline, and PAM50 subtype

Supplementary Data 6. Gene expression counts from SOLTI-1805 TOT-HER3 part B.
