# Supplementary material for: Patritumab deruxtecan in HER2-negative breast cancer: part B results of the window-of-opportunity SOLTI-1805 TOT-HER3 trial and biological determinants of early response
Source: Nat Commun. 2024 Jul 11;15:5826. doi: 10.1038/s41467-024-50056-y (PMC11239918; doi:10.1038/s41467-024-50056-y)
Supplement: Supplementary file 10 — Reporting Summary [file 41467_2024_50056_MOESM10_ESM.pdf]

nature portfolio

Corresponding author(s): Aleix Prat

Last updated by author(s): Jun 7, 2024

Reporting Summary

Nature Portfolio wishes to improve the reproducibility of the work that we publish. This form provides structure for consistency and transparency in reporting. For further information on Nature Portfolio policies, see our Editorial Policies and the Editorial Policy Checklist.

Statistics

For all statistical analyses, confirm that the following items are present in the figure legend, table legend, main text, or Methods section.

n/a	Confirmed	
☐	☒	The exact sample size (*n*) for each experimental group/condition, given as a discrete number and unit of measurement
☒	☐	A statement on whether measurements were taken from distinct samples or whether the same sample was measured repeatedly
☐	☒	The statistical test(s) used AND whether they are one- or two-sided<br>*Only common tests should be described solely by name; describe more complex techniques in the Methods section.*
☐	☒	A description of all covariates tested
☐	☒	A description of any assumptions or corrections, such as tests of normality and adjustment for multiple comparisons
☐	☒	A full description of the statistical parameters including central tendency (e.g. means) or other basic estimates (e.g. regression coefficient) AND variation (e.g. standard deviation) or associated estimates of uncertainty (e.g. confidence intervals)
☐	☒	For null hypothesis testing, the test statistic (e.g. *F*, *t*, *r*) with confidence intervals, effect sizes, degrees of freedom and *P* value noted<br>*Give P values as exact values whenever suitable.*
☐	☐	For Bayesian analysis, information on the choice of priors and Markov chain Monte Carlo settings
☐	☒	For hierarchical and complex designs, identification of the appropriate level for tests and full reporting of outcomes
☐	☒	Estimates of effect sizes (e.g. Cohen's *d*, Pearson's *r*), indicating how they were calculated

Our web collection on statistics for biologists contains articles on many of the points above.

Software and code

Policy information about availability of computer code

Data collection	no software used
Data analysis	R software v 4.0.3

For manuscripts utilizing custom algorithms or software that are central to the research but not yet described in published literature, software must be made available to editors and reviewers. We strongly encourage code deposition in a community repository (e.g. GitHub). See the Nature Portfolio guidelines for submitting code & software for further information.

Data

Policy information about availability of data

All manuscripts must include a data availability statement. This statement should provide the following information, where applicable:

- Accession codes, unique identifiers, or web links for publicly available datasets
- A description of any restrictions on data availability
- For clinical datasets or third party data, please ensure that the statement adheres to our policy

The protocol of the SOLTI-1805 TOT-HER3 study is available as a Supplementary Information file. The data generated in this study including gene expression counts, PAM50 subtypes, DNA signatures and subtypes, and pathological data can be found in Supplementary Material. FASTQ and BAM files from targeted DNA-Seq experiments of 49 breast cancer samples have been submitted to EGA European Genome-Phenome Archive under the accession number EGAD50000000562 [https://ega-archive.org/datasets/EGAD50000000562]. Data are available under restricted access as participants of this study did not agree for their sequencing

data to be shared publicly. Access can be obtained for academic use only, under a data transfer agreement and upon Ethics Committee approval. The timescale for this process is approximately 6 months and the data will be available for 3 years. . GSE25066 gene expression and pCR data was downloaded from the Gene Expression Omnibus (GEO) under the accession number GSE25066 [https://www.ncbi.nlm.nih.gov/geo/query/acc.cgi?acc=gse25066]. METABRIC data was downloaded from cBioportal [https://www.cbioportal.org/study/summary?id=brca_metabric]. The data generated in this study and presented in the figures are provided in the Supplementary Data/Source Data files. Source data are provided with this paper.

Research involving human participants, their data, or biological material

Policy information about studies with human participants or human data. See also policy information about sex, gender (identity/presentation), and sexual orientation and race, ethnicity and racism.

Reporting on sex and gender	Reported in Supplementary Tables
Reporting on race, ethnicity, or other socially relevant groupings	Reported in Supplementary Tables
Population characteristics	Reported in Supplementary Tables
Recruitment	For SOLTI-1805 TOT-HER3 Part B, a total of 44 patients were assessed for eligibility, and 37 patients were included in the study. Recruitment period was 10 months.
Ethics oversight	Written informed consent was obtained from all study participants before the initiation of any study-specific assessments. This trial was conducted in compliance with the protocol, regulatory requirements, an independent ethics committee in accordance with the International Council for Harmonisation of Technical Requirements for Pharmaceuticals for Human Use guidelines for Good Clinical Practice, and the ethical principles of the latest revision of the Declaration of Helsinki as adopted by the World Medical Association and approved by the independent ethics committee of Hospital Clínico de Valencia and the the Spanish Agency for Medicines and Health Products.

Note that full information on the approval of the study protocol must also be provided in the manuscript.

Field-specific reporting

Please select the one below that is the best fit for your research. If you are not sure, read the appropriate sections before making your selection.

☒ Life sciences ☐ Behavioural & social sciences ☐ Ecological, evolutionary & environmental sciences

For a reference copy of the document with all sections, see nature.com/documents/nr-reporting-summary-flat.pdf

Life sciences study design

All studies must disclose on these points even when the disclosure is negative.

Sample size	Part B of the study target was to include 20 patients with HR+/HER2-negative breast cancer and 15 patients with TNBC to be treated with a dose of 5.6 mg/kg. No formal sample size calculation was made, but it maintained a reasonable proportion with Part A, also allowing recruitment within a reasonable time frame.<br>For the translational study we did not perform a sample size calculation and we used all the samples available for correlative analyses.
Data exclusions	1. Inoperable locally advanced or inflammatory (i.e., inoperable Stage III) breast cancer.<br>2. Metastatic (Stage IV) breast cancer.<br>3. Bilateral invasive breast cancer.<br>4. Patients in whom a primary tumor excisional biopsy was performed.<br>5. Any prior treatment for primary actual invasive breast cancer.<br>6. Prior treatment with a HER3 antibody, topoisomerase I inhibitor, with an ADC which consists of an exatecan derivative that is a topoisomerase I inhibitor (e.g., DS-8201) and with a govitecan derivative (e.g., IMMU-132).<br>7. Medical history of symptomatic congestive heart failure (New York Heart Association classes II-IV) or serious cardiac arrhythmia requiring treatment; myocardial infarction within 6 months prior to enrollment or unstable angina.<br>8. QT interval corrected using Fridericia's formula to > 450 millisecond (ms) in males and > 470 ms in females.<br>9. Any factors that increase the risk of corrected QT (QTc) interval prolongation or risk of arrhythmic events, such as congenital long QT syndrome, family history of long QT syndrome, or unexplained sudden death under 40 years of age in first-degree relatives.<br>10. Medical history of clinically significant lung diseases (e.g., interstitial pneumonia, pneumonitis, pulmonary fibrosis, and severe radiation pneumonitis) or who are suspected to have these diseases by imaging at screening period.<br>11. Clinically significant corneal disease.<br>12. Major surgical procedure or significant traumatic injury within 28 days prior to enrollment.<br>13. Assessment by the investigator to be unable or unwilling to comply with the requirements of the protocol.<br>14. History of other malignancy within the last 3 years, except for appropriately treated carcinoma in situ of the cervix, non-melanoma skin carcinoma, Stage I uterine cancer, or other malignancies with an expected curative outcome.<br>15. Current severe, uncontrolled systemic disease (e.g. clinically significant cardiovascular, pulmonary or metabolic disease; wound healing disorders; ulcers; bone fractures).<br>16. Concurrent, serious, uncontrolled infections or current known infection with HIV or active hepatitis B and/or hepatitis C.<br>17. History of significant co-morbidities that, in the judgment of the investigator, may interfere with the conduction of the study, the evaluation of response, or with ICF.

18. Known hypersensitivity to either the drug substance components (including an antibody, a drug-linker, or a topoisomerase I inhibitor) or inactive ingredients in the drug product or history of severe hypersensitivity reactions to other monoclonal antibodies.
19. Clinically severe pulmonary compromise resulting from intercurrent pulmonary illnesses including, but not limited to, any underlying pulmonary disorder (i.e. pulmonary emboli within three months of the study enrollment, severe asthma, severe COPD, restrictive lung disease, pleural effusion etc.), and any autoimmune, connective tissue or inflammatory disorders with potential pulmonary involvement (i.e. rheumatoid arthritis, Sjögren's syndrome, sarcoidosis etc.), or prior pneumonectomy.
20. Has unresolved toxicities from previous anticancer therapy, defined as toxicities (other than alopecia) not yet resolved to National Cancer Institute Common Terminology Criteria for Adverse Events (NCI-CTCAE) version 5.0, grade ≤1 or baseline. Subjects with chronic grade 2 toxicities may be eligible per the discretion of the Investigator.
21. Is receiving chronic systemic corticosteroids dosed at >10 mg prednisone or equivalent anti-inflammatory activity or any form of immunosuppressive therapy prior to Cycle 1 Day 1. Subjects who require use of bronchodilators, inhaled or topical steroids, or local steroid injections may be included in the study.

Replication	The translational analyses were repeated at least twice and were performed by two investigators.
Randomization	N/A
Blinding	Blinding was not relevant to the translational study, the information of all variables was needed to be available to perform correlative science analyses.

Reporting for specific materials, systems and methods

We require information from authors about some types of materials, experimental systems and methods used in many studies. Here, indicate whether each material, system or method listed is relevant to your study. If you are not sure if a list item applies to your research, read the appropriate section before selecting a response.

Materials & experimental systems

n/a	Involved in the study
☒	☐ Antibodies
☒	☐ Eukaryotic cell lines
☒	☐ Palaeontology and archaeology
☒	☐ Animals and other organisms
☐	☒ Clinical data
☒	☐ Dual use research of concern
☒	☐ Plants

Methods

n/a	Involved in the study
☒	☐ ChIP-seq
☒	☐ Flow cytometry
☒	☐ MRI-based neuroimaging

Clinical data

Policy information about clinical studies
All manuscripts should comply with the ICMJE guidelines for publication of clinical research and a completed CONSORT checklist must be included with all submissions.

Clinical trial registration	NCT04610528
Study protocol	The trial protocol can be accessed
Data collection	Pre- and post-menopausal women or men with untreated, operable breast cancer ≥1 cm by ultrasound. HER2-negative by local assessment. Ki67≥10% by local assessment.

Outcomes

Primary endpoint was CelTIL score
Mean change in CelTIL score per central assessment between paired samples (baseline and C1D21). CelTIL score = −0.8 × tumor cellularity (in %) + 1.3 × TILs (in %).

Secondary outcomes
CelTIL score [Time Frame: baseline and after one dose of U3-1402 at Cycle 1 Day 21]
Mean change in CelTIL score per central assessment in paired samples for each ERBB3 cohort
ERBB3 levels vs. CelTIL score [Time Frame: baseline and after one dose of U3-1402 at Cycle 1 Day 21]
ERBB3 mRNA baseline levels and changes in CelTIL score for all patients and for each ERBB3 cohort
HER3 IHC vs. CelTIL score [Time Frame: baseline and after one dose of U3-1402 at Cycle 1 Day 21]
HER3 IHC baseline levels and changes in CelTIL score for all patients and for each ERBB3 cohort
PAM50 intrinsic subtypes vs. CelTIL score [Time Frame: baseline and after one dose of U3-1402 at Cycle 1 Day 21]
changes in CelTIL across the four PAM50 intrinsic subtypes.
Complete Cell Cycle Arrest (CCCA) [Time Frame: baseline and after one dose of U3-1402 at Cycle 1 Day 21]
determined per central assessment by IHC Ki67< 2.7%
ERBB3 mRNA expression vs. HER3 IHC [Time Frame: baseline]
Correlation coefficients between both biomarkers
safety and tolerability [Time Frame: through study completion, an average of 65 days each patient]
Type, incidence, severity (as graded by the NCI CTCAE v. 5.0), seriousness and attribution to the study medications of AEs and any

laboratory abnormalities.
changes of HER3 expression. [Time Frame: t baseline, at Day 3-Day 7 (optional), Cycle 1 Day 21]
HER3 expression

Plants

Seed stocks

Report on the source of all seed stocks or other plant material used. If applicable, state the seed stock centre and catalogue number. If plant specimens were collected from the field, describe the collection location, date and sampling procedures.

Novel plant genotypes

Describe the methods by which all novel plant genotypes were produced. This includes those generated by transgenic approaches, gene editing, chemical/radiation-based mutagenesis and hybridization. For transgenic lines, describe the transformation method, the number of independent lines analyzed and the generation upon which experiments were performed. For gene-edited lines, describe the editor used, the endogenous sequence targeted for editing, the targeting guide RNA sequence (if applicable) and how the editor was applied.

Authentication

Describe any authentication procedures for each seed stock used or novel genotype generated. Describe any experiments used to assess the effect of a mutation and, where applicable, how potential secondary effects (e.g. second site T-DNA insertions, mosiacism, off-target gene editing) were examined.
